# Synthesis and quantum crystallographic evaluation of WYLID: YLID’s red rival

**DOI:** 10.1107/S160057672500175X

**Published:** 2025-04-04

**Authors:** Florian Meurer, Maximilian Schimpf, Birgit Hischa, Christoph Hennig, Julia Rehbein, Florian Kleemiss, Michael Bodensteiner

**Affiliations:** a Faculty for Chemistry and Pharmacy University of Regensburg Universitätsstraße 31 Regensburg93053 Germany; b Institute of Resource Ecology Helmholtz-Zentrum Dresden-Rossendorf (HZDR) Bautzner Landstraße 400 Dresden Sachsen01314 Germany; c Rossendorf Beamline European Synchrotron Radiation Facility Avenue des Martyrs 51 Grenoble Rhône-Alpes France; d Institute for Inorganic Chemistry RWTH Aachen Landoltweg 1a Aachen52074 Germany; University of Warsaw, Poland

**Keywords:** quantum crystallography, synthesis, chemical crystallography, complementary bond analysis

## Abstract

The synthesis and characterization of WYLID [2-(di­methyl-λ^4^-sulfane­ylidene)-[1,2′-bi­indenyl­idene]-1′,3,3′(2*H*)-trione], a side product that appears during the synthesis of YLID, is presented in this study. Quantum crystallographic insights into the bonding situation in WYLID are provided on the basis of Hirshfeld atom refinement (HAR), multipolar modelling and X-ray restrained wavefunction fitting. We compare these results with theoretical calculations on a gas-phase optimized density functional theory and a Hartree–Fock calculation based on the geometry obtained by HAR. The S—C bond in WYLID is best described as an ylid-type bond and the SMe_2_ fragment does not affect the nearest C—O carbonyl/enolate equilibrium. This work may contribute to an ongoing debate in the quantum crystallography community about the bonding situation in YLID and provides a routine for distinguishing between carbonyls and enolates.

## Abstract 2

The synthesis and quantum crystallographic analysis of the chemical bonding within WYLID, 2-(di­methyl-λ^4^-sulfane­ylidene)-[1,2′-bi­indenyl­idene]-1′,3,3′(2*H*)-trione, a condensation product of YLID which is the most widely used calibrant for laboratory diffractometers, is presented. An ylid-type description of the S—C bond and a carbonyl-type description of the C—O bonds in WYLID is found in all aspects of the analysis.

## 1. Introduction

Since its inception in the year 1912 (Friedrich *et al.*, 1912 ▸), X-ray crystallography has become the primary method for definitive structural elucidation. The continuous advancement in X-ray sources, goniometers and detectors has facilitated tremendous growth of X-ray crystallography over the past century, leading to its current position as a dominant technique. We recently demonstrated what extensive chemical information can be obtained nowadays using diffraction data from a state-of-the-art in-house diffractometer on the example of Cp′′′NiP_3_ (Cp′′′ = 1,2,4-tri-*tert*-butylcyclopentadienyl) and P_4_ in the context of Hoffmann’s isolobal principle (Meurer *et al.*, 2024 ▸).

**Figure 1:**
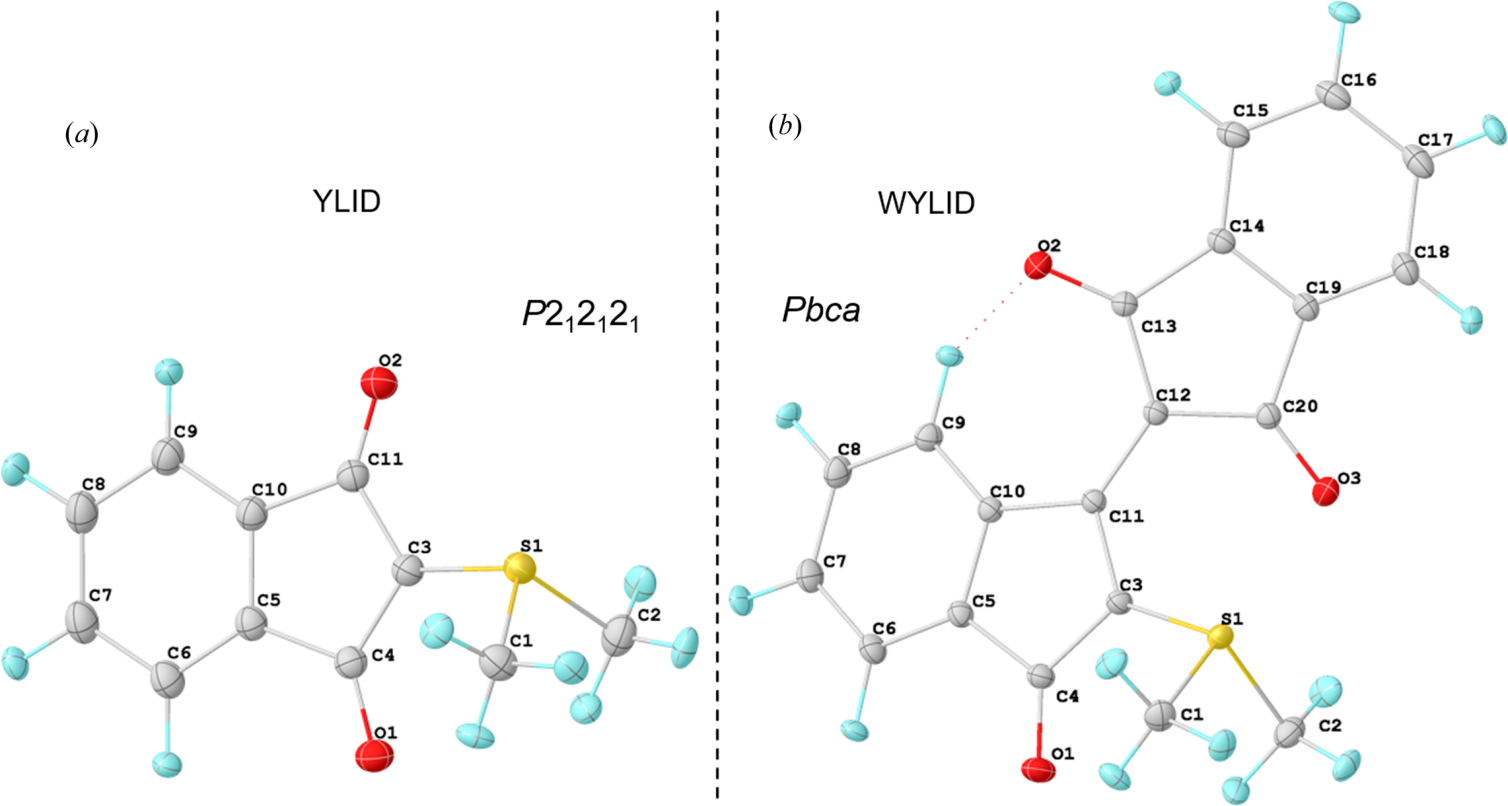
Comparison of YLID (Graw *et al.*, 2023 ▸) (110 K) and WYLID (100 K) after Hirshfeld atom refinement, with their space groups and labelling schemes. Displacement ellipsoids are drawn at the 50% probability level.

Today, most institutions engaged in structural science, chemistry, physics, biology or geology have access to such in-house X-ray diffractometers or synchrotron facilities and rely on accurate crystal structures. One of the most important prerequisites for successful and accurate structure elucidation is the calibration of the instrument in question, for which a suitable calibration crystal is required. 2-Di­methyl­sulfuran­ylidene-1,3-indane­dione, better known as YLID [Fig. 1 ▸(*a*)], has been established as a reliable, stable and well crystallizing reference. The orthorhombic form of the structure was initially used as a calibration crystal by the company Syntex in 1969 with the market release of their P-1 diffractometer. YLID’s structure was then published in 1971 by Christensen & Thom (1971 ▸). Following this, it was adopted as a calibrant by numerous different diffractometer manufacturers and has undoubtedly been the most widely measured crystal structure on single-crystal X-ray devices (Guzei *et al.*, 2008 ▸; Bal­mohammadi *et al.*, 2025*a* ▸; Bal­mohammadi *et al.*, 2025*b* ▸).

YLID possesses the advantageous properties of orthonormal cell parameters, short and long unit-cell dimensions, and a non-centrosymmetric space group (*P*2_1_2_1_2_1_). It is stable enough to be ground into crystals of spherical shape, which are well suited for a default multi-scan spherical absorption correction (Blessing, 1995 ▸; Krause *et al.*, 2015 ▸) and can be conveniently used to align the microscope of the goniometer.

Speculations of a phase transition to a second, monoclinic, modification of YLID at low temperature were disproven by Guzei *et al.* (2008 ▸), who demonstrated that both the monoclinic and orthorhombic forms are indefinitely stable between 100 and 298 K. However, the orthorhombic form was shown to be slightly favoured, as it crystallizes first and has a higher density.

**Figure 2:**
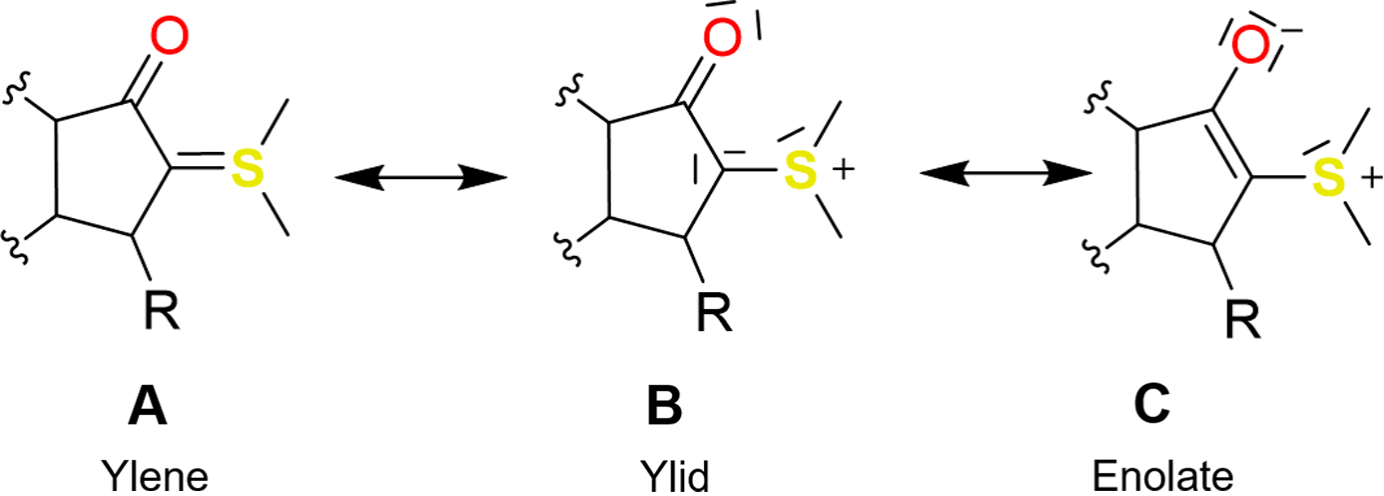
Relevant mesomeric structures of the ylid system of this study.

Even though the original purpose of YLID was the investigation of unusual bonding situations involving sulfur, the first quantum crystallographic study of YLID was only recently published by Graw *et al.* (2023 ▸). This work described YLID’s favoured resonance structure according to the quantum theory of atoms in molecules (QTAIM) (Bader, 1991 ▸) based on a multipolar modelling approach. Their YLID data were also the first instance of a successful charge-density study using In *K*α radiation. Graw *et al.* (2023 ▸) concluded that the enolate resonance form is the most accurate picture of the chemical bonding in YLID (C in Fig. 2 ▸).

Balmohammadi *et al.* (2025*a* ▸,*b* ▸) were kind enough to share their insights into the quantum crystallography of YLID with us before publication so that we could compare our results here. They investigated several structures of YLID at low and room temperature, at ambient and high pressure, and using X-rays of four distinct wavelengths (Cu *K*α, Mo *K*α and Ag *K*α, and 0.2483 Å at the SPring-8 synchrotron facility). Their study also performed a topological analysis of the total electron density according to QTAIM.

In the work by Guzei *et al.* (2008 ▸) a maroon-coloured reaction mixture is first mentioned, despite the two obtained YLID polymorphs being of yellow and orange colour, respectively. The maroon impurity could not be further identified. Upon attempting to synthesize YLID, we also observed a deep-red-coloured reaction mixture. However, the formation of red crystals alongside the yellow YLID crystals was an unexpected outcome, given that the synthesis described by Lácová & Sisková (1983 ▸) was maintained to obtain the orthorhombic and monoclinic (Guzei *et al.*, 2008 ▸) polymorphs of YLID. Single-crystal X-ray diffraction revealed the structure of these red crystals to be a condensation product between YLID and a second molecule of indandione [Fig. 1 ▸(*b*)], termed WYLID.

**Figure 3:**
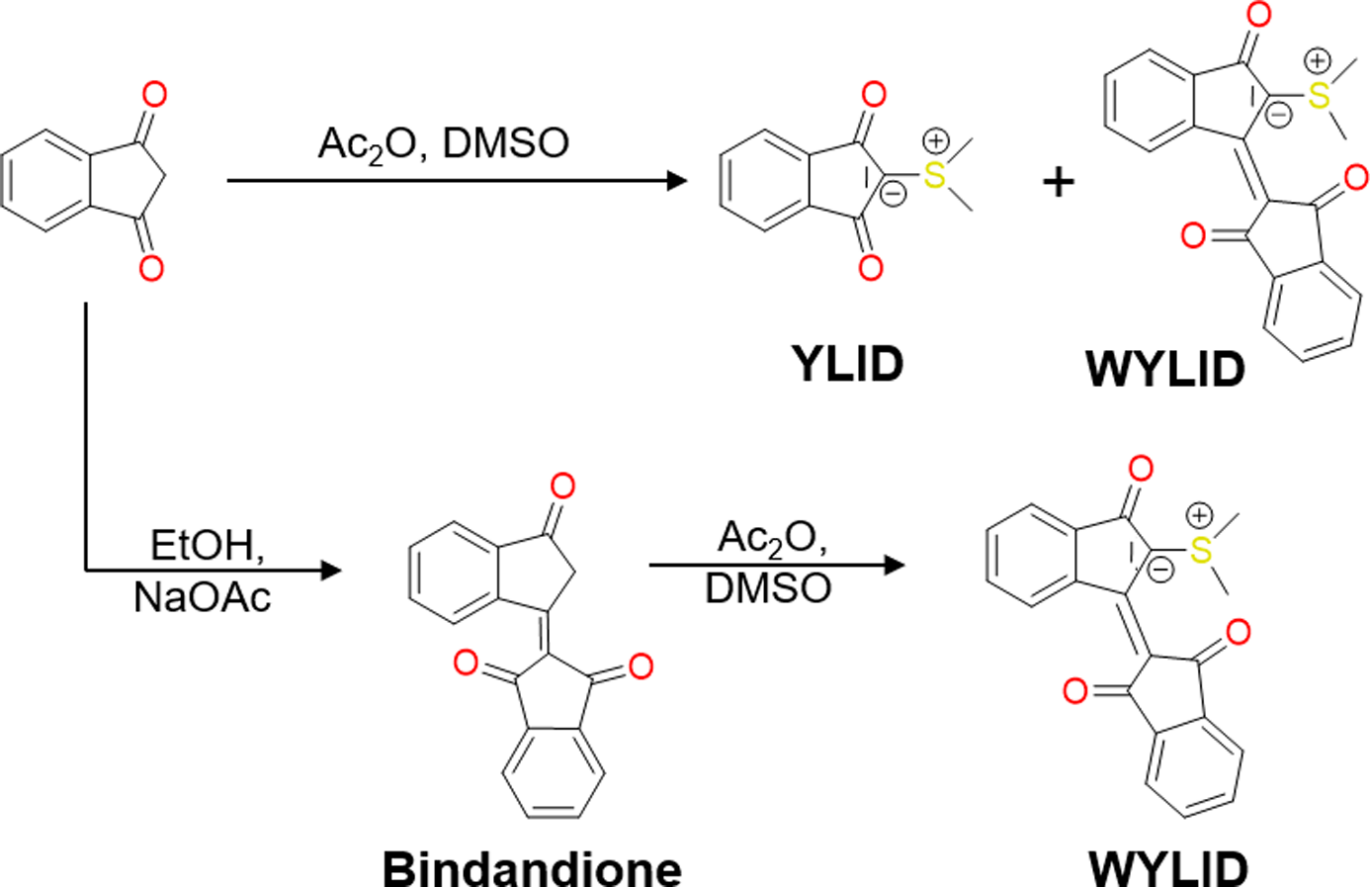
Synthesis pathways according to Lácová & Sisková (1983 ▸) and the synthesis route adapted from Bürckstümmer *et al.* (2011 ▸) for the selective synthesis of WYLID presented in this work. The reactants are acetic an­hydride (Ac_2_O), di­methyl­sulfoxide (DMSO) and ethanol (EtOH).

Both WYLID and YLID exhibit a large conjugated π system and thus many possible resonance structures. For the ylid/ylene case, the relevant structures are shown in Fig. 2 ▸. There is an ongoing debate in the quantum crystallography community about which resonance form best describes the bonding situation in YLID. While motif A has been ruled out by several observations before (Cook & Moffatt, 1968 ▸; Guzei *et al.*, 2008 ▸; Graw *et al.*, 2023 ▸), B and C are still controversial. By presenting a quantum crystallographic analysis of WYLID with a structure that exhibits one C—O functionality similar to YLID, but also two C—O bonds distant to the SMe_2_ functionality, we anticipated gaining a clearer insight into distinguishing between the carbonyl and enolate structures. For additional comparison, we also crystallized and investigated the intermediate Bindandione (Fig. 3 ▸), which gives an insight into the C—O bonding situation without the SMe_2_ group.

In this work, we describe the modified synthesis of WYLID, its characterization and its quantum crystallographic description compared with YLID. To explain and compare the C—S bonding situation in this YLID derivative, we performed a complementary bonding analysis from both experimentally driven (multipolar modelling, MM) and wavefunction-based [Hirshfeld atom refinement (HAR) and X-ray restrained wavefunction fitting (XRW)] charge-density approaches. We compare our results with those of Balmohammadi *et al.* (2025*a* ▸,*b* ▸) on YLID from their high-quality data set and with the data published by Graw *et al.* (2023 ▸). The suitability of WYLID as a calibration crystal for these quantum crystallographic descriptions has been tested using different wavelengths (Cu *K*α, Cu *K*β and Mo *K*α radiation, and synchrotron radiation at 22 keV, λ = 0.56356 Å).

## 2. Experiments and methods

### 2.1. Synthesis and characterization

The first synthetic strategy applied to reproduce WYLID was to vary the reaction conditions reported for YLID (Cook & Moffatt, 1968 ▸). This approach was based on changes in reaction time, temperature, stoichiometry (*e.g.* excess of indandione) and the order of reactants. Additionally, different methods for isolating the desired compound were tested. Despite the strong red coloration of the reaction mixture, neither of the two YLID polymorphs was selectively formed or could be isolated by the modification of this route.

To obtain WYLID exclusively, 1,3-indandione is replaced by its condensed dimer ([1,2′]bi­indenyl­idene-3,1′,3′-trione, Bindandione). The synthesis of Bindandione was carried out following the literature procedure according to Bürckstümmer *et al.* (2011 ▸) (see Section S1.2 in the supporting information). Bindandione was subsequently reacted with DMSO in acetic anhydride under conditions similar to the classic synthesis of YLID. After the reaction, the dark-red compound was recrystallized to obtain the desired WYLID selectively in moderate yields of 33%. All WYLID crystals that were used in this study were obtained via this route.

The spectral properties of WYLID in the UV–Vis region were investigated and showed a maximum absorption at λ_max_ = 508 nm with additional absorption peaks at λ = 362 nm and λ = 301 nm (Fig. S3 in the supporting information). This is consistent with the strong red colour of the crystals and solutions of WYLID. All details of the synthesis and the full characterization of WYLID can be found in the supporting information.

### 2.2. XRD measurements, data processing and modelling

The crystallographic results within this work originate from various setups using different wavelengths, different crystals, and different settings concerning the goniometer and detector used for data acquisition. Except for the Cu *K*β data set, which was recorded on an Atlas S2 CCD detector, all data sets were recorded on photon counting devices, namely a HyPix-Arc 150° (Mo *K*α and Cu *K*α) at our home laboratory XtaLAB Synergy-DW diffractometer or a Pilatus3 X 2M at the European Synchrotron on beamline BM20 (Scheinost *et al.*, 2021 ▸). The same crystal was used for the Mo *K*α and Cu *K*α data collections, but different crystals were used for the corresponding Cu *K*β and synchrotron experiments.

An earlier data set of a different WYLID crystal recorded at 20 keV at the ESRF is also compared. Due to the high flux, this measurement showed an oversaturation of the detector and therefore some strong reflections were measured with nonlinear intensity. An empirical extinction correction similar to the model presented by Ahmed *et al.* (1970 ▸) was applied to ‘correct’ this effect. Despite this misuse of extinction correction, this data set achieved a reasonable agreement between the calculated and recorded structure factors. However, we base our bond analysis on other, more reliable, data sets (Table 1 ▸) without oversaturation. Nevertheless, we consider it worth reporting on the influence of detector saturation on the results of an electron-density refinement and comparing them with the benchmark data set recorded at 22 keV without detector saturation.

An initial structure solution was obtained using *SHELXT* (Sheldrick, 2015 ▸) and the independent atom model (IAM) was refined using *olex2.refine* (Bourhis *et al.*, 2015 ▸) within *Olex2* (Dolomanov *et al.*, 2009 ▸).

Multipolar modelling according to the Hansen–Koppens formalism (Hansen & Coppens, 1978 ▸) was performed on the symmetry-merged data sets using the *MoPro* (Guillot *et al.*, 2001 ▸) software and evaluated using *VMoPro* and *MoProViwer* (Jelsch *et al.*, 2005 ▸). Anisotropic hydrogen-atom displacement parameters were adopted from a HAR (Capelli *et al.*, 2014 ▸; Hirshfeld, 1977 ▸) procedure employing *NoSpherA2* (Kleemiss *et al.*, 2021 ▸), which itself invoked the software *Orca5* (Neese *et al.*, 2020 ▸) to determine single-point molecular wavefunctions at the r2SCAN/def2-TZVP (Weigend & Ahlrichs, 2005 ▸; Furness *et al.*, 2020 ▸) level of theory. The Mo *K*α HAR-optimized X-ray geometry was used for the *TONTO* (Jayatilaka & Grimwood, 2003 ▸) software to perform an XRW fitting procedure (Jayatilaka, 1998 ▸). This XRW fitting procedure was carried out by including the weighted perturbation of the energy in the self-consistent-field (SCF) method of a Hartree–Fock calculation (Hartree, 1928 ▸; Fock, 1930 ▸; Jayatilaka, 1998 ▸). The weight was increased until no convergence could be reached anymore in the SCF procedure and the last converging step (λ = 0.07) was used as the final XRW model. The quality of each model is assessed using residual electron density, normal probability (Abrahams & Keve, 1971 ▸), DRK (Stash, 2007 ▸) and fractal dimension plots (Meindl & Henn, 2008 ▸) in the supporting information.

Table 1 ▸ shows the recorded WYLID data sets, modelling strategies and quality indicators. For further crystallographic information, the raw data and the structure data, we refer the reader to Table S1 in the supporting information.

Except for the model based on Cu *K*β radiation, all non-spherical models agreed well between the observed and calculated structure factors. As shown in the supporting information, we attribute this difference to the low raw intensity of our Cu *K*β micro-focus CCD detector setup compared with that of the rotating anode/synchrotron dual photon counting detectors used in the others. We relied on the three data sets with significantly lower *R* factors for our quantum crystallographic analysis.

## 3. Results and discussion

### 3.1. Crystal structure of WYLID

The general geometric description and Hirshfeld surface analysis using *CrystalExplorer* (Furness *et al.*, 2020 ▸) were conducted using the HAR model of the Mo *K*α data set.

WYLID crystallized in the centrosymmetric orthorhombic space group *Pbca*, different from the non-centrosymmetric *P*2_1_2_1_2_1_ space group of YLID. Table 2 ▸ shows interatomic distances in WYLID at 100 K compared with the 110 K data set for YLID obtained by Graw *et al.* (2023 ▸) and the synchrotron measurement of YLID by Balmohammadi *et al.* (2025*a* ▸).

The S1—C3 bond in WYLID is slightly shorter than the other S–methyl bonds (C2 and C3), indicating a stronger S—C interaction. Compared with the YLID model of Graw and co-workers, the S1—C3 bond is slightly longer. All three carbonyl C—O bonds in WYLID are of comparable length, with a slight exception for the C20—O3 bond which points to the C2 methyl group and exhibits a C—O⋯H2*C* interaction with a distance of 2.625 (2) Å.

A search and comparison in the Cambridge Structural Database (CSD) using the *MOGUL* tool (Bruno *et al.*, 2004 ▸) revealed that the C—O bonds in WYLID are within the estimated ranges for similar structures (Fig. S18 in the supporting information). In contrast, the S1—C3 bond in WYLID is situated between two bond distances that are more prevalent, with the shorter ones associated with YLID entries in the database (CCDC reference code MSULIN; Christensen & Thom, 1971 ▸) and the longer ones belonging to organo­metallic coordination complexes (*e.g.* CCDC refcode LUNHOU; Thorarinsdottir *et al.*, 2019 ▸). The most comparable C—S distance of 1.734 Å is found in a diester structure (CCDC refcode WOBTUE; Giovannitti *et al.*, 2014 ▸).

**Figure 4:**
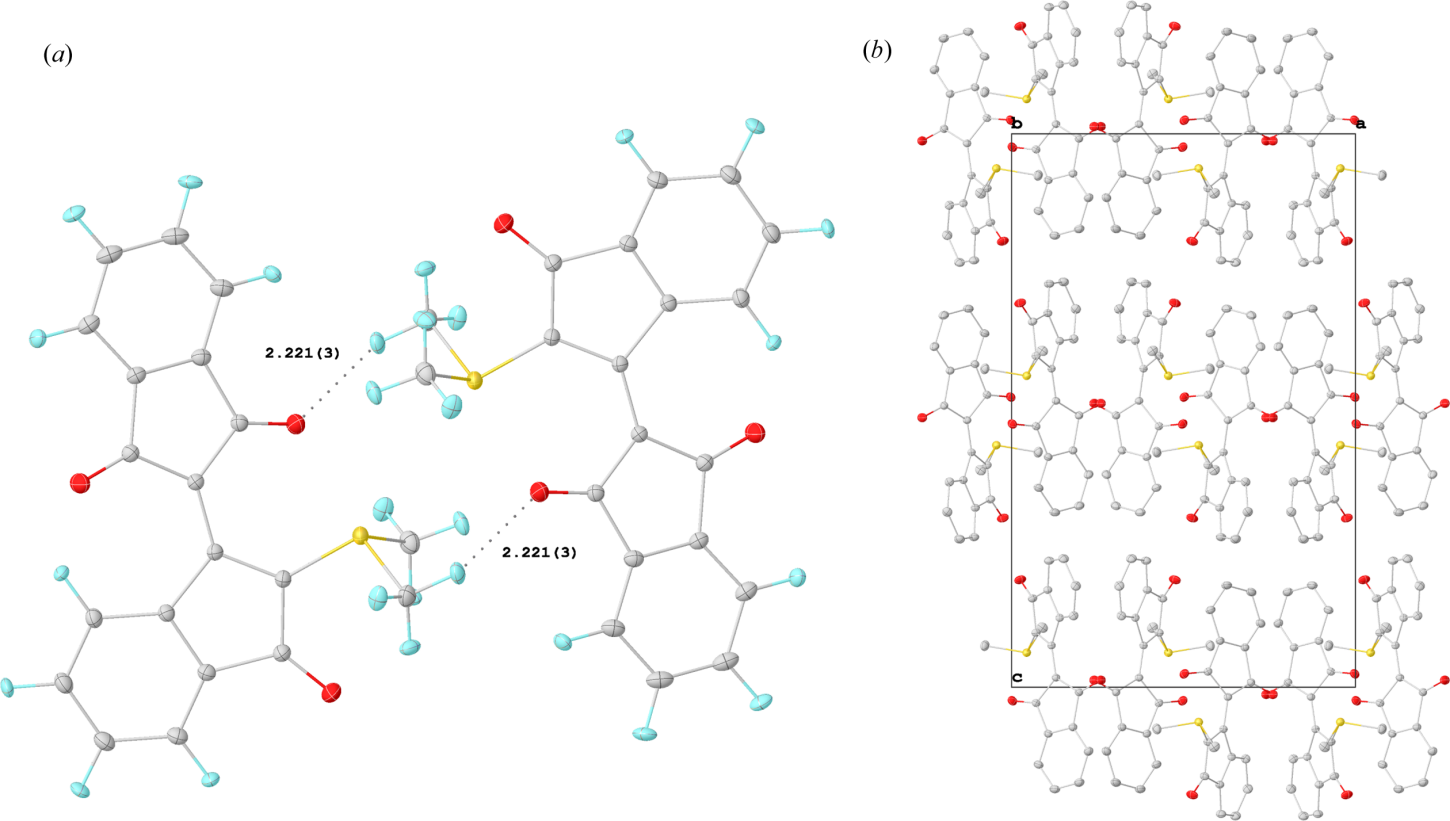
(*a*) Closest O⋯H contacts between two molecules of WYLID. (*b*) Crystal packing of the pairs in the *b* direction (H atoms omitted for clarity). Displacement ellipsoids are shown at the 50% probability level.

In the solid state, WYLID forms closely interacting pairs [Fig. 4 ▸(*a*)] dominated by O⋯H contacts between O3 and the two methyl groups. The two moieties of WYLID are positioned around a crystallographic centre of inversion, thus preventing a similar form of helical chirality as observed in YLID. These WYLID pairs align along the crystallographic *b* axis in a zigzag fashion, exhibiting weak interactions and adopting a sheet-like configuration in the crystallographic *a* direction [Fig. 4 ▸(*b*)].

Structurally, YLID and WYLID are very similar. The main difference is that one of the carbonyl oxygen atoms in YLID is replaced by another indandione moiety. This expands the aromatic system in WYLID, resulting in the colour change from yellow (YLID) to deep red (WYLID).

The sulfur atom in WYLID is slightly tilted out of the five-membered ring plane involving atoms C3, C4, C5, C10 and C11 by an angle of 8.26 (1)°, which is more than the 7° distortion in the orthorhombic YLID reported by Bal­mohammadi *et al.* (2025*a* ▸). The non-chiral monoclinic polymorph of YLID shows no distortion of the SMe_2_ fragment. This can be attributed to the steric and carbonyl electronic influence of the second indandione fragment.

Balmohammadi *et al.* (2025*a* ▸) further refined the anomalous dispersion parameters of the sulfur atoms in the Cu *K*α data sets and found small deviations from the tabulated values. We also performed an anomalous dispersion refinement (Meurer *et al.*, 2022 ▸; Balmohammadi *et al.*, 2025*a* ▸) on our Cu *K*α WYLID data set and found small deviations from the standard tables in the same range. Details are given in the supporting information.

### 3.2. Quantum crystallographic analyses

#### 3.2.1. Charge density analysis

**Figure 5:**
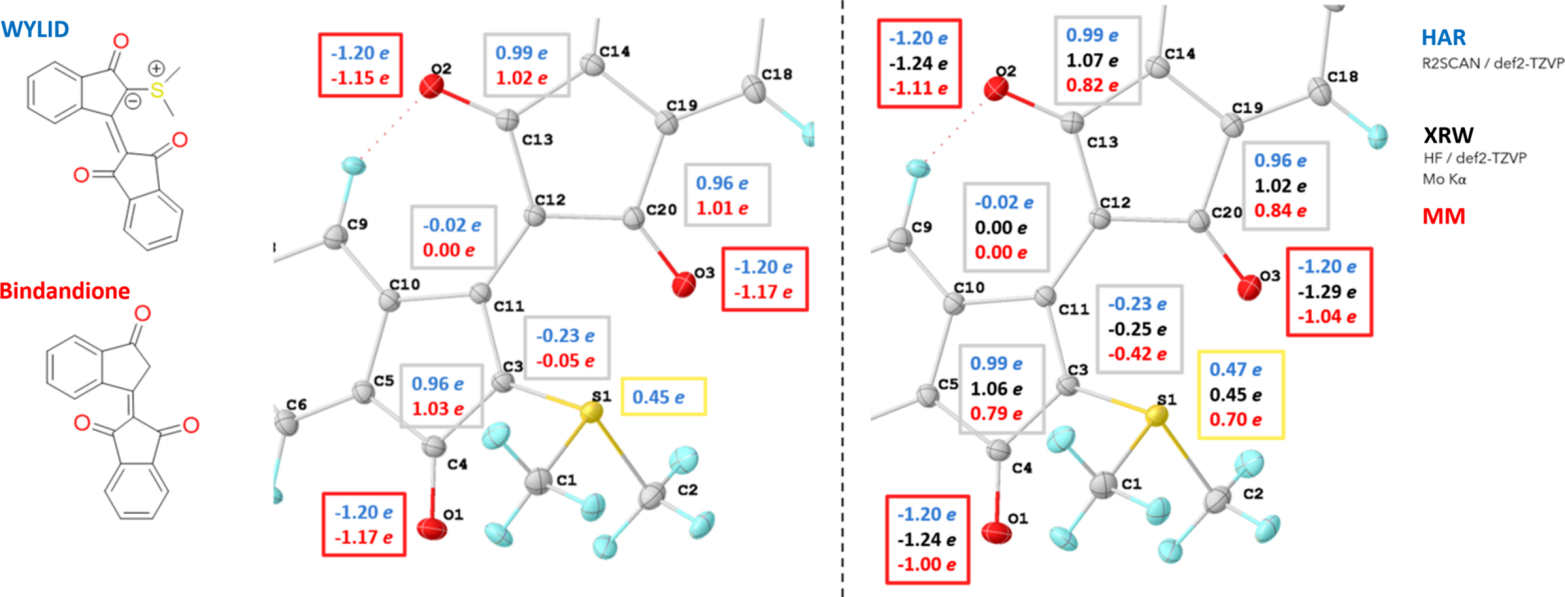
(Left) A comparison of Bader charges in WYLID (blue) and the respective charges in Bindandione (red), based on the HAR model (Mo *K*α for WYLID, Cu *K*α for Bindandione, r2SCAN/def2-TZVP). (Right) The Bader charges of WYLID from the different Mo *K*α models, namely HAR (r2SCAN/def2-TZVP), XRW (X-ray-HF/def2-TZVP) and MM.

Fig. 5 ▸ shows the atomic Bader charges according to the QTAIM (Bader, 1991 ▸) analysis of various combinations. Further atomic charges can be found in the supporting information.

In all models of WYLID, there is a significant charge separation between the sulfur atom and the attached atom C3 and between the carbonyl oxygen and their connected carbon atoms. A significant negative charge on the ylid carbon is found in the HAR and the two ‘experimental wavefunction/electron density’ approaches of XRW and MM. This is also reflected in a pure density functional theory (DFT) calculation after geometry optimization of WYLID (Section S2.4). While the multipolar model approach gives a more significant charge separation for the S1—C3 bond than the wavefunction-based methods, the opposite is true for all three carbonyl C—O pairs. The S1—C3 charge separation in the XRW model is close to that obtained by HAR (both have a difference of 0.70 e). For the C—O charges, the XRW model yields the highest charge separation of the three models (2.30 and 2.31 e). The multipolar model yields the highest S1—C3 charge separation with 1.12 e but has a lower charge separation on the carbonyls (1.79, 1.88 and 1.93 e).

**Figure 6:**
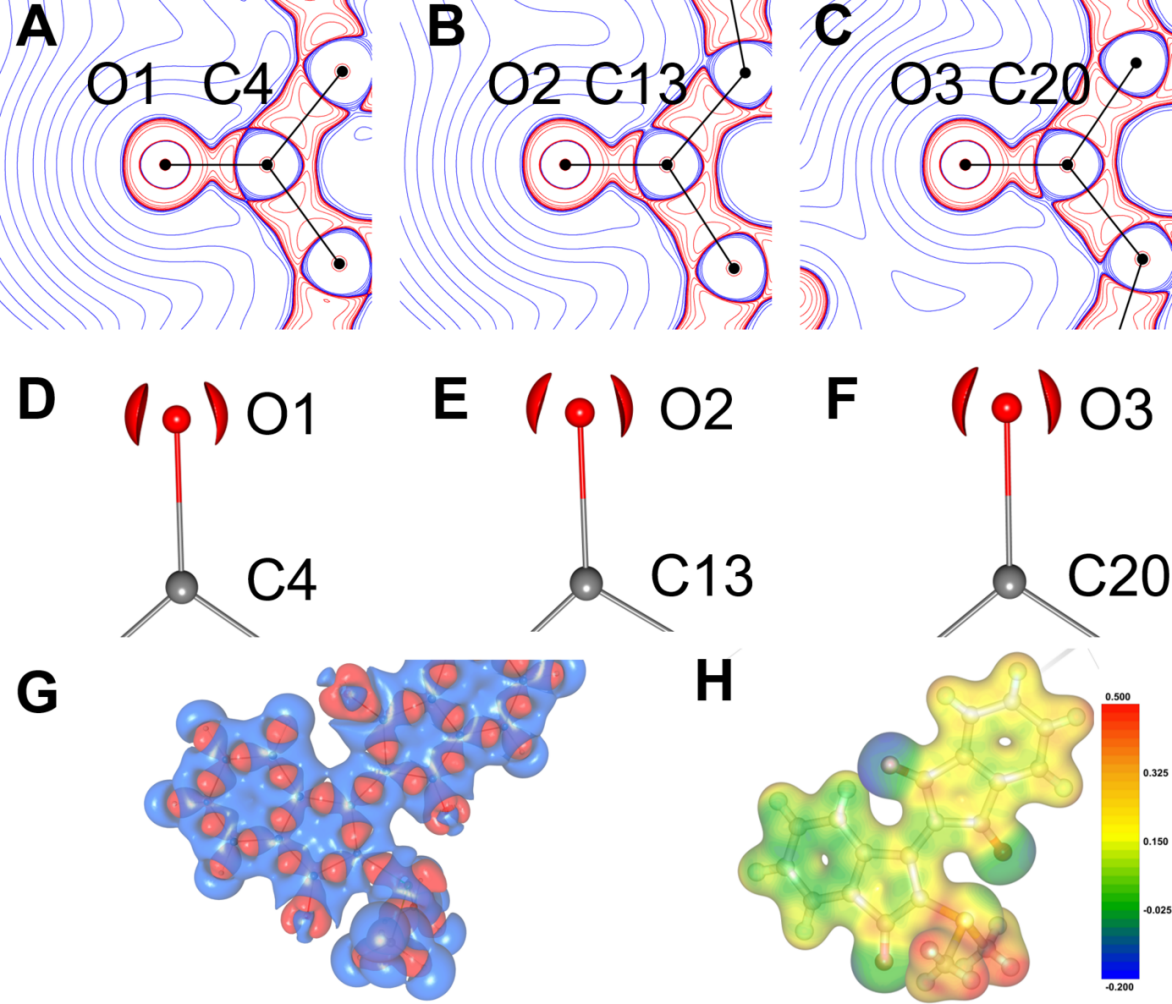
Two-dimensional Laplacians (panels A–C, −50 to 50 e Å^−5^, logarithmic iso-levels), 3D carbonyl Laplacians (panels D–F, at the −100 e Å^−5^ iso-level) and 3D Laplacians (panel G, at the 0.2 e Å^−5^ iso-surface) of the total electron density in the MM Mo *K*α model of the carbonyl groups in WYLID. Blue lines show negative Laplacian values and indicate valence-shell charge concentration. Red lines show positive Laplacian values and indicate valence-shell charge depletion. Panel H shows the electrostatic potential in e Å^−1^ mapped onto the static total electron density at the 0.2 e Å^−5^ iso-surface.

The comparison data sets between WYLID and Bindandione were recorded using different wavelengths (Mo *K*α and Cu *K*α). Despite their different maximum resolutions (0.55 and 0.80 Å, respectively), the Bader charges from each HAR model are almost identical. This indicates a similar bonding pattern, particularly for the carbonyl/enolate groups, and suggests that the presence of the SMe_2_ group does not additionally favour the enolate form (labelled C in Fig. 6). In contrast, the most significant difference between the WYLID and Bindandione charges lies in the ylid C3 atom, which is significantly negatively charged after the introduction of the SMe_2_ group.

In general, the charges we have obtained for WYLID using wavefunction-based methods agree with the charges that Balmohammadi *et al.* (2025*a* ▸) found in their analysis of YLID.

#### 3.2.2. Bonding analysis

The Laplacians of the total electron density in Fig. 6 ▸ (labelled A–F) reveal a close similarity for the three carbonyl groups in WYLID. The negative Laplacian around the carbonyl O atom shows distinct lone pairs in each case, which are in plane with the C—O bond. This indicates a preference for the carbonyl bonding scheme and an absence of the enolate form. Fig. 6 ▸ panel G shows signs of the lone pair at S1 in pseudo-tetrahedral geometry and the three S—C bonds. Table 3 ▸ shows selected bonding indices for the relevant atomic pairs in WYLID based on the Mo *K*α models. The electrostatic potential (ESP) mapped onto the static total electron density in Fig. 6 ▸ panel H shows the lowest negative ESP at the O2 carbonyl group and a higher, very similar, ESP at the O1 and O3 carbonyl groups. The highest positive ESP is found at the methyl units of the SMe_2_ group.

There is more electron density and valence-shell charge concentration at the bond critical points between the sulfur atom and the ylid carbon atom than between sulfur and the two methyl groups. This is consistent with a stronger bond between the sulfur and the ylid carbon. All bonding indices suggest a predominantly covalent single bond with a larger ionic contribution than the methyl S—C bonds exhibit. For all selected topological and bonding indicators, there is no systematic difference between the three C—O bonds in WYLID. The Wiberg bond index (WBI) suggests a bond order between 1.65 and 1.70 for each carbonyl, close to a double bond. The natural bonding orbital (NBO) analysis, however, shows that the C—O bonds have a strong ionic contribution, which is common for carbonyl systems and more so in enolate systems. The delocalization index suggests lower delocalization in the carbonyl bonds than in the S—C bonds.

**Figure 7:**
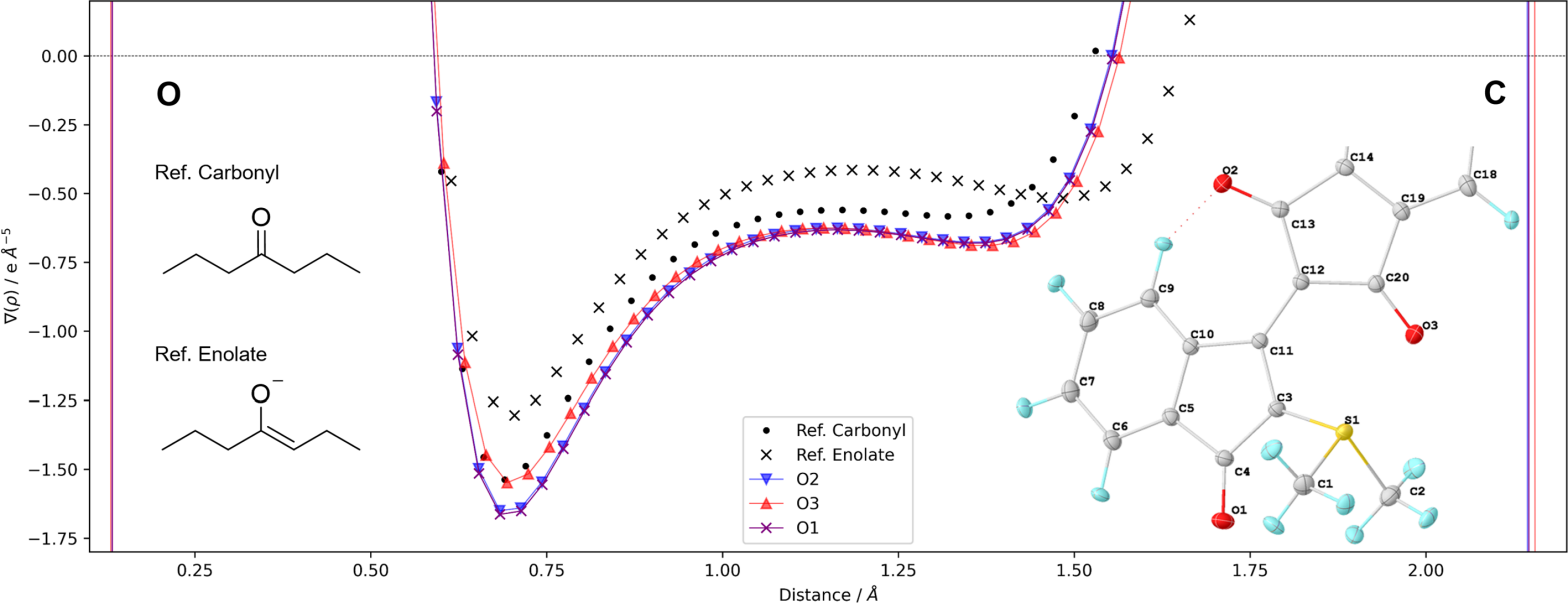
Comparison of the Laplacians of the total electron density along the topological bond path of the XRW Mo *K*α model in the three carbonyl groups in WYLID compared with the reference ketone 4-heptanone and its enolate form. More details can be found in Section S2.4 in the supporting information.

The Laplacian of the total electron density along the C—O bond path for the three carbonyls in WYLID (Fig. 7 ▸) is similar for all three carbonyl bonds. Interestingly, the largest difference was found for the O3 carbonyl bond, which interacts non-covalently with the S1—C1 anti-bond, resulting in a slightly longer S1—C1 than S1—C2 bond. This intramolecular interaction has a larger influence on the C—O bond than the proximity to the ylid bond. Compared with the reference systems of 4-heptanone and 4-hept-3-enolate, all three systems in WYLID are more similar to the Laplacian in the carbonyl than to the enolate resonance form.

In summary, the bond analysis strongly supports the ylid-type S—C bond and does not show a significant contribution from the ylene form. While the ylid bond has no significant influence on the bonding of O1 and C4, the large amount of ionic bonding together with a WBI(C—O) between 1.5 and 2 suggests that the carbonyl form has the largest contribution. A comparison of the Laplacian of the total electron density along the bond path compared with a reference carbonyl and enolate fully supports the carbonyl form. This finding is also consistent with the other bond indices, which point more towards the carbonyl structure.

#### 3.2.3. Natural resonance theory

The resonance structures in WYLID were investigated employing natural resonance theory (NRT) in *NBO 7* (Frisch *et al.*, 2013 ▸; Glendening *et al.*, 2019 ▸) using the XRW Mo *K*α model. Due to the highly delocalized π system in WYLID, a total of 175 resonance structures were found, covering 97.3% of the compound’s resonance weight, with the leading structure contributing only 2.54%. This emphasizes the difficulty of finding meaningful Lewis structures for these highly delocalized systems. Nevertheless, the summed resonance weights provide valuable information about the tendency of a Lewis structure of WYLID.

The resonance weights were analysed for atoms S1, C3, C4, O1, O2 and O3. The NRT analysis suggests a high dominance of the ylid S1—C3 bond over an ylene-type bonding. Here, the S1—C3 double bond is present in 12.0% of all resonance structures, while the ylid carbon atom C3 exhibits a lone pair in 11.1% of cases. At first glance, this seems to indicate equal ylid/ylene bonding. However, the lone pair and negative charge on C3 are most likely strongly delocalized via the π system in WYLID. In every case where the S1—C3 double bond was present, at least one other S—C bond, if not both, was cleaved.

For the carbonyl groups, the largest proportion of resonance structures prefer the carbonyl bonding scheme, being a little lower at 60.4% for O1 than for O2 and O3 (67.0% and 64.8%, respectively). This suggests that in the NRT the presence of the SMe_2_ group has a small effect on the bonding situation for O1—C4, but the difference is in the same range as between the other two carbonyl groups in WYLID. The remainder of the probabilities are mainly the enolate form but also include a third resonant structure, where only one lone pair is located at the oxygen atoms. The NBO charges and the ‘lone pairs’ as diagonals in the NBO matrix support these findings.

#### 3.2.4. Comparison with YLID

To compare our results with the classic YLID, we conducted a comparison HAR of the high-resolution data set generated by the In *K*α metal jet – one of the brightest X-ray sources apart from storage-ring facilities – and compared these results with our findings on WYLID. Specifically, we evaluated it against our best high-resolution model of WYLID coming from the BM20-CRG beamline at the ESRF. This is because the wavelength used in the synchrotron experiment (λ = 0.5634 Å, 22.0 keV) is closer to the wavelength of 0.5134 Å for the In *K*α radiation used by Graw *et al.* (2023 ▸) and has an even better agreement between the measured and calculated structure factors than the Mo *K*α HAR model.

**Figure 8:**
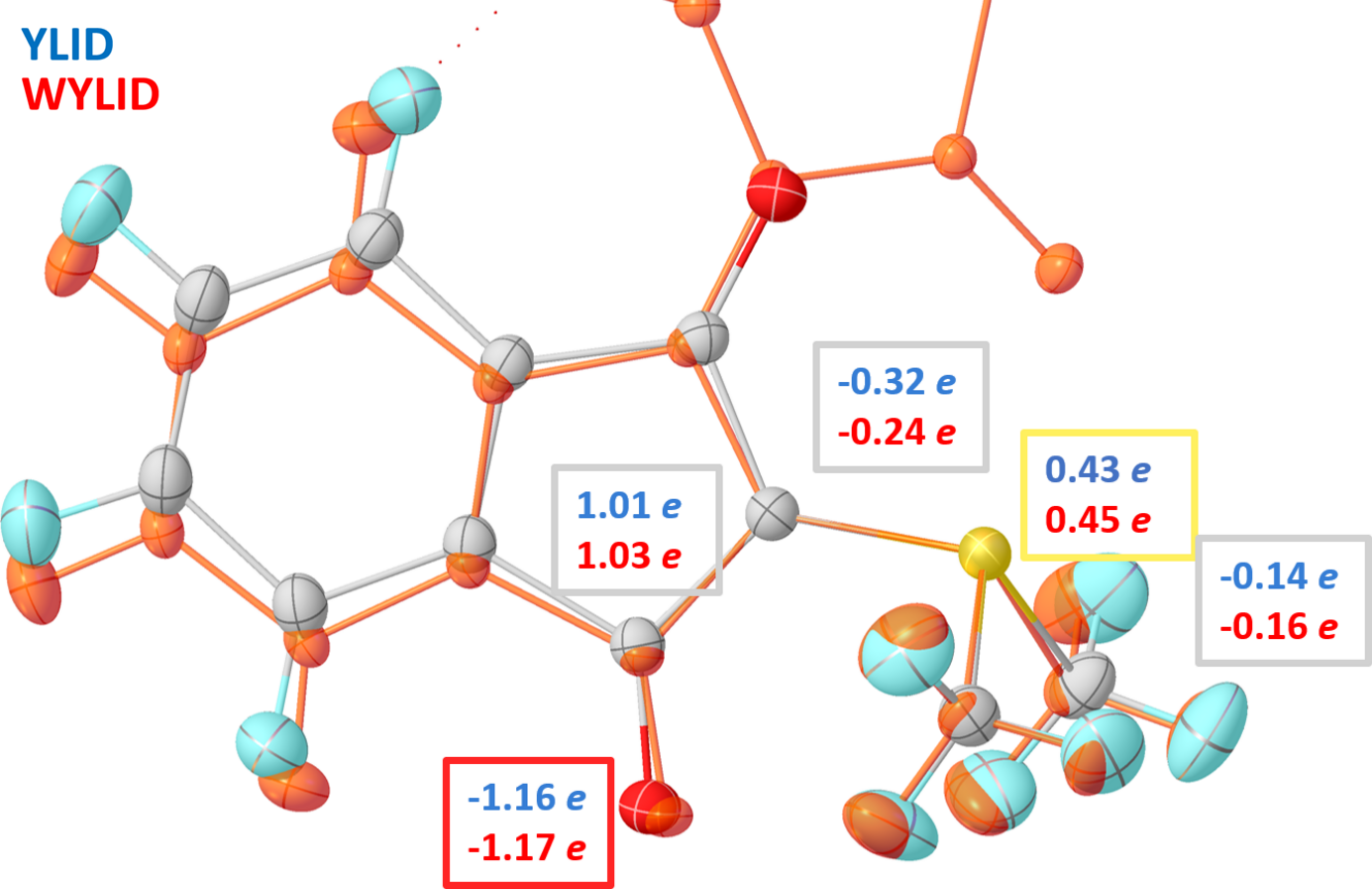
Comparison of Bader charges in electrons for YLID obtained by Graw *et al.* (2023 ▸) with a HAR using the same settings as for WYLID (transparent orange overlay).

The Bader charges in Fig. 8 ▸ for WYLID and YLID (Graw *et al.*, 2023 ▸) on the same level of theory and for similar wavelength reveal a similar bonding situation in YLID. The negative charge at the ylid carbon atom is even more pronounced in YLID than in WYLID, while the carbonyl system is almost identical. This suggests a similar ylid S—C bond as well as a similar carbonyl C—O bond in YLID.

#### 3.2.5. Comparison between XRW, HAR, HF, DFT and MM

Table 4 ▸ compares the details of the charges for the Mo *K*α based models. Generally, there was a close agreement between the wavefunction-based models. With respect to the multipolar model, the general trend is also preserved. However, the absolute charges are larger for the ylid sulfur and carbon atoms and lower in the carbonyl groups.

The NBO/NRT results are compared between the HAR, pure Hartree–Fock (HF) and XRW models concerning their natural charges, WBI and NBO. Table 5 ▸ shows the bonding parameters. In general, all parameters are in good agreement with each other and there are only small differences.

For all bonds with the exception of C13—O2, the XRW approach shows bond orders in between the pure HF approach and the HAR model. This demonstrates the general capability of XRW to include effects which are neglected in HF, such as electron correlation and polarization, and to bring additional effects into the model to the right extent. For the WBI, the XRW results in slightly weaker S—C but slightly stronger C—O bonds.

A comparison of the synchrotron data sets with the detector-saturated data set, as well as a HAR comparison using the hybrid functional ωB97X (Chai & Head-Gordon, 2008 ▸), can be found in the supporting information.

## 4. Conclusion

The synthesis and comprehensive characterization of WYLID, a byproduct of the literature synthesis of YLID, which is potentially the most extensively measured calibration mater­ial, have been presented. The combination of data from the ‘experimental charge density’ derived from multipolar modelling and the ‘experimental wavefunction’ obtained from X-ray restrained wavefunction fitting offer a profound understanding of the diverse bonding schemes observed in the WYLID compound.

A multi-wavelength complementary quantum crystallographic analysis has been employed to gain insight into the C—S and C—O bonding situation of the ylide/ylene and carbonyl/enolate systems. In the principal aspects of our analysis, the ylide structure is preferred over the ylene and the carbonyl structure is preferred over the enolate resonance form. This preference has been established by a comparison of the bonding indices for the C—S and the three C—O systems present in WYLID. In this approach, the C—O system adjacent to the ylid bond exhibits no notable distinction from the other two C—O bonds.

The analysis of WYLID was also compared with a HAR model of Bindandione, *i.e.* WYLID without the SMe_2_ group. This comparison demonstrates that the C—O bond in question exhibits no notable difference upon the introduction of the SMe_2_ group.

It can be concluded that quantum crystallography is an effective approach for elucidating the bonding situation in chemically relevant moieties, such as ylide/ylene or, arguably more importantly, carbonyl/enolate.

## 5. Related literature

For further literature related to the supporting information, see Brennan & Cowan (1992 ▸), Gasevic *et al.* (2022 ▸), Guillot (2012 ▸), Henke *et al.* (1993 ▸), Lu & Chen (2012 ▸), Rigaku Oxford Diffraction (2024 ▸) and Sasaki (1989 ▸).

## Supplementary Material

Crystal structure: contains datablock(s) Biindandione_DW_CuKa_100K, WYlid_Large6_Cub_100K_fm2, WYLID-4_22000_abs, Ylidanhydrate_MoKaDW_cut, Ylidanhydrate-CuKaDW, Ylideanhydrate-20000eV, Ylidanhydrate_MoKaDW_cut_XRW. DOI: 10.1107/S160057672500175X/pl5048sup1.cif

CIFs, fcfs and checkcifs. DOI: 10.1107/S160057672500175X/pl5048sup2.zip

Experimental details, and additional figures and tables. DOI: 10.1107/S160057672500175X/pl5048sup3.pdf

CCDC references: 2392768, 2392769, 2392770, 2392771, 2394618, 2401360, 2426878

## Figures and Tables

**Table 1:** Comparison of the different data sets recorded at 100 K and the crystallographic models used in this study

	Cu *K*α	Cu *K*β	Mo *K*α	Synchrotron
Crystal No.	1	2	1	3
Wavelength (Å)	1.54187	1.39222	0.71073	0.56356
Total reflections	204454	83609	527077	508696
Unique reflections < 2σ(*I*)	3187	4385	8605	8745
*R*_int_ (%)	2.69	7.67	2.71	4.88
Multiplicity	64.15	19.07	57.3	54.7
*I*/σ(*I*)	208.1	49.3	138.0	95.3
Resolution (Å)	0.80	0.72	0.55	0.55

IAM				
R_1_ (%)	2.82	5.12	2.86	2.63
*wR*^2^ (%)	7.36	13.24	8.91	8.49
Goodness of fit	1.066	1.042	1.022	1.020
Max, min peak (e Å^−3^)	0.320, −0.370	0.626, −0.502	0.598, −0.233	0.574, −0.380

HAR				
R_1_ (%)	0.88	3.89	1.10	1.00
*wR*^2^ (%)	1.94	10.0	1.43	1.83
Goodness of fit	1.118	1.068	1.103	1.070
Max, min peak (e Å^−3^)	−0.100, 0.065	−0.330, 0.360	0.131, −0.121	0.106, −0.117

MM				
R_1_ (%)			1.27	1.32
*wR*^2^ (%)			1.34	1.99
Goodness of fit			1.06	1.02
Max, min peak (e Å^−3^)			0.182, −0.171	0.143, −0.169

XRW				
R_1_ (%)			0.85	
*wR*^2^ (%)			0.76	
Goodness of fit			1.43	
Max, min peak (e Å^−3^)			0.132, −0.114	

**Table 2:** Selected interatomic distances (Å) in WYLID at 100 K (HAR Mo *K*α) compared with the data set presented by Graw *et al.* (2023 ▸) at 110 K and the synchrotron data set at 100 K presented by Balmohammadi *et al.* (2025*a* ▸)

Bond	WYLID, 100 K	YLID, 110 K (Graw *et al.*)	YLID, 100 K (Balmohammadi *et al.*)
S1—C3	1.7340 (1)	1.7107 (3)	1.7098 (2)
C3—C4†	1.4534 (2)	1.4363 (4), 1.4426 (3)	1.4351 (2), 1.4416 (2)
C4—O1†	1.2270 (2)	1.2349 (2), 1.2304 (3)	1.2348 (2), 1.2303 (2)
S1—C1	1.7956 (2)	1.7885 (4)	1.7889 (2)
S1—C2	1.7972 (2)	1.7975 (3)	1.7964 (2)
C11—C12	1.4014 (2)		
C13—O2	1.2261 (2)		
C20—O3	1.2316 (2)		

† For YLID, both comparable distances are given. Distances originate from the re-refinement at the same level of theory as employed in this study.

**Table 3:** Selected bonding indices of WYLID from the ‘experimental charge density’ (MM) and the ‘experimental wavefunction’ (XRW) Topological features of the bond critical points according to QTAIM, the ellipticity, the Wiberg bond index (WBI), the delocalization index (DI) and the natural bonding orbital (NBO) bond orders are shown.

	MM Mo *K*α			XRW Mo *K*α				
Atom pair	ρ_BCP_ (e Å^−3^)	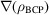 (e Å^−5^)	Ellipticity	WBI	DI	NBO total	NBO covalent	NBO ionic
S1—C3	1.42	−6.81	0.087	1.018	0.575	1.0798	0.9636	0.1162
S1—C2	1.28	−4.95	0.060	0.977	0.560	0.9209	0.8884	0.0325
S1—C1	1.26	−4.36	0.015	0.978	0.555	0.9147	0.8879	0.0268
O1—C4	2.79	−28.25	0.085	1.682	0.362	1.7568	1.1207	0.6361
O2—C13	2.78	−27.96	0.085	1.702	0.365	1.8104	1.1503	0.6601
O3—C20	2.79	−25.55	0.082	1.653	0.358	1.7787	1.1106	0.6681
C11—C12	2.11	−18.26	0.212	1.375	0.297	1.441	1.2970	0.1440

**Table 4:** Bader charges of selected atoms in WYLID from the Mo *K*α data-based models Charges given in e per atomic basin.

Atom	XRW	HAR	DFT	MM
S1	0.453	0.470	0.400	0.708
C3	−0.247	−0.238	−0.244	−0.407
C1	−0.220	−0.159	−0.178	−0.118
C2	−0.217	−0.142	−0.264	−0.138
C4	1.062	0.992	0.982	0.839
C13	1.065	0.990	1.000	0.823
C20	1.021	0.960	0.920	0.786
O1	−1.240	−1.198	−1.142	−1.002
O2	−1.237	−1.197	−1.175	−1.110
O3	−1.285	−1.185	−1.120	−1.040

**Table 5:** Results of the NBO/NRT analysis of the Mo *K*α based HAR, HF and XRW models

		WBI			NBO					
		HAR	HF	XRW	HAR total	HAR covalent	HF total	HF covalent	XRW total	XRW covalent
S1	C3	1.035	1.020	1.018	1.112	1.001	1.067	0.940	1.080	0.964
S1	C2	0.961	0.977	0.977	0.899	0.882	0.940	0.930	0.921	0.888
S1	C1	0.970	0.979	0.978	0.904	0.892	0.927	0.906	0.915	0.888
C4	O1	1.652	1.641	1.682	1.750	1.138	1.793	1.080	1.757	1.121
C13	O2	1.689	1.666	1.702	1.886	1.197	1.786	1.081	1.810	1.150
C20	O3	1.633	1.611	1.653	1.875	1.162	1.813	1.074	1.779	1.111
