# Supplementary material for: Synthesis and quantum crystallographic evaluation of WYLID: YLID’s red rival
Source: J Appl Crystallogr. 2025 Apr 4;58(Pt 3):678–87. doi: 10.1107/S160057672500175X (PMC12135977; doi:10.1107/S160057672500175X)

# checkCIF/PLATON report

Structure factors have been supplied for datablock(s) Biindandione_DW_CuKa_100K

THIS REPORT IS FOR GUIDANCE ONLY. IF USED AS PART OF A REVIEW PROCEDURE FOR PUBLICATION, IT SHOULD NOT REPLACE THE EXPERTISE OF AN EXPERIENCED CRYSTALLOGRAPHIC REFEREE.

No syntax errors found.    CIF dictionary    Interpreting this report

## Datablock: Biindandione_DW_CuKa_100K

---

```
Bond precision:   C-C = 0.0013 A          Wavelength=1.54184

Cell:              a=18.0174(1)      b=20.9221(2)      c=26.0847(2)
                   alpha=90          beta=90           gamma=90
Temperature:       100 K
```

| | Calculated | Reported |
|---|---|---|
| Volume | 9832.94(13) | 9832.94(13) |
| Space group | F d d 2 | F d d 2 |
| Hall group | F 2 -2d | F 2 -2d |
| Moiety formula | C18 H10 O3 | C18 H10 O3 |
| Sum formula | C18 H10 O3 | C18 H10 O3 |
| Mr | 274.26 | 274.28 |
| Dx,g cm-3 | 1.482 | 1.482 |
| Z | 32 | 32 |
| Mu (mm-1) | 0.826 | 0.826 |
| F000 | 4544.0 | 4560.1 |
| F000’ | 4558.62 | |
| h,k,lmax | 22,26,32 | 22,26,32 |
| Nref | 5085[ 2601] | 5026 |
| Tmin,Tmax | 0.931,0.967 | 0.895,1.000 |
| Tmin’ | 0.931 | |

```
Correction method= # Reported T Limits: Tmin=0.895 Tmax=1.000
AbsCorr = GAUSSIAN

Data completeness= 1.93/0.99        Theta(max)= 75.320

R(reflections)= 0.0161( 4962)                    wR2(reflections)=
                                                 0.0366( 5026)
S = 1.055                   Npar= 559
```

The following ALERTS were generated. Each ALERT has the format
**test-name_ALERT_alert-type_alert-level.**
Click on the hyperlinks for more details of the test.

## Alert level B

```
PLAT089_ALERT_3_B Poor Data / Parameter Ratio (Zmax < 18) ........       4.63 Note
```

## Alert level C

```
PLAT068_ALERT_1_C Reported F000 Differs from Calcd (or Missing)...     Please Check
PLAT351_ALERT_3_C Long   C-H  (X0.96,N1.08A)  C011     - H011     .       1.11 Ang.
PLAT351_ALERT_3_C Long   C-H  (X0.96,N1.08A)  C00P     - H00P     .       1.11 Ang.
PLAT351_ALERT_3_C Long   C-H  (X0.96,N1.08A)  C012     - H012     .       1.12 Ang.
PLAT411_ALERT_2_C Short Inter H...H Contact  H012     ..H015     .       2.06 Ang.
                                      1/4+x,3/4-y,-1/4+z  =      8_554 Check
```

## Alert level G

```
PLAT083_ALERT_2_G SHELXL Second Parameter in WGHT  Unusually Large       5.34 Why ?
PLAT142_ALERT_4_G s.u. on b - Axis Small or Missing .............    0.00020 Ang.
PLAT143_ALERT_4_G s.u. on c - Axis Small or Missing .............    0.00020 Ang.
PLAT720_ALERT_4_G Number of Unusual/Non-Standard Labels ..........         62 Note
              O001    O002    O003    O004    O005    O006    C007    C008
              C009    C00A    C00B    C00C    C00D    C00E    C00F    C00G
              C00H    C00I    C00J    C00K    C00L    C00M    C00N    C00O
              C00P    C00Q    C00R    C00S    C00T    C00U    C00V    C00W
              C00X    C00Y    C00Z    C010    C011    C012    C013    C014
              C015    C016    H00Y    H00X    H011    H016    H00B    H00D
              H00H    H012    H010    H014    H00S    H013    H015    H00P
              H00M    H00E    H00A    H00C    H00F    H00K
PLAT802_ALERT_4_G CIF Input Record(s) with more than 80 Characters          1 Info
PLAT881_ALERT_1_G No Datum for _diffrn_reflns_av_R_equivalents ...     Please Do !
PLAT912_ALERT_4_G Missing # of FCF Reflections Above STh/L=  0.600         14 Note
PLAT913_ALERT_3_G Missing # of Very Strong Reflections in FCF ....          1 Note
                4   4   0,
PLAT961_ALERT_5_G Dataset Contains no Negative Intensities .......     Please Check
PLAT969_ALERT_5_G The 'Henn et al.' R-Factor-gap value ...........      3.230 Note
              Predicted wR2: Based on SigI**2  1.13 or SHELX Weight  3.47
PLAT978_ALERT_2_G Number C-C Bonds with Positive Residual Density.         21 Info
PLAT979_ALERT_1_G NoSpherA2 Scattering Factors Used .............     Please Note
```

0 **ALERT level A** = Most likely a serious problem - resolve or explain
1 **ALERT level B** = A potentially serious problem, consider carefully
5 **ALERT level C** = Check. Ensure it is not caused by an omission or oversight
12 **ALERT level G** = General information/check it is not something unexpected

3 ALERT type 1 CIF construction/syntax error, inconsistent or missing data
3 ALERT type 2 Indicator that the structure model may be wrong or deficient
5 ALERT type 3 Indicator that the structure quality may be low
5 ALERT type 4 Improvement, methodology, query or suggestion
2 ALERT type 5 Informative message, check

# checkCIF publication errors

---

**Alert level A**

```
PUBL004_ALERT_1_A  The contact author's name and address are missing,
            _publ_contact_author_name and _publ_contact_author_address.
PUBL005_ALERT_1_A  _publ_contact_author_email, _publ_contact_author_fax and
            _publ_contact_author_phone are all missing.
            At least one of these should be present.
PUBL006_ALERT_1_A  _publ_requested_journal is missing
            e.g. 'Acta Crystallographica Section C'
PUBL008_ALERT_1_A  _publ_section_title is missing. Title of paper.
PUBL009_ALERT_1_A  _publ_author_name is missing. List of author(s) name(s).
PUBL010_ALERT_1_A  _publ_author_address is missing. Author(s) address(es).
PUBL012_ALERT_1_A  _publ_section_abstract is missing.
            Abstract of paper in English.
```

---

7 **ALERT level A** = Data missing that is essential or data in wrong format
0 **ALERT level G** = General alerts. Data that may be required is missing

---

**Publication of your CIF**

You should attempt to resolve as many as possible of the alerts in all categories. Often the minor alerts point to easily fixed oversights, errors and omissions in your CIF or refinement strategy, so attention to these fine details can be worthwhile. In order to resolve some of the more serious problems it may be necessary to carry out additional measurements or structure refinements. However, the nature of your study may justify the reported deviations from journal submission requirements and the more serious of these should be commented upon in the discussion or experimental section of a paper or in the "special_details" fields of the CIF. *checkCIF* was carefully designed to identify outliers and unusual parameters, but every test has its limitations and alerts that are not important in a particular case may appear. Conversely, the absence of alerts does not guarantee there are no aspects of the results needing attention. It is up to the individual to critically assess their own results and, if necessary, seek expert advice.

If level A alerts remain, which you believe to be justified deviations, and you intend to submit this CIF for publication in a journal, you should additionally insert an explanation in your CIF using the Validation Reply Form (VRF) below. This will allow your explanation to be considered as part of the review process.

```
# start Validation Reply Form
_vrf_PUBL004_GLOBAL
;
PROBLEM: The contact author's name and address are missing,
RESPONSE: ...
;
_vrf_PUBL005_GLOBAL
;
PROBLEM: _publ_contact_author_email, _publ_contact_author_fax and
RESPONSE: ...
```

```
;
_vrf_PUBL006_GLOBAL
;
PROBLEM: _publ_requested_journal is missing
RESPONSE: ...
;
_vrf_PUBL008_GLOBAL
;
PROBLEM: _publ_section_title is missing. Title of paper.
RESPONSE: ...
;
_vrf_PUBL009_GLOBAL
;
PROBLEM: _publ_author_name is missing. List of author(s) name(s).
RESPONSE: ...
;
_vrf_PUBL010_GLOBAL
;
PROBLEM: _publ_author_address is missing. Author(s) address(es).
RESPONSE: ...
;
_vrf_PUBL012_GLOBAL
;
PROBLEM: _publ_section_abstract is missing.
RESPONSE: ...
;
# end Validation Reply Form
```

If you wish to submit your CIF for publication in Acta Crystallographica Section C or E, you should upload your CIF via the web. If you wish to submit your CIF for publication in IUCrData you should upload your CIF via the web. If your CIF is to form part of a submission to another IUCr journal, you will be asked, either during electronic submission or by the Co-editor handling your paper, to upload your CIF via our web site.

---

**PLATON version of 22/08/2024; check.def file version of 21/08/2024**

**Datablock Biindandione_DW_CuKa_100K** - ellipsoid plot

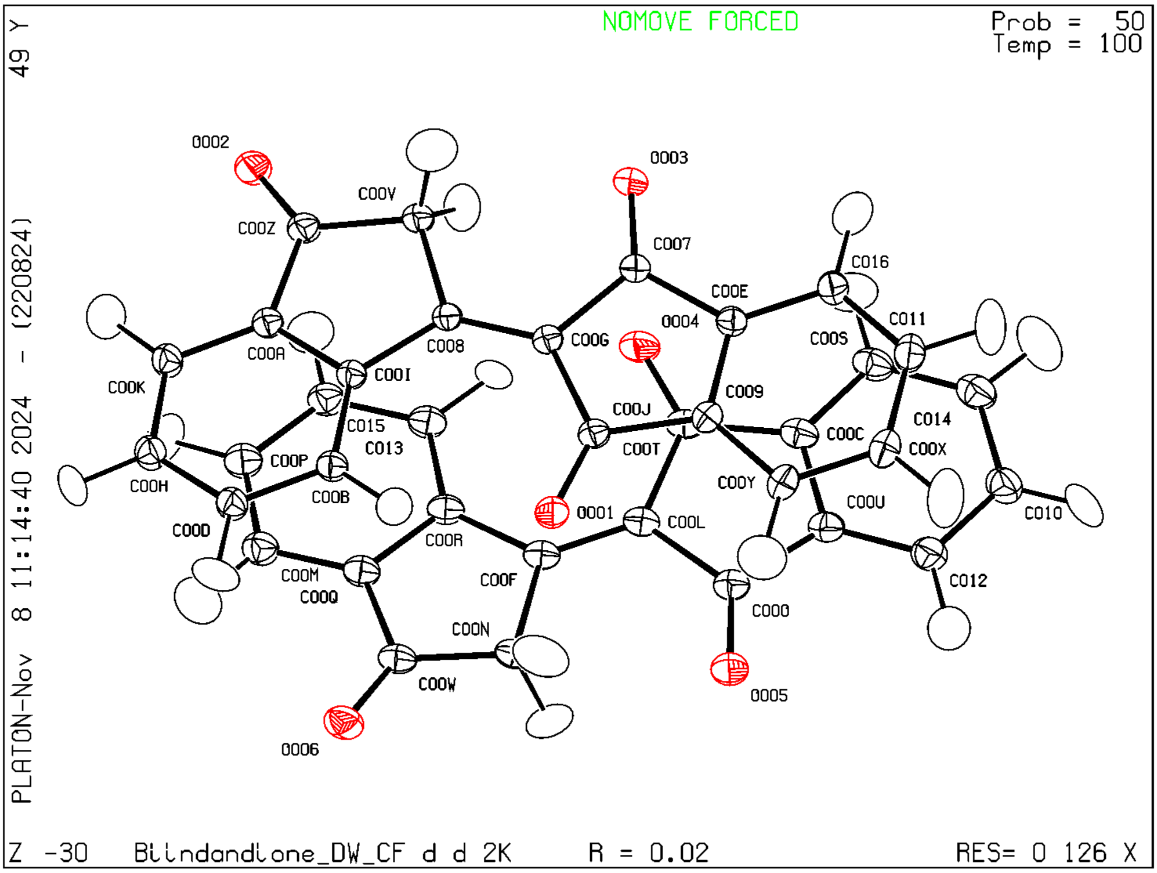

Supplement: Supplementary file 2 [file j-58-00678-sup2.zip › bindandione_checkcif.pdf]
