# Supplementary material for: Synthesis and quantum crystallographic evaluation of WYLID: YLID’s red rival
Source: J Appl Crystallogr. 2025 Apr 4;58(Pt 3):678–87. doi: 10.1107/S160057672500175X (PMC12135977; doi:10.1107/S160057672500175X)

checkCIF/PLATON report

Structure factors have been supplied for datablock(s) I

THIS REPORT IS FOR GUIDANCE ONLY. IF USED AS PART OF A REVIEW PROCEDURE FOR PUBLICATION, IT SHOULD NOT REPLACE THE EXPERTISE OF AN EXPERIENCED CRYSTALLOGRAPHIC REFEREE.

No syntax errors found. CIF dictionary Interpreting this report

Datablock: I

Bond precision:	C-C = 0.0003 A	Wavelength=0.56356	
Cell:	a=14.4906(1)	b=9.15690	c=23.32900
	alpha=90	beta=90	gamma=90
Temperature:	100 K		

	Calculated	Reported
Volume	3095.50(2)	3095.50(2)
Space group	P b c a	P b c a
Hall group	-P 2ac 2ab	-P 2ac 2ab
Moiety formula	C20 H14 O3 S	C20 H14 O3 S
Sum formula	C20 H14 O3 S	C20 H14 O3 S
Mr	334.37	334.38
Dx,g cm-3	1.435	1.435
Z	8	8
Mu (mm-1)	0.125	0.122
F000	1392.0	1392.0
F000'	1392.88	
h,k,lmax	26,16,42	25,16,42
Nref	9843	9474
Tmin,Tmax	0.988,0.988	0.771,1.000
Tmin'	0.988	

Correction method= # Reported T Limits: Tmin=0.771 Tmax=1.000
AbsCorr = MULTI-SCAN

Data completeness= 0.963 Theta(max)= 30.930

R(reflections)= 0.0111(8419) wR2(reflections)= 0.0199(9471)

S = 1.016 Npar= 787

The following ALERTS were generated. Each ALERT has the format
test-name_ALERT_alert-type_alert-level.
Click on the hyperlinks for more details of the test.

Alert level C

```
PLAT001_ALERT_1_C No _shelx_res_file DataName Found in SHELXL CIF      Please Do !
PLAT105_ALERT_1_C CIF and RES reported wavelengths inconsistent ..          ? Check
PLAT142_ALERT_4_C s.u. on b - Axis Small or Missing .............    0.00000 Ang.
PLAT143_ALERT_4_C s.u. on c - Axis Small or Missing .............    0.00000 Ang.
PLAT218_ALERT_3_C Constrained U(i,j) Components(s) for H9                   6 Check
PLAT218_ALERT_3_C Constrained U(i,j) Components(s) for H16                  6 Check
PLAT218_ALERT_3_C Constrained U(i,j) Components(s) for H18                  6 Check
PLAT218_ALERT_3_C Constrained U(i,j) Components(s) for H15                  6 Check
PLAT218_ALERT_3_C Constrained U(i,j) Components(s) for H2A                  6 Check
PLAT218_ALERT_3_C Constrained U(i,j) Components(s) for H8                   6 Check
PLAT218_ALERT_3_C Constrained U(i,j) Components(s) for H1A                  6 Check
PLAT218_ALERT_3_C Constrained U(i,j) Components(s) for H6                   6 Check
PLAT218_ALERT_3_C Constrained U(i,j) Components(s) for H2B                  6 Check
PLAT218_ALERT_3_C Constrained U(i,j) Components(s) for H1B                  6 Check
PLAT218_ALERT_3_C Constrained U(i,j) Components(s) for H17                  6 Check
PLAT218_ALERT_3_C Constrained U(i,j) Components(s) for H7                   6 Check
PLAT218_ALERT_3_C Constrained U(i,j) Components(s) for H1C                  6 Check
PLAT218_ALERT_3_C Constrained U(i,j) Components(s) for H2C                  6 Check
PLAT911_ALERT_3_C Missing FCF Refl Between Thmin & STh/L=    0.600         17 Report
               11  0  4,  14  3  4,   3  7  5,   5  8  5,   2  9  6,   5  8  8,
                7  1  8,   1 10  9,  11  7 11,  13  1 14,  13  4 14,   2  7 19,
               11  1 19,  10  4 20,   3  6 21,   2  3 25,   3  1 26,
```

Alert level G

```
ABSMU01_ALERT_1_G  Calculation of _exptl_absorpt_correction_mu
                not performed for this radiation type.
PLAT002_ALERT_2_G Number of Distance or Angle Restraints on AtSite         14 Note
PLAT860_ALERT_3_G Number of Least-Squares Restraints ............         317 Note
PLAT881_ALERT_1_G No Datum for _diffrn_reflns_av_R_equivalents ...     Please Do !
PLAT910_ALERT_3_G Missing # of FCF Reflection(s) Below Theta(Min).          1 Note
                0  0  2,
PLAT912_ALERT_4_G Missing # of FCF Reflections Above STh/L=  0.600        333 Note
PLAT929_ALERT_5_G No Weight Pars,Obs and Calc R1,wR2,S not Checked          ! Info
PLAT961_ALERT_5_G Dataset Contains no Negative Intensities .......     Please Check
PLAT969_ALERT_5_G The 'Henn et al.' R-Factor-gap value ...........      1.034 Note
              Predicted wR2: Based on SigI**2  1.92 or SHELX Weight  1.92
PLAT978_ALERT_2_G Number C-C Bonds with Positive Residual Density.         19 Info
PLAT984_ALERT_1_G The  C-f'=    0.0033 Deviates from the B&C-Value     0.0014 Check
PLAT984_ALERT_1_G The  O-f'=    0.0106 Deviates from the B&C-Value     0.0059 Check
PLAT984_ALERT_1_G The  S-f'=    0.1246 Deviates from the B&C-Value     0.0838 Check
PLAT985_ALERT_1_G The  O-f"=    0.0060 Deviates from the B&C-Value     0.0036 Check
PLAT985_ALERT_1_G The  S-f"=    0.1234 Deviates from the B&C-Value     0.0777 Check
```

0 **ALERT level A** = Most likely a serious problem - resolve or explain
0 **ALERT level B** = A potentially serious problem, consider carefully
19 **ALERT level C** = Check. Ensure it is not caused by an omission or oversight
15 **ALERT level G** = General information/check it is not something unexpected

```
  9 ALERT type 1 CIF construction/syntax error, inconsistent or missing data
  2 ALERT type 2 Indicator that the structure model may be wrong or deficient
 17 ALERT type 3 Indicator that the structure quality may be low
  3 ALERT type 4 Improvement, methodology, query or suggestion
  3 ALERT type 5 Informative message, check
```

It is advisable to attempt to resolve as many as possible of the alerts in all categories. Often the minor alerts point to easily fixed oversights, errors and omissions in your CIF or refinement strategy, so attention to these fine details can be worthwhile. In order to resolve some of the more serious problems it may be necessary to carry out additional measurements or structure refinements. However, the purpose of your study may justify the reported deviations and the more serious of these should normally be commented upon in the discussion or experimental section of a paper or in the "special_details" fields of the CIF. checkCIF was carefully designed to identify outliers and unusual parameters, but every test has its limitations and alerts that are not important in a particular case may appear. Conversely, the absence of alerts does not guarantee there are no aspects of the results needing attention. It is up to the individual to critically assess their own results and, if necessary, seek expert advice.

Publication of your CIF in IUCr journals

A basic structural check has been run on your CIF. These basic checks will be run on all CIFs submitted for publication in IUCr journals (*Acta Crystallographica*, *Journal of Applied Crystallography*, *Journal of Synchrotron Radiation*); however, if you intend to submit to *Acta Crystallographica Section C* or *E* or *IUCrData*, you should make sure that full publication checks are run on the final version of your CIF prior to submission.

Publication of your CIF in other journals

Please refer to the *Notes for Authors* of the relevant journal for any special instructions relating to CIF submission.

PLATON version of 22/08/2024; check.def file version of 21/08/2024

Datablock I - ellipsoid plot

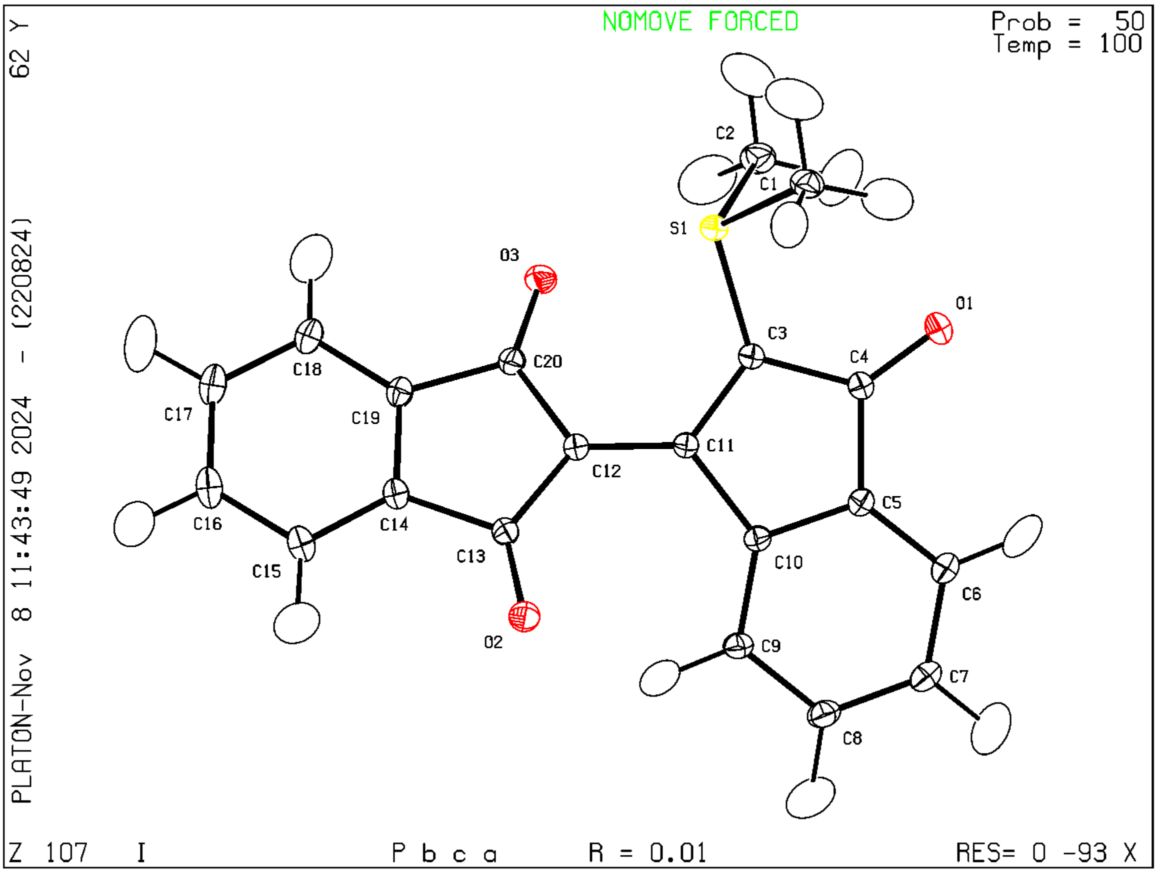

Supplement: Supplementary file 2 [file j-58-00678-sup2.zip › WYLID_22keV_MM_checkcif.pdf]
