# Supplementary material for: Synthesis and quantum crystallographic evaluation of WYLID: YLID’s red rival
Source: J Appl Crystallogr. 2025 Apr 4;58(Pt 3):678–87. doi: 10.1107/S160057672500175X (PMC12135977; doi:10.1107/S160057672500175X)

# checkCIF/PLATON report

Structure factors have been supplied for datablock(s) WYlid_Large6_Cub_100K_fm2

THIS REPORT IS FOR GUIDANCE ONLY. IF USED AS PART OF A REVIEW PROCEDURE FOR PUBLICATION, IT SHOULD NOT REPLACE THE EXPERTISE OF AN EXPERIENCED CRYSTALLOGRAPHIC REFEREE.

No syntax errors found.     CIF dictionary     Interpreting this report

## Datablock: WYlid_Large6_Cub_100K_fm2

---

```
Bond precision:   C-C = 0.0016 A          Wavelength=1.39222

Cell:              a=14.4765(2)      b=9.1539(1)      c=23.3338(4)
                   alpha=90          beta=90          gamma=90
Temperature:       100 K

                 Calculated                  Reported
Volume           3092.11(8)                  3092.11(8)
Space group      P b c a                     P b c a
Hall group       -P 2ac 2ab                  -P 2ac 2ab
Moiety formula   C20 H14 O3 S                C20 H14 O3 S
Sum formula      C20 H14 O3 S                C20 H14 O3 S
Mr               334.37                      334.40
Dx,g cm-3        1.436                       1.437
Z                8                           8
Mu (mm-1)        1.447                       1.447
F000             1392.0                      1397.8
F000'            1397.64
h,k,lmax         20,12,32                    20,12,32
Nref             4398                        4385
Tmin,Tmax        0.763,0.849                 0.490,1.000
Tmin'            0.675

Correction method= # Reported T Limits: Tmin=0.490 Tmax=1.000
AbsCorr = GAUSSIAN

Data completeness= 0.997          Theta(max)= 76.180

                                                      wR2(reflections)=
R(reflections)= 0.0389( 4228)                         0.1000( 4385)

S = 1.068                 Npar= 343
```

The following ALERTS were generated. Each ALERT has the format
**test-name_ALERT_alert-type_alert-level.**
Click on the hyperlinks for more details of the test.

### Alert level C

```
PLAT906_ALERT_3_C Large K Value in the Analysis of Variance ......      3.367 Check
```

### Alert level G

```
ABSMU01_ALERT_1_G  Calculation of _exptl_absorpt_correction_mu
                not performed for this radiation type.
PLAT003_ALERT_2_G Number of Uiso or U(i,j) Restrained non-H Atoms         24 Report
PLAT068_ALERT_1_G Reported F000 Differs from Calcd (or Missing)...     Please Check
PLAT092_ALERT_4_G Check: Wavelength Given is not Cu,Ga,Mo,Ag,In Ka    1.39222 Ang.
PLAT187_ALERT_4_G The CIF-Embedded .res File Contains RIGU Records         2 Report
PLAT190_ALERT_3_G A Non-default RIGU Restraint Value for First Par     0.0100 Report
PLAT190_ALERT_3_G A Non-default RIGU Restraint Value for SecondPar     0.0200 Report
PLAT190_ALERT_3_G A Non-default RIGU Restraint Value for First Par     0.0100 Report
PLAT190_ALERT_3_G A Non-default RIGU Restraint Value for SecondPar     0.0100 Report
PLAT802_ALERT_4_G CIF Input Record(s) with more than 80 Characters         1 Info
PLAT860_ALERT_3_G Number of Least-Squares Restraints ............        534 Note
PLAT881_ALERT_1_G No Datum for _diffrn_reflns_av_R_equivalents ...     Please Do !
PLAT883_ALERT_1_G No Info/Value for _atom_sites_solution_primary .     Please Do !
PLAT910_ALERT_3_G Missing # of FCF Reflection(s) Below Theta(Min).         1 Note
                0  0  2,
PLAT912_ALERT_4_G Missing # of FCF Reflections Above STh/L=  0.600        12 Note
PLAT969_ALERT_5_G The 'Henn et al.' R-Factor-gap value ..........      4.153 Note
              Predicted wR2: Based on SigI**2  2.41 or SHELX Weight  9.36
PLAT978_ALERT_2_G Number C-C Bonds with Positive Residual Density.        18 Info
PLAT979_ALERT_1_G NoSpherA2 Scattering Factors Used .............     Please Note
```

0 **ALERT level A** = Most likely a serious problem - resolve or explain
0 **ALERT level B** = A potentially serious problem, consider carefully
1 **ALERT level C** = Check. Ensure it is not caused by an omission or oversight
18 **ALERT level G** = General information/check it is not something unexpected

5 ALERT type 1 CIF construction/syntax error, inconsistent or missing data
2 ALERT type 2 Indicator that the structure model may be wrong or deficient
7 ALERT type 3 Indicator that the structure quality may be low
4 ALERT type 4 Improvement, methodology, query or suggestion
1 ALERT type 5 Informative message, check

It is advisable to attempt to resolve as many as possible of the alerts in all categories. Often the minor alerts point to easily fixed oversights, errors and omissions in your CIF or refinement strategy, so attention to these fine details can be worthwhile. In order to resolve some of the more serious problems it may be necessary to carry out additional measurements or structure refinements. However, the purpose of your study may justify the reported deviations and the more serious of these should normally be commented upon in the discussion or experimental section of a paper or in the "special_details" fields of the CIF. checkCIF was carefully designed to identify outliers and unusual parameters, but every test has its limitations and alerts that are not important in a particular case may appear. Conversely, the absence of alerts does not guarantee there are no aspects of the results needing attention. It is up to the individual to critically assess their own results and, if necessary, seek expert advice.

**Publication of your CIF in IUCr journals**

A basic structural check has been run on your CIF. These basic checks will be run on all CIFs submitted for publication in IUCr journals (*Acta Crystallographica*, *Journal of Applied Crystallography*, *Journal of Synchrotron Radiation*); however, if you intend to submit to *Acta Crystallographica Section C* or *E* or *IUCrData*, you should make sure that full publication checks are run on the final version of your CIF prior to submission.

**Publication of your CIF in other journals**

Please refer to the *Notes for Authors* of the relevant journal for any special instructions relating to CIF submission.

---

**PLATON version of 22/08/2024; check.def file version of 21/08/2024**

**Datablock WYlid_Large6_Cub_100K_fm2** - ellipsoid plot

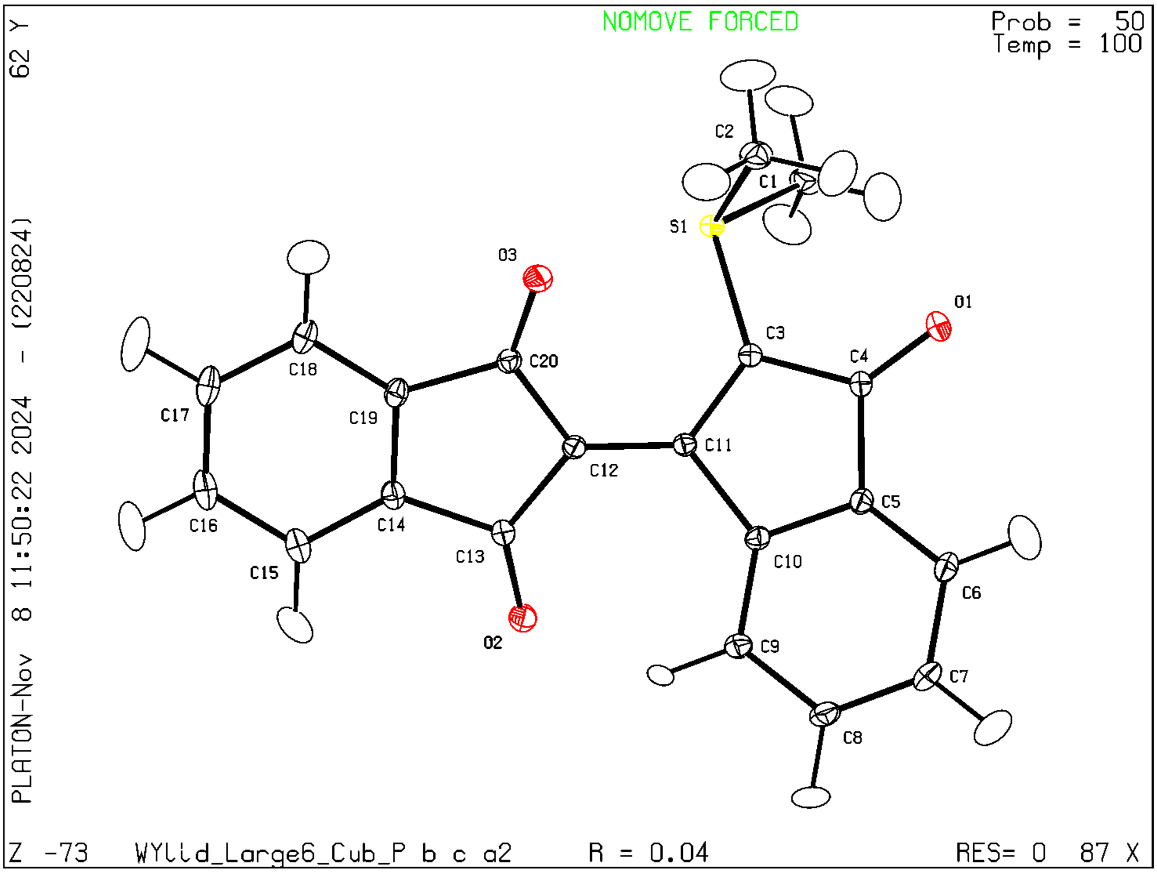

Supplement: Supplementary file 2 [file j-58-00678-sup2.zip › WYLID_Large6_Cub_100K_fm2_checkcif.pdf]
