# Supplementary material for: Synthesis and quantum crystallographic evaluation of WYLID: YLID’s red rival
Source: J Appl Crystallogr. 2025 Apr 4;58(Pt 3):678–87. doi: 10.1107/S160057672500175X (PMC12135977; doi:10.1107/S160057672500175X)

checkCIF/PLATON report

Structure factors have been supplied for datablock(s) Ylidanhydrate_MoKaDW_cut

THIS REPORT IS FOR GUIDANCE ONLY. IF USED AS PART OF A REVIEW PROCEDURE FOR PUBLICATION, IT SHOULD NOT REPLACE THE EXPERTISE OF AN EXPERIENCED CRYSTALLOGRAPHIC REFEREE.

No syntax errors found. CIF dictionary Interpreting this report

Datablock: Ylidanhydrate_MoKaDW_cut

```
Bond precision:   C-C = 0.0002 A           Wavelength=0.71073

Cell:            a=14.49062(7)     b=9.15691(4)     c=23.32900(11)
                 alpha=90          beta=90          gamma=90
Temperature:     100 K
```

	Calculated	Reported
Volume	3095.51(2)	3095.51(2)
Space group	P b c a	P b c a
Hall group	-P 2ac 2ab	-P 2ac 2ab
Moiety formula	C20 H14 O3 S	C20 H14 O3 S
Sum formula	C20 H14 O3 S	C20 H14 O3 S
Mr	334.37	334.40
Dx,g cm-3	1.435	1.435
Z	8	8
Mu (mm-1)	0.224	0.224
F000	1392.0	1393.8
F000’	1393.63	
h,k,lmax	26,16,42	26,16,42
Nref	9752	9749
Tmin,Tmax	0.973,0.987	0.707,1.000
Tmin’	0.958	

```
Correction method= # Reported T Limits: Tmin=0.707 Tmax=1.000
AbsCorr = GAUSSIAN

Data completeness= 1.000            Theta(max)= 40.250

R(reflections)= 0.0111( 8605)                 wR2(reflections)= 0.0164( 9749)

S = 1.153                   Npar= 418
```

The following ALERTS were generated. Each ALERT has the format
test-name_ALERT_alert-type_alert-level.
Click on the hyperlinks for more details of the test.

Alert level B

```
PLAT230_ALERT_2_B Hirshfeld Test Diff for    S1       --C2       .       13.4 s.u.
```

Alert level G

```
PLAT003_ALERT_2_G Number of Uiso or U(i,j) Restrained non-H Atoms         24 Report
PLAT068_ALERT_1_G Reported F000 Differs from Calcd (or Missing)...     Please Check
PLAT142_ALERT_4_G s.u. on b - Axis Small or Missing .............    0.00004 Ang.
PLAT143_ALERT_4_G s.u. on c - Axis Small or Missing .............    0.00011 Ang.
PLAT187_ALERT_4_G The CIF-Embedded .res File Contains RIGU Records         2 Report
PLAT190_ALERT_3_G A Non-default RIGU Restraint Value for First Par     0.2000 Report
PLAT190_ALERT_3_G A Non-default RIGU Restraint Value for SecondPar     0.2000 Report
PLAT190_ALERT_3_G A Non-default RIGU Restraint Value for First Par     0.0100 Report
PLAT190_ALERT_3_G A Non-default RIGU Restraint Value for SecondPar     0.0100 Report
PLAT802_ALERT_4_G CIF Input Record(s) with more than 80 Characters         1 Info
PLAT860_ALERT_3_G Number of Least-Squares Restraints ............        207 Note
PLAT872_ALERT_4_G ALERTS Related to Anharmonic Refine   Suppressed         ! Info
PLAT910_ALERT_3_G Missing # of FCF Reflection(s) Below Theta(Min).         1 Note
                0  0  2,
PLAT933_ALERT_2_G Number of HKL-OMIT Records in Embedded .res File         1 Note
                1  4  2,
PLAT960_ALERT_3_G Number of Intensities with I  <   - 2*sig(I) ...         4 Check
PLAT969_ALERT_5_G The 'Henn et al.' R-Factor-gap value ..........      2.845 Note
              Predicted wR2: Based on SigI**2  0.58 or SHELX Weight  1.43
PLAT978_ALERT_2_G Number C-C Bonds with Positive Residual Density.        20 Info
PLAT979_ALERT_1_G NoSpherA2 Scattering Factors Used .............     Please Note
```

0 **ALERT level A** = Most likely a serious problem - resolve or explain
1 **ALERT level B** = A potentially serious problem, consider carefully
0 **ALERT level C** = Check. Ensure it is not caused by an omission or oversight
18 **ALERT level G** = General information/check it is not something unexpected

2 ALERT type 1 CIF construction/syntax error, inconsistent or missing data
4 ALERT type 2 Indicator that the structure model may be wrong or deficient
7 ALERT type 3 Indicator that the structure quality may be low
5 ALERT type 4 Improvement, methodology, query or suggestion
1 ALERT type 5 Informative message, check

It is advisable to attempt to resolve as many as possible of the alerts in all categories. Often the minor alerts point to easily fixed oversights, errors and omissions in your CIF or refinement strategy, so attention to these fine details can be worthwhile. In order to resolve some of the more serious problems it may be necessary to carry out additional measurements or structure refinements. However, the purpose of your study may justify the reported deviations and the more serious of these should normally be commented upon in the discussion or experimental section of a paper or in the "special_details" fields of the CIF. checkCIF was carefully designed to identify outliers and unusual parameters, but every test has its limitations and alerts that are not important in a particular case may appear. Conversely, the absence of alerts does not guarantee there are no aspects of the results needing attention. It is up to the individual to critically assess their own results and, if necessary, seek expert advice.

Publication of your CIF in IUCr journals

A basic structural check has been run on your CIF. These basic checks will be run on all CIFs submitted for publication in IUCr journals (*Acta Crystallographica*, *Journal of Applied Crystallography*, *Journal of Synchrotron Radiation*); however, if you intend to submit to *Acta Crystallographica Section C* or *E* or *IUCrData*, you should make sure that full publication checks are run on the final version of your CIF prior to submission.

Publication of your CIF in other journals

Please refer to the *Notes for Authors* of the relevant journal for any special instructions relating to CIF submission.

PLATON version of 22/08/2024; check.def file version of 21/08/2024

Datablock Ylidanhydrate_MoKaDW_cut - ellipsoid plot

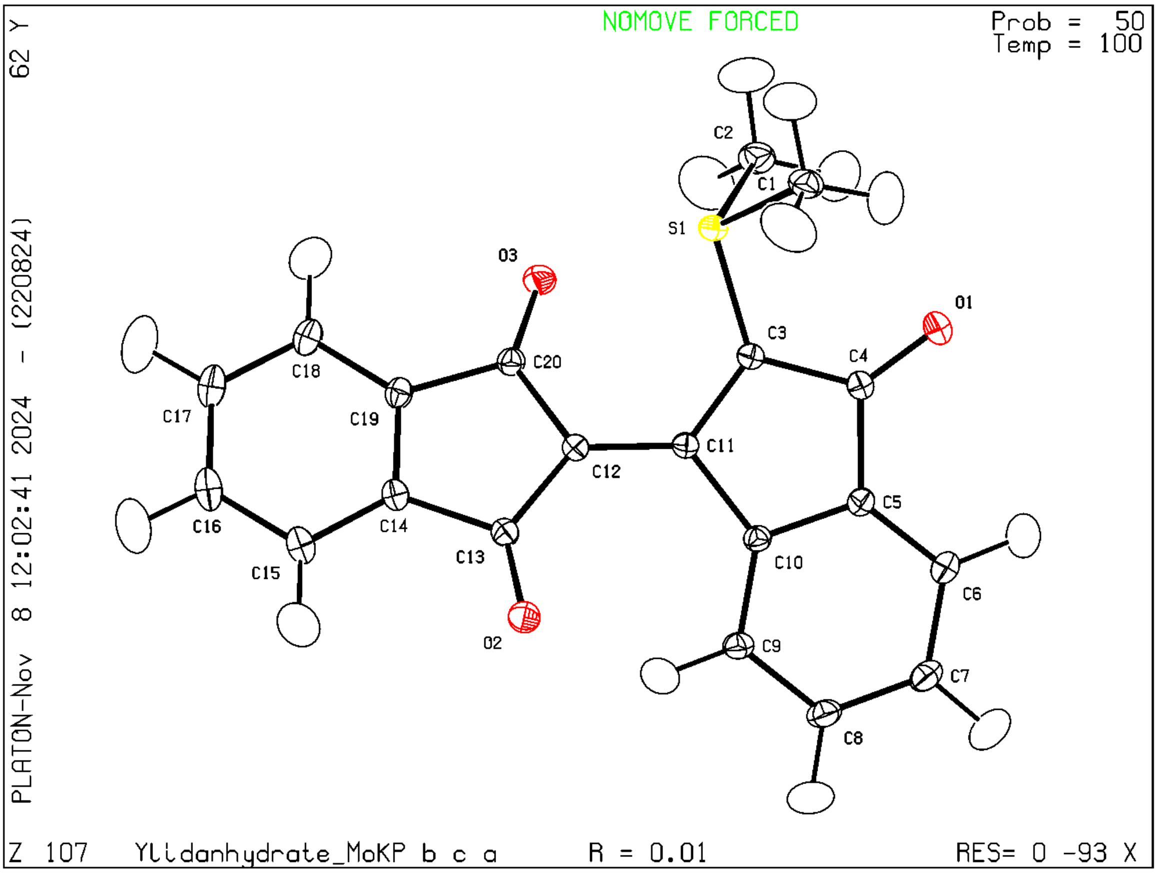

Supplement: Supplementary file 2 [file j-58-00678-sup2.zip › Ylidanhydrate_MoKaDW_cut_XRW_checkcif.pdf]
