# Supplementary material for: Synthesis and quantum crystallographic evaluation of WYLID: YLID’s red rival
Source: J Appl Crystallogr. 2025 Apr 4;58(Pt 3):678–87. doi: 10.1107/S160057672500175X (PMC12135977; doi:10.1107/S160057672500175X)

checkCIF/PLATON report

Structure factors have been supplied for datablock(s) Ylideanhydrate-20000eV

THIS REPORT IS FOR GUIDANCE ONLY. IF USED AS PART OF A REVIEW PROCEDURE FOR PUBLICATION, IT SHOULD NOT REPLACE THE EXPERTISE OF AN EXPERIENCED CRYSTALLOGRAPHIC REFEREE.

No syntax errors found. CIF dictionary Interpreting this report

Datablock: Ylideanhydrate-20000eV

Bond precision: C-C = 0.0002 A Wavelength=0.61980

Cell:	a=14.47953(6)	b=9.15279(5)	c=23.32492(9)
	alpha=90	beta=90	gamma=90
Temperature:	100 K		

	Calculated	Reported
Volume	3091.21(2)	3091.21(2)
Space group	P b c a	P b c a
Hall group	-P 2ac 2ab	-P 2ac 2ab
Moiety formula	C20 H14 O3 S	C20 H14 O3 S
Sum formula	C20 H14 O3 S	C20 H14 O3 S
Mr	334.37	334.40
Dx,g cm-3	1.437	1.437
Z	8	8
Mu (mm-1)	0.157	0.158
F000	1392.0	1393.3
F000’	1393.15	
h,k,lmax	23,14,37	23,14,37
Nref	6873	6713
Tmin,Tmax	0.961,0.969	0.411,1.000
Tmin’	0.961	

Correction method= # Reported T Limits: Tmin=0.411 Tmax=1.000
AbsCorr = MULTI-SCAN

Data completeness= 0.977 Theta(max)= 30.120

R(reflections)= 0.0110(6617) wR2(reflections)= 0.0299(6713)

S = 1.143 Npar= 344

The following ALERTS were generated. Each ALERT has the format
test-name_ALERT_alert-type_alert-level.
Click on the hyperlinks for more details of the test.

Alert level B

PLAT230_ALERT_2_B Hirshfeld Test Diff for S1 --C1 . 20.0 s.u.
PLAT230_ALERT_2_B Hirshfeld Test Diff for S1 --C2 . 20.5 s.u.
PLAT934_ALERT_3_B Number of (Iobs-Icalc)/Sigma(W) > 10 Outliers .. 3 Check
0 2 0, 2 2 0, 3 1 1,

Alert level C

PLAT918_ALERT_3_C Reflection(s) with I(obs) much Smaller I(calc) . 1 Check

Alert level G

ABSMU01_ALERT_1_G Calculation of _exptl_absorpt_correction_mu
not performed for this radiation type.
PLAT068_ALERT_1_G Reported F000 Differs from Calcd (or Missing)... Please Check
PLAT142_ALERT_4_G s.u. on b - Axis Small or Missing .... 0.00005 Ang.
PLAT143_ALERT_4_G s.u. on c - Axis Small or Missing .... 0.00009 Ang.
PLAT802_ALERT_4_G CIF Input Record(s) with more than 80 Characters 1 Info
PLAT910_ALERT_3_G Missing # of FCF Reflection(s) Below Theta(Min). 1 Note
0 0 2,
PLAT912_ALERT_4_G Missing # of FCF Reflections Above STh/L= 0.600 161 Note
PLAT933_ALERT_2_G Number of HKL-OMIT Records in Embedded .res File 1 Note
6 2 36,
PLAT969_ALERT_5_G The 'Henn et al.' R-Factor-gap value .... 3.266 Note
Predicted wR2: Based on SigI**2 0.92 or SHELX Weight 2.62
PLAT978_ALERT_2_G Number C-C Bonds with Positive Residual Density. 15 Info
PLAT979_ALERT_1_G NoSpherA2 Scattering Factors Used .... Please Note

---

0 **ALERT level A** = Most likely a serious problem - resolve or explain
3 **ALERT level B** = A potentially serious problem, consider carefully
1 **ALERT level C** = Check. Ensure it is not caused by an omission or oversight
11 **ALERT level G** = General information/check it is not something unexpected

3 ALERT type 1 CIF construction/syntax error, inconsistent or missing data
4 ALERT type 2 Indicator that the structure model may be wrong or deficient
3 ALERT type 3 Indicator that the structure quality may be low
4 ALERT type 4 Improvement, methodology, query or suggestion
1 ALERT type 5 Informative message, check

It is advisable to attempt to resolve as many as possible of the alerts in all categories. Often the minor alerts point to easily fixed oversights, errors and omissions in your CIF or refinement strategy, so attention to these fine details can be worthwhile. In order to resolve some of the more serious problems it may be necessary to carry out additional measurements or structure refinements. However, the purpose of your study may justify the reported deviations and the more serious of these should normally be commented upon in the discussion or experimental section of a paper or in the "special_details" fields of the CIF. checkCIF was carefully designed to identify outliers and unusual parameters, but every test has its limitations and alerts that are not important in a particular case may appear. Conversely, the absence of alerts does not guarantee there are no aspects of the results needing attention. It is up to the individual to critically assess their own results and, if necessary, seek expert advice.

Publication of your CIF in IUCr journals

A basic structural check has been run on your CIF. These basic checks will be run on all CIFs submitted for publication in IUCr journals (*Acta Crystallographica*, *Journal of Applied Crystallography*, *Journal of Synchrotron Radiation*); however, if you intend to submit to *Acta Crystallographica Section C* or *E* or *IUCrData*, you should make sure that full publication checks are run on the final version of your CIF prior to submission.

Publication of your CIF in other journals

Please refer to the *Notes for Authors* of the relevant journal for any special instructions relating to CIF submission.

PLATON version of 22/08/2024; check.def file version of 21/08/2024

Datablock Ylideanhydrate-20000eV - ellipsoid plot

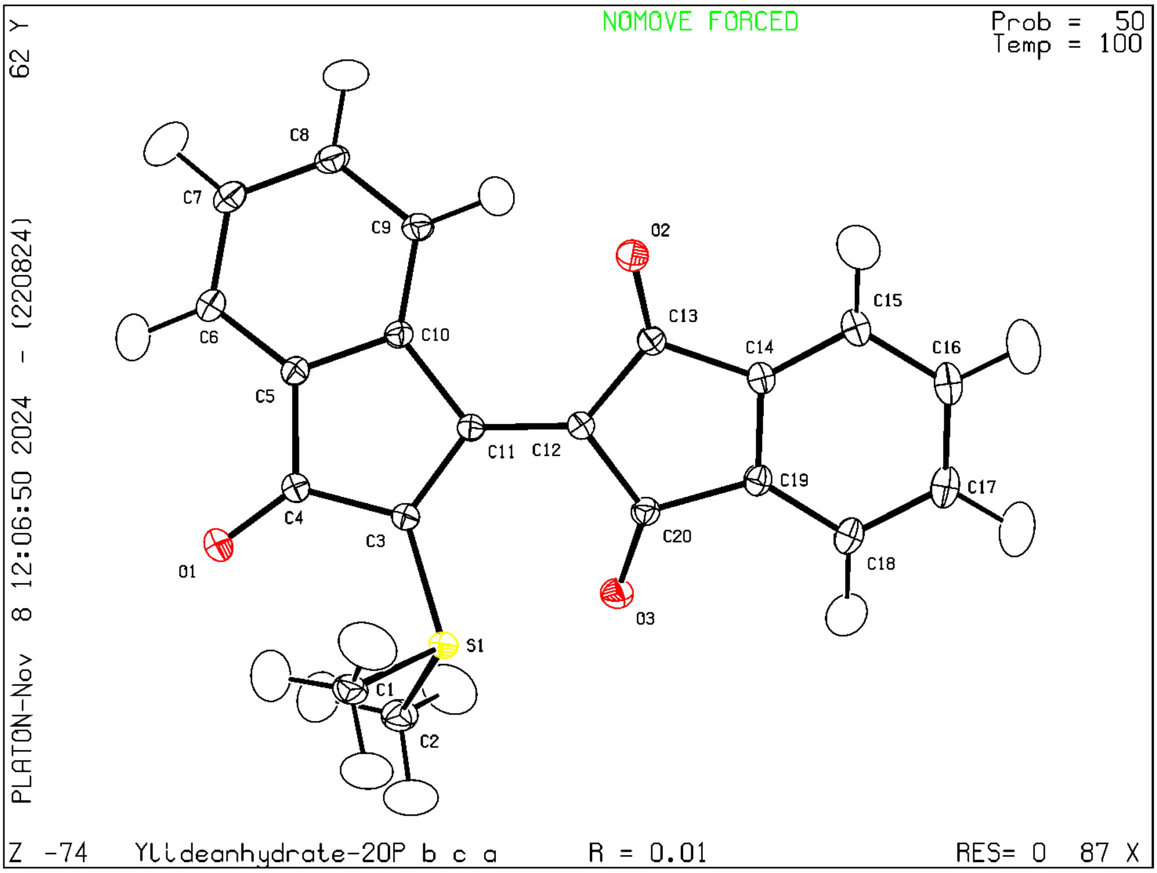

Supplement: Supplementary file 2 [file j-58-00678-sup2.zip › Ylideanhydrate-20000eV_checkcif.pdf]
