# Supplementary material for: Synthesis and quantum crystallographic evaluation of WYLID: YLID’s red rival
Source: J Appl Crystallogr. 2025 Apr 4;58(Pt 3):678–87. doi: 10.1107/S160057672500175X (PMC12135977; doi:10.1107/S160057672500175X)
Supplement: Supplementary file 3 [file j-58-00678-sup3.pdf]

JOURNAL OF APPLIED CRYSTALLOGRAPHY

Volume 58 (2025)

Supporting information for article:

Synthesis and quantum crystallographic evaluation of WYLID: YLID's red rival

Florian Meurer, Maximilian Schimpf, Birgit Hischa, Christoph Hennig, Julia Rehbein, Florian Kleemiss and Michael Bodensteiner

Supporting Information

S1. Synthesis ... 2
S1.1 General Information ... 2
S1.2 Synthetic Procedure ... 2
S2. Crystallographic Analysis ... 7
S2.1 Input for the X-ray restrained wavefunction fitting ... 10
S2.2 Model quality evaluation ... 12
S2.3 Quantum Crystallographic Analysis ... 18
S2.3.1 Laplacians of several bonds from XRW and MM ... 19
S2.3.2 Laplacians along the C-O Bonds from MM ... 21
S2.3.3 Topological Data at the BCPs from the XRW model ... 21
S2.4 Laplacians along the C-O bonds compared to 4-heptone ... 24
S2.5 Density Functional Theory Calculations of WYLID ... 25
S2.6 Comparison of saturated and unsaturated detector synchrotron data sets ... 28
S2.7 Comparison of HAR results between r2SCAN and ωB97X ... 32
S2.8 CSD search using MOGUL ... 34
S2.9 Hirshfeld Surface Analysis of WYLID ... 35
S2.10 Anomalous Dispersion Refinement ... 36
S2.11 Crystallographic Details of Bindandione ... 37
S3. References ... 38

S1. Synthesis

S1.1. General Information

All reactions were performed under a dry nitrogen atmosphere by using standard Schlenk techniques. DMSO and ethanol were dried over 4 Å and 3 Å molar sieves respectively. Other solvents were used without further purification or drying. Commercially available chemicals were purchased in high quality and used without further purification. For moisture-sensitive reactions, oven-dried glassware was used. NMR spectra were recorded at room temperature using a Bruker Avance 300 NMR spectrometer (300.13 MHz for ^{1}H, 75.47 MHz for ^{13}C). Mass spectra were recorded on an Agilent Q-TOF 6540 UHD instrument. Elemental analysis was performed using a Vario microcube. All UV-Vis spectra were measured using a SPECORD® 50 PLUS spectrometer from Analytik Jena with an optical path length of 10 mm. IR spectra were recorded on a Cory 630 FTIR from Agilent Technologies.

S1.2. Synthetic Procedure

([1,2']biindenylidene-3,1',3'-trione), Bindandione

A solution of dry ethanol (24 ml) and 1,3-indandione (2.04 g, 14.0 mmol, 1 equiv.) was prepared in an oven-dried Schlenk tube and stirred for 5 min. Sodium acetate (1.53 g, 18.6 mmol, 1.3 equiv.) was added and the mixture was stirred for 1 h at room temperature. The solution was then diluted with water (50 ml) and acidified with HCl to a pH of about 1. The precipitate was filtered off and dried under vacuum to give a green powder (1.67 mg, 43 %).

^{1}H NMR (300.13 MHz, $CDCl_3$): δ 9.67 (m, 1H), 8.04-7.99 (m, 1H), 7.99-7.92 (m, 2H), 7.89-7.84 (m, 1H), 7.84-7.79 (m, 2H), 7.78-7.70 (m, 1H), 4.16 (s, 2H); ^{13}C NMR (75.47 MHz, $CDCl_3$): δ 201.0, 191.1, 189.4, 155.4, 145.9, 141.7, 141.3, 140.4, 135.4, 134.2, 131.7, 125.9, 123.5, 123.4, 123.1, 43.5

Crystals suitable for X-ray diffraction experiments were grown by applying the vapour diffusion method. For this purpose, a concentrated solution of Bindandione in benzene was filled into a Schlenk tube, which was connected via joints to a small flask containing diethyl ether. The setup was then placed in a refrigerator at 6°C and left until crystals formed.

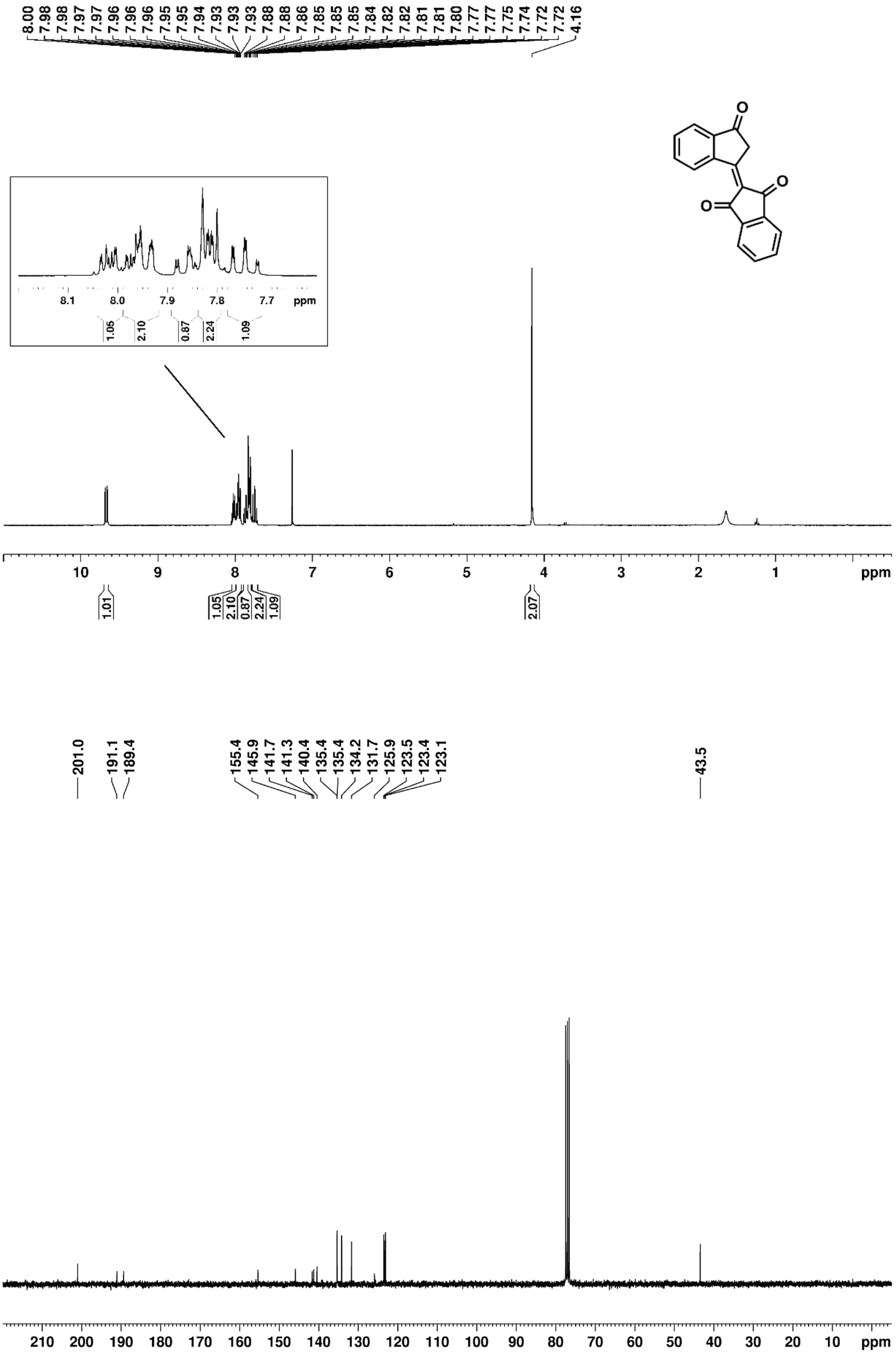

Figure S1: 1H-NMR (top) and 13C-NMR (bottom) spectra of Bindandione.

2-(dimethyl-λ4-sulfaneylidene)-[1,2'-biindenylidene]-1',3,3'(2H)-trione, WYLID

Bindandione (200 mg, 0.73 mmol, 1 equiv.) was suspended in acetic anhydride (2 ml) in an oven-dried Schlenk tube under a dry N_2 atmosphere. DMSO (71 µL, 1.4 equiv.) was added and the reaction mixture was stirred at 85°C for 4 h. At the end of the reaction time, the solution was allowed to cool to room temperature and then quenched with potassium carbonate. DCM and water were added in equal parts (20 ml) and transferred to a separating funnel. The aqueous phase was then extracted with DCM (3x 20 ml) and the collected organic phases were dried over $MgSO_4$. The drying agent was filtered off and the solution was then dried under vacuum to give a dark red solid. Recrystallization from ethyl acetate/petroleum ether afforded WYLID as a bright red crystalline powder (80.4 mg, 33%).

^{1}H NMR (300.13 MHz, $CDCl_3$): δ 8.42 (m, 1H), 7.80 (m, 1H), 7.69 (m, 1H), 7.61 (m, 2H), 7.53 (m, 1H), 7.47 (m, 1H), 7.34 (m, 1H), 3.25 (s, 6H); ^{13}C NMR (75.47 MHz, $CDCl_3$): δ 190.3, 163.3, 140.6, 134.9, 132.9, 131.1, 128.4, 121.6, 120.8, 109.1, 86.7, 27.9.

HR-ESI-MS: $(C_{20}H_{13}O_3S+H)^+$: m/z_{calc} = 335.0736, m/z_{found} = 335.074

EA: Anal. Calcd. (%) for $C_{20}H_{13}O_3S$: C 71.84, H 4.22, S 9.59; found: C 71.66, 4.33, S 8.59

IR: IR (solid-state): ν / cm^{-1} = 3012, 1655, 1625, 1580, 1510, 992, 738.

Crystals suitable for X-ray diffraction experiments were grown by the vapour diffusion method. Therefore, a concentrated solution of WYLID in benzene was added in a Schlenk tube, which was connected to a small flask containing diethyl ether. The setup was the placed in a refrigerator at 6°C and left until crystals suitable for the diffraction experiments were formed.

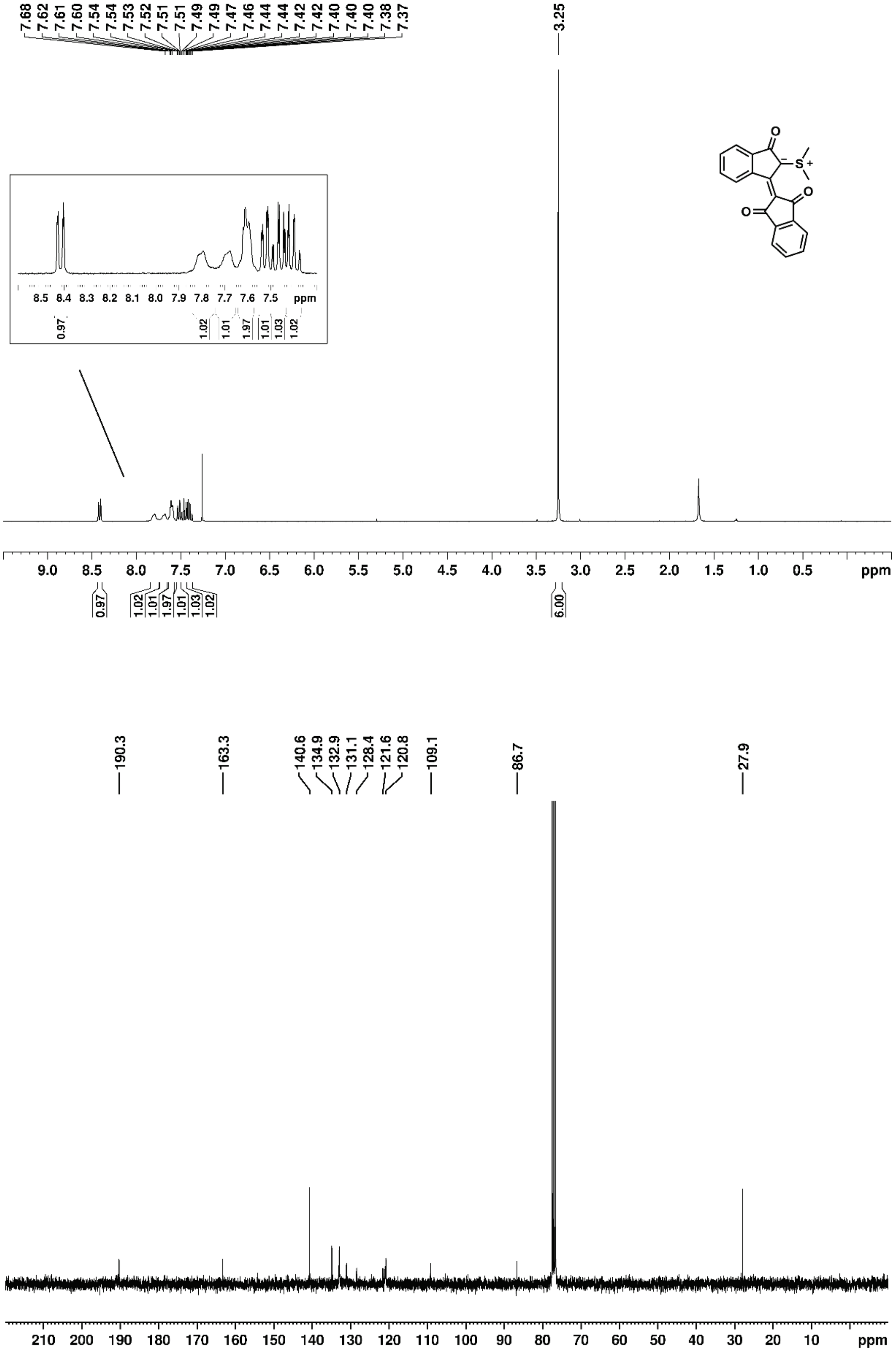

Figure S2: 1H-NMR (top) and 13C-NMR (bottom) spectra of WYLID.

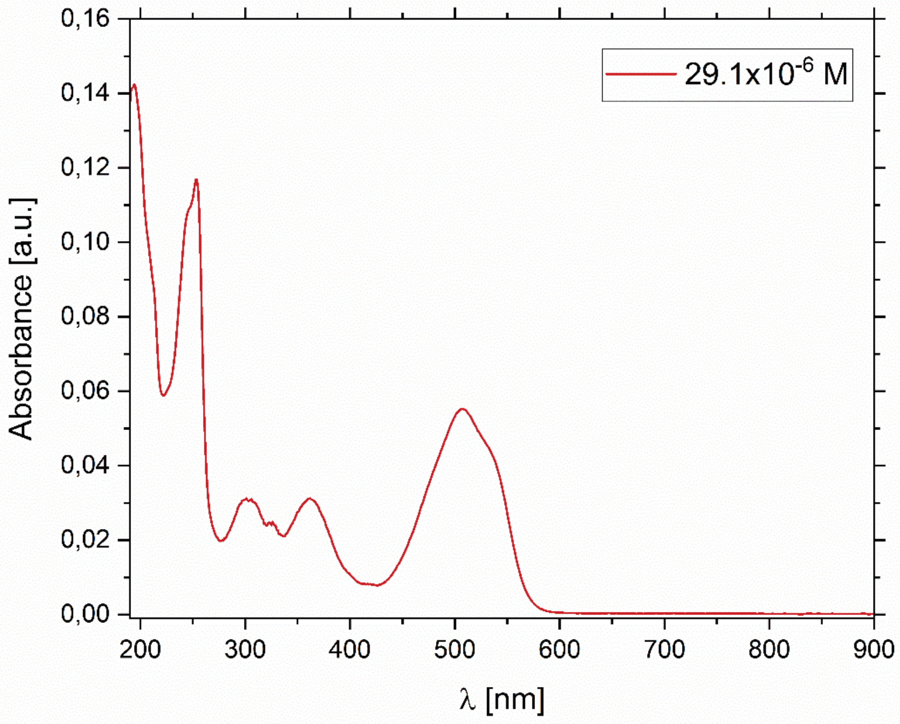

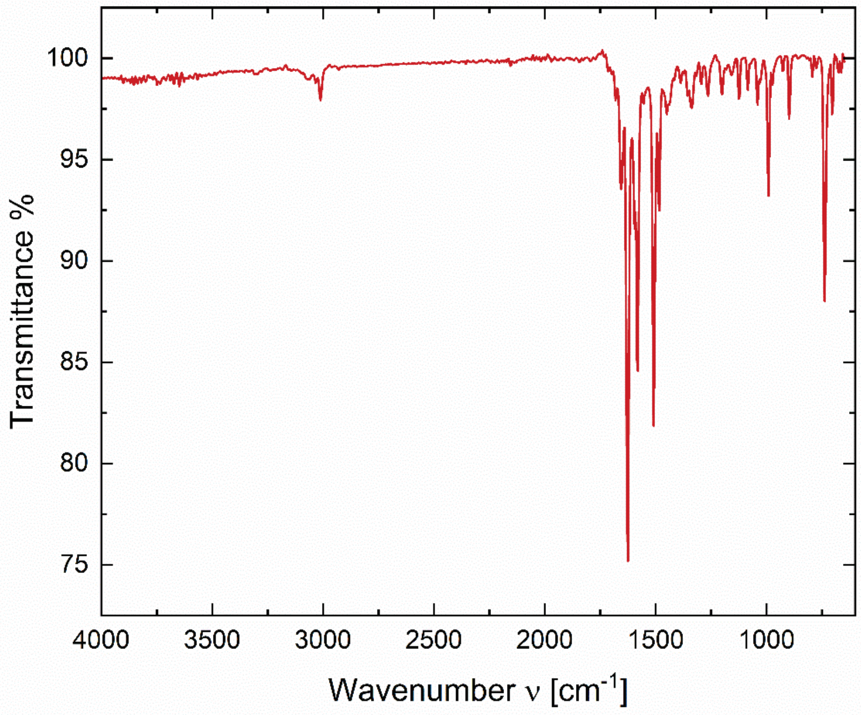

Figure S3: UV/VIS (top) and IR (bottom) spectra of WYLID.

S2. Crystallographic Analysis

All data sets were processed and integrated using *CrysAlisPro* V.43 (Rigaku Oxford Diffraction Ltd, 2019). The initial structure solution for each model was obtained using ShelXT (Sheldrick, 2015). Structural refinement for all models except for the multipolar models was performed using *Olex2* software (Dolomanov *et al.*, 2009) with *olex2.refine* (Bourhis *et al.*, 2015) as the refinement engine. All HAR models were obtained by using *NoSpherA2* (Kleemiss *et al.*, 2021) with the quantum chemical software *ORCA5* (Neese *et al.*, 2020). All calculations were performed using the r2SCAN/def2-TZVP (Weigend & Ahlrichs, 2005; Furness *et al.*, 2020) method/basis set combination unless otherwise noted (see below for the comparison with the hybrid functional, ωB97X). The parameters set in *NoSpherA2* / *ORCA* for the calculations were a normal integration accuracy, the *NoSpherA2* custom SCF threshold, a normal convergence strategy in the SCF procedure and a toluene or DMSO solvation model. In this work, neither the choice of a solvation model nor the inclusion of the nearest neighbour molecules to include interatomic interactions significantly changed the results of the HAR procedure. In all models, we used the r2SCAN method and employed a toluene solvation model. The HAR procedure (Capelli *et al.*, 2014) was performed iteratively, switching between the single-point wavefunction calculation in *ORCA*, adjusting the new atomic form factors using *NoSpherA2* and the Hirshfeld stockholder partitioning scheme (Hirshfeld, 1977), and then iteratively updating the atomic coordinates and anisotropic displacement parameters until convergence was reached.

The multipolar models and visualization of their topological analysis were performed using the *MoPro*, *VMoPro* (Jelsch *et al.*, 2005) and the *MoProViewer* (Guillot, 2012) software packages. Refinement was conducted against the squared structure factors F^2. We employed positional and ADPs restraints on all H-atoms according to the freely refined parameters from the HAR procedure on the same data set.

The X-ray restrained wavefunction fitting was performed using the *TONTO* software (Jayatilaka & Grimwood, 2003). The input file for the calculation in *TONTO* is given in S2.1. The XRW procedure was run starting with the HAR geometry and increasing the perturbation by the agreement of measured and calculated intensities by a weight step size of 0.01 ("λ-step"). The procedure was terminated already after the 8th step ($\lambda = 0.07$).

Throughout the analysis, the first step of the XRW, without any X-ray diffraction contribution is referred to as the "HF model". This is different from the "DFT model", which is based on a gas-phase optimized (r2SCAN-3//r2SCAN/def2-TZVP (Gasevic *et al.*, 2022)) DFT calculation. All wavefunction-based models were investigated using the *multiwfn* software. (Lu & Chen, 2012)

The data sets using Cu K_{α} and Mo K_{α} radiation were collected with a Rigaku Oxford Diffraction Synergy-DW rotating anode diffractometer with HyPix Arc 150° photon counting detector.

The data set using Cu K_β radiation was recorded on a Rigaku Oxford Diffraction SuperNova system using an Oxford Instruments microfocus tube and specially optimized Axo Dresden Cu K_β multilayer optic as well as an Atlas S2 CCD detector.

Table S1 shows the crystallographic information for each of the quantum crystallographic models of the different wavelength structures.

Table S1: Crystallographic information of data sets and quality parameters of the models. Structures used within this work were deposited in the CCDC with merged measured structure factors. The raw data and structures can be obtained from zenodo.org using the DOI provided. The models can be found in the respective "struct" directories, starting with the model name "IAM", "HAR", "MM" or "XRW".

Radiation (λ / Å)	**Cu Kα** (1.54187)	**Cu Kα** (1.39222)	**Mo Kα** (0.71073)	**Synchrotron** (0.56356)
Data DOI	10.5281/zenodo.14699349	10.5281/zenodo.14699464	10.5281/zenodo.14699544	10.5281/zenodo.14723884
Formula	$C_{20}H_{14}O_3S$			
Mass / g mol^{-1}	334.40			
Crystal size / mm^3	0.079 x 0.083 x 0.160	0.113 x 0.184 x 0.238	0.079 x 0.083 x 0.160	0.10 x 0.10 x 0.10
Space group	*Pbca*			
a / Å	14.47962(7)	14.4765(2)	14.49062(7)	14.54072(7)
b / Å	9.15557(4)	9.1539(1)	9.1569(4)	9.17361(2)
c / Å	23.2502(19	23.3338(4)	23.3290(1)	23.40310(7)
V / Å^3	3093.50(3)	3092.11(6)	3095.51(2)	3121.7(2)
D / g cm^{-3}	1.436	1.437	1.435	1.423
μ / mm^{-1}	1.989	1.447	0.224	0.122
θ range / °	3.79-67.68	4.39-76.18	2.22-40.48	2.24-40.40
T / K	100	100	100	100
Resolution / Å	0.80	0.72	0.55	0.55
$I/\sigma(I)$	208.1	49.3	138.0	95.3
R_{int} / %	2.69	7.67	2.71	4.88
Total reflections	187273	78182	527077	508696
Unique reflections > 4σ	3130	4227	8605	8745
Completeness / %	100.0	100.0	99.9	100.0
**HAR**				
CCDC Nr.	2392768	2392769	2392771	2392770
Data set	"data_Ylidanhydrate-CuKaDW"	"data_WYlid_Large6_Cub_100K_fm2"	"data_Ylidanhydrate_MoKaDW_cut"	"data_WYLID-4_22000_abs"
Nr. refined parameters	343	343	418	444
Goodness-of-fit	1.118	1.068	1.011	1.070
R indices, $I > 2\sigma$ / %	0.88	3.89	1.10	1.00
R indices (all data) / %	0.92	4.09	1.61	1.39
wR2 indices / %	1.94	10.00	1.43	1.83
Diff. peak, hole / e Å^{-3}	0.065, -0.100	0.362, -0.332	0.131, -0.121	0.106, -0.117
**MM**				
CCDC Nr.	-	-	2393562	2393564
Data set	-	-	"data_Ylidanhydrat-MoKADW2_MM"	"data_WYLID_22keV_MM"
Nr. refined parameters	-	-	756	756
Goodness-of-fit	-	-	1.06	1.02
R indices, $I > 2\sigma$ / %	-	-	1.27	1.32
R indices (all data) / %	-	-	1.59	1.36
wR2 indices / %	-	-	1.34	1.02
Diff. peak, hole / e Å^{-3}	-	-	0.182, -0.171	0.143, -0.169
**XRW**				
Data set	-	-	"data_Ylidanhydrate_MoKaDW_XRW"	-
Goodness-of-fit	-	-	1.43	-
R indices, $I > 2\sigma$ / %	-	-	0.85	-
wR2 indices / %	-	-	0.76	-
Diff. peak, hole / e Å^{-3}	-	-	0.132, -0.114	-

S2.1. Input for the X-ray restrained wavefunction fitting

```
{

  charge= 0
  multiplicity= 1

  ! Process the CIF
  cif= {
    file_name= ./WYLID_MoKa_small.cif
  }
  process_cif

  name= Ylideanhydrate-MoKa-XRW

  basis_directory= /home/xrz/tonto/basis_sets/
  basis_name= def2-TZVP

  dispersion_coefficients= {
   C 0.0035 0.00160
   H 0.00000 0.00000
   O 0.0116 0.0061
   S 0.1242 0.1244
  }

  crystal= {
    xray_data= {
      thermal_smearing_model= hirshfeld
      partition_model= mulliken
      optimise_extinction= false
      correct_dispersion= TRUE
      optimise_scale_factor= true
      wavelength= 0.6198 angstrom

      REDIRECT ./WYLID_MoKa_merged.hkl

      f_sigma_cutoff= 4.0
    }
  }

  ! Geometry
  put

  ! Tight grid
  becke_grid = {
    set_defaults
    accuracy= normal
    pruning_scheme= jayatilaka2
  }

  ! More accuracy
  output_style_options= {
    real_precision= 8
    real_width= 20
  }

  ! Normal SCF
  scfdata= {
    initial_density= promolecule
    kind=           rhf
    use_SC_cluster_charges= FALSE
    convergence= 0.001
    linear_dependence_tol= 0.001
    diis= {                   ! This is the extrapolation procedure
      save_iteration=  1
      start_iteration= 3
      keep=           10
      convergence_tolerance= 0.09
    }
    max_iterations=  200         ! The maximum number of SCF iteration

    use_damping=     YES         ! These are used to damp the SCF iteration process
    damp_factor=     0.80        ! by including 60% of the previous result
    damp_finish=     7
    use_level_shift= NO
  }
  scf

  scfdata= {
    initial_density=   restricted
    kind=              xray_rhf
    direct=            yes
    convergence=       0.00001
    linear_dependence_tol= 0.001
    use_SC_cluster_charges= FALSE
```

```
    diis= {                    ! This is the extrapolation procedure
      save_iteration=  1
      start_iteration= 3
      keep=            10
      convergence_tolerance= 0.09
    }
    max_iterations=  200         ! The maximum number of SCF iteration
    do_outputs= TRUE

    use_damping=     YES         ! These are used to damp the SCF iteration process
    damp_factor=     0.80        ! by including 60% of the previous result
    damp_finish=     7
    use_level_shift= YES
    initial_lambda=  0.000       ! These specify the "lambda value"
    lambda_step=     0.010       ! used to mix the energy with the chi^2
    lambda_max=      2.000
  }
  scf
}
```

S2.2. Model quality evaluation

Cu K_{α}

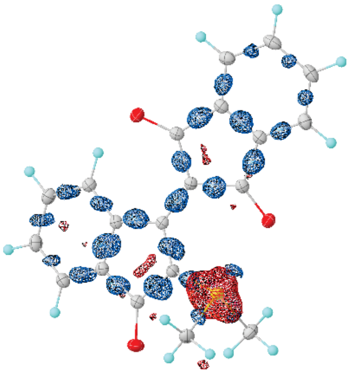

Cu K_{β}

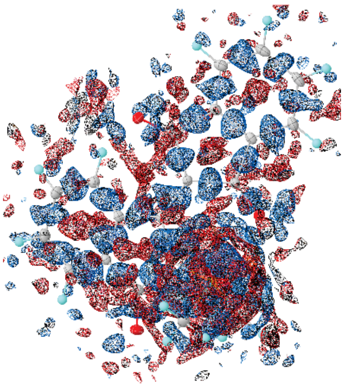

Mo K_{α}

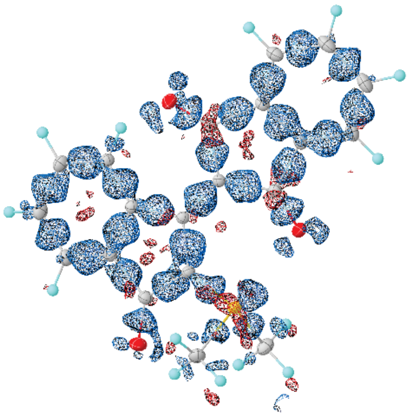

Synchrotron

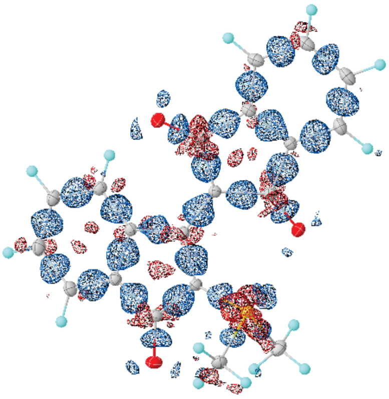

Figure S4a: Residual electron density of the independent atom models for each data set at the 0.15 e/Å^3 iso level projected onto the asymmetric unit. Blue indicates positive, red negative residual electron density. Non-hydrogen atoms are shown with a 50% probability.

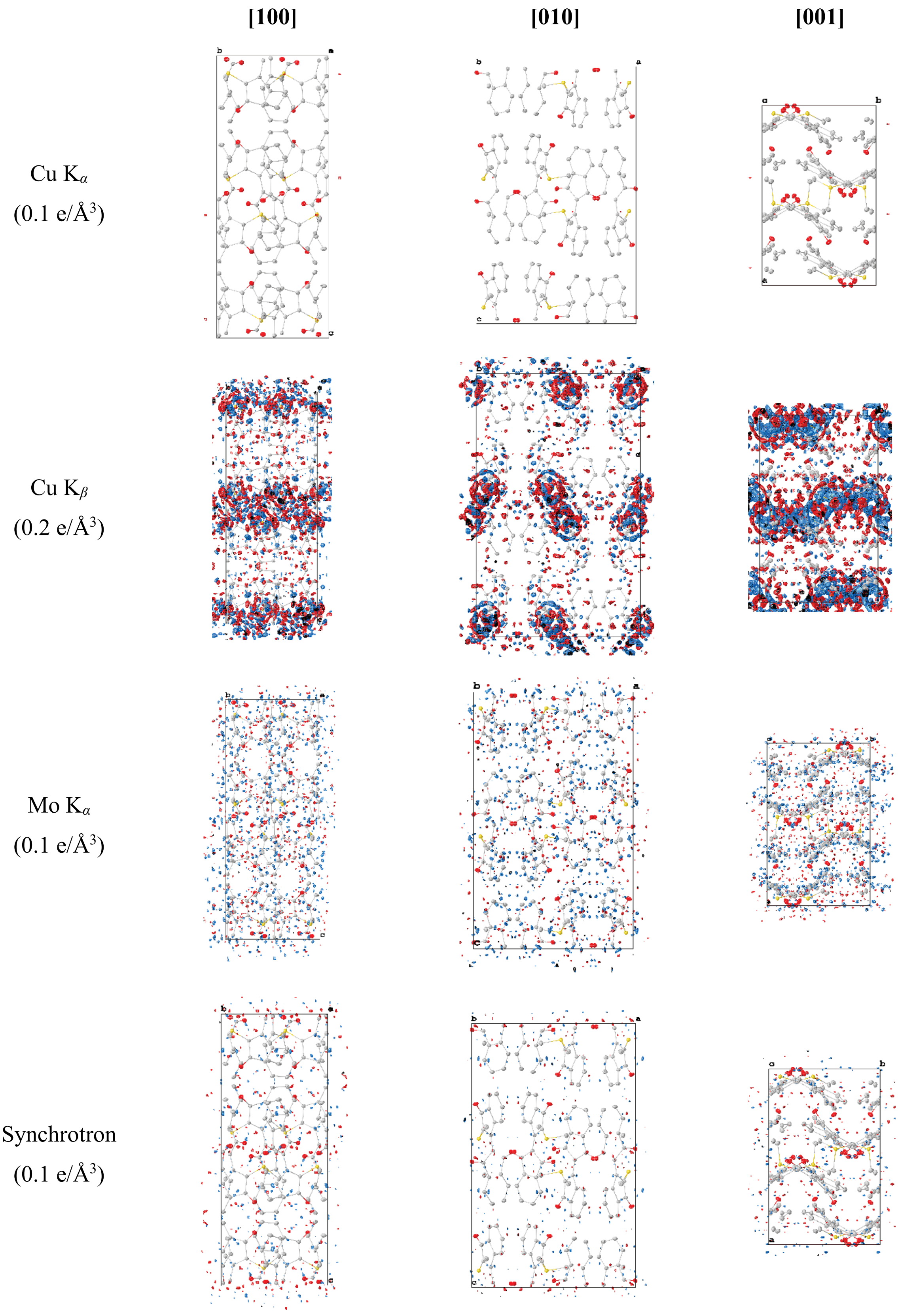

Figure S4b: Residual electron densities of the HAR models along each crystallographic axis with the iso level indicated below each wavelength. Blue indicates positive, red negative residual electron density. All atoms are shown with 50% probability. Hydrogen atoms have been omitted for clarity.

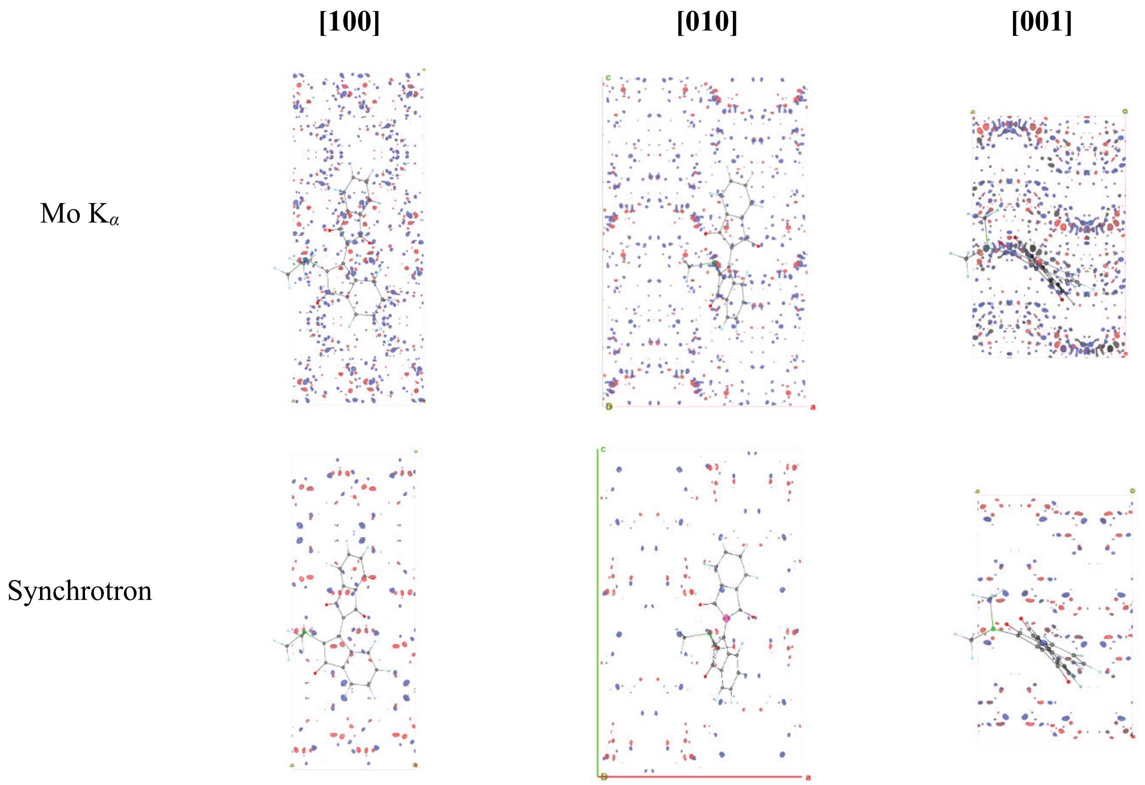

Figure S5: Residual electron densities of the multipole models along each crystallographic axis at a 0.1 e/Å^3 iso level. Blue indicates positive, red negative residual electron density. All atoms are shown with 50% probability. Hydrogen atoms have been omitted for clarity.

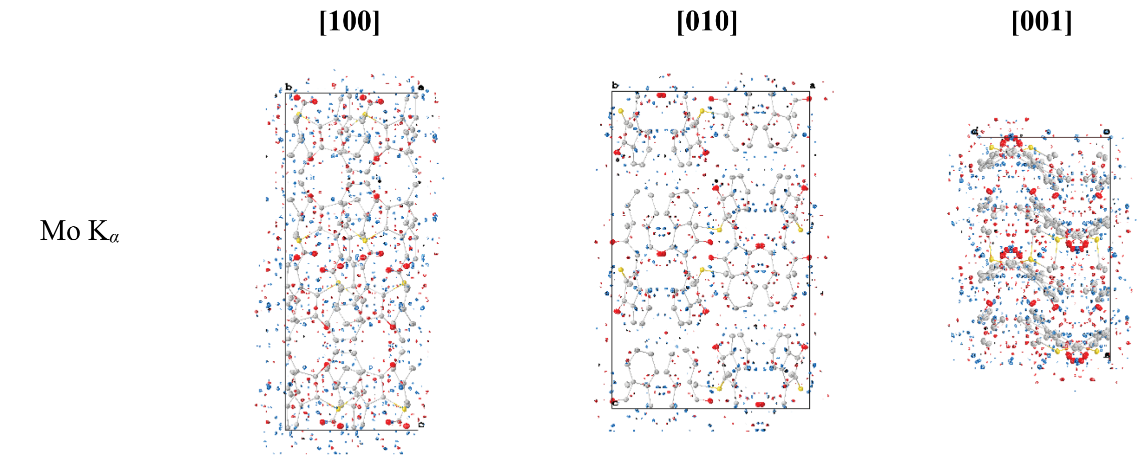

Figure S6: Residual electron densities of the XRW model along each crystallographic at an 0.1 e/Å^3 iso level. Blue indicates positive, red negative residual electron density. All atoms are shown with 50% probability ellipsoids. Hydrogen atoms have been omitted for clarity.

The residual electron density in Figures S4-S6 is generally low for all models, except the Cu K_β based models. We attribute this to the much weaker Cu K_β raw intensity compared to the Cu K_α / Mo K_α

rotating anode and synchrotron radiation. The residual density in the Cu K_β models is mostly concentrated near the sulfur atom, which is the heaviest atom in the structure.

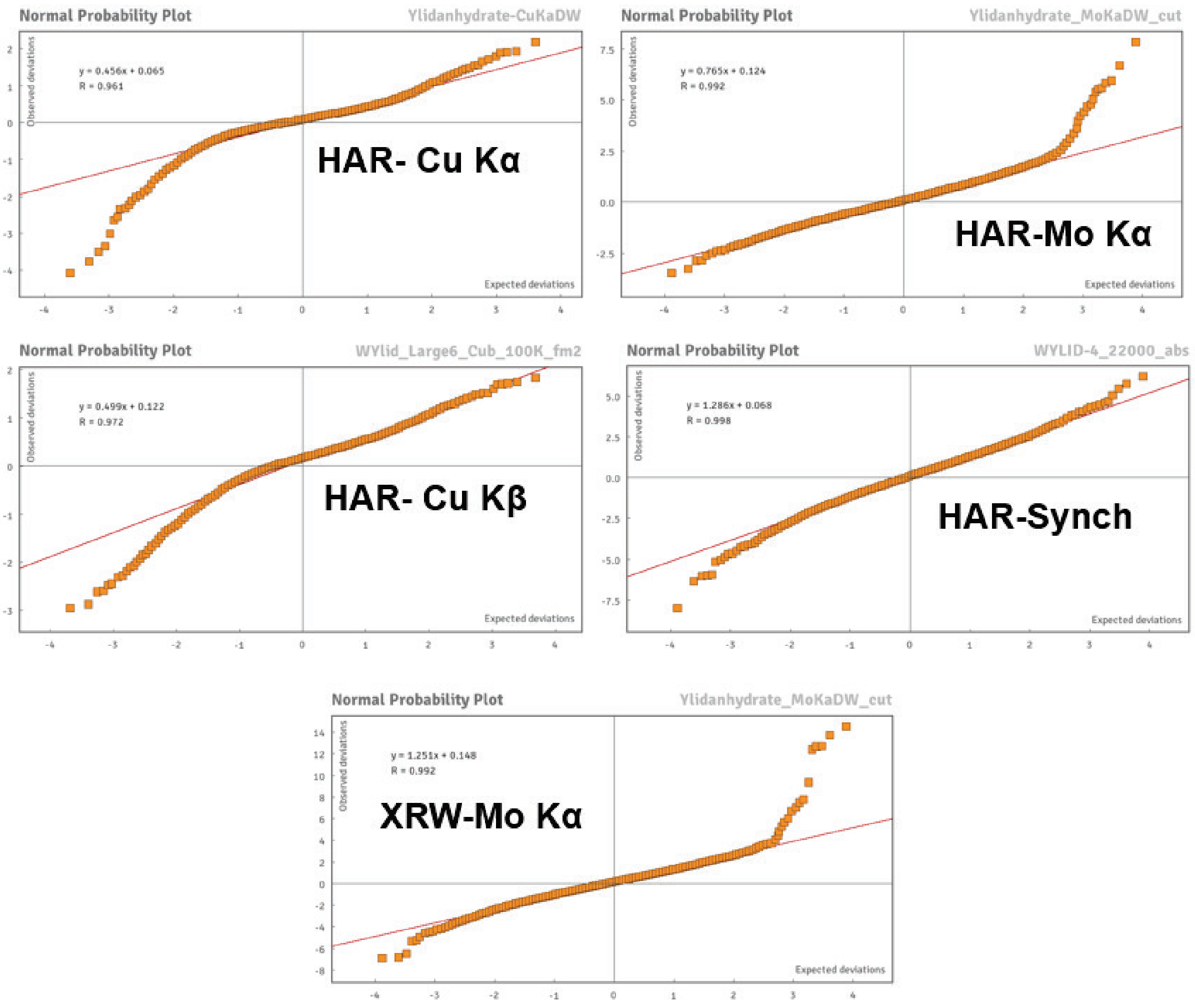

Figure S7: Normal probability plots of the wavefunction based quantum crystallographic models of WYLID.

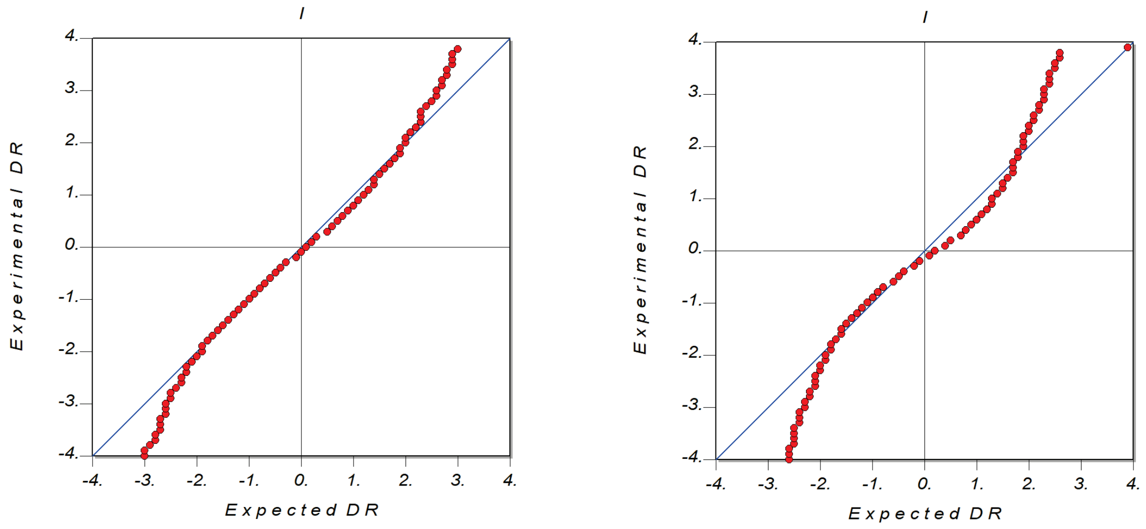

Figure S8: Normal probability plots of the multipolar models of Mo K_{α} (left) and synchrotron (right).

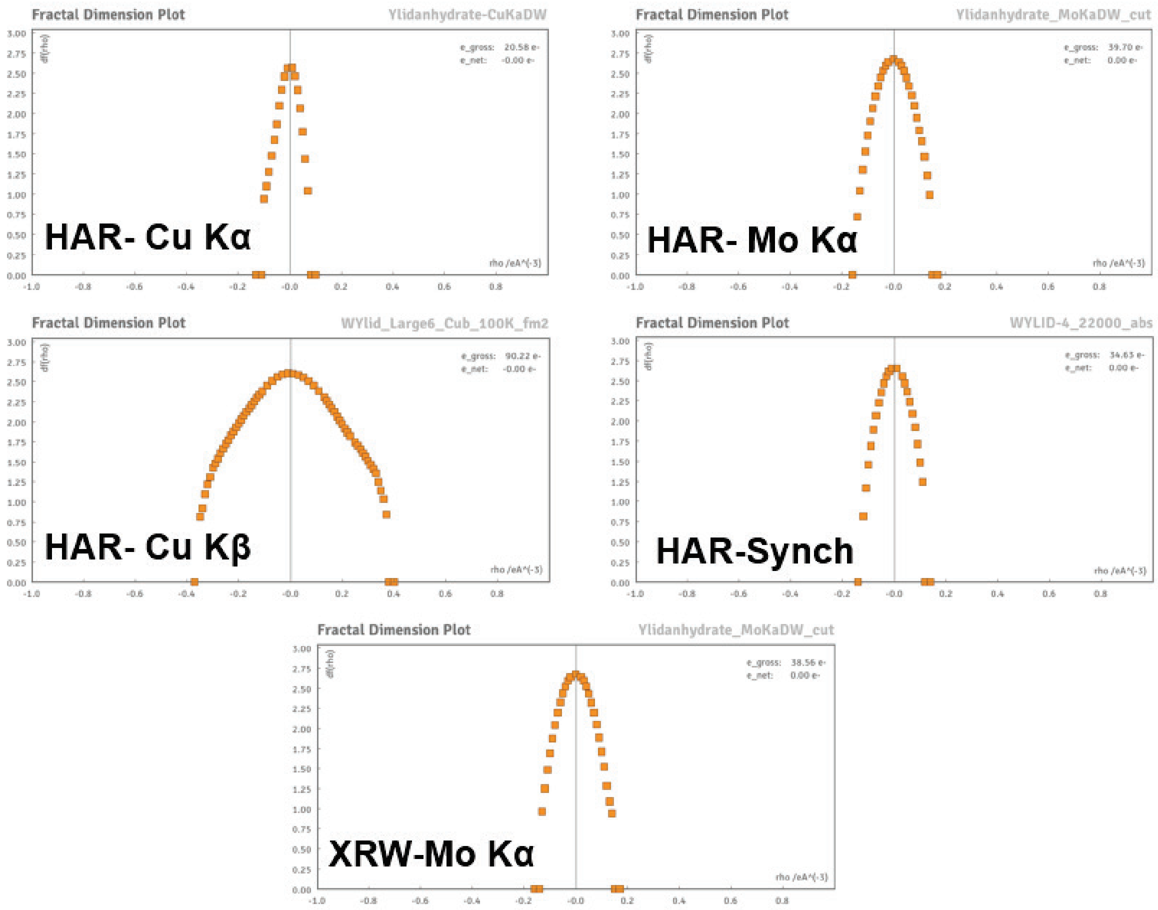

Figure S9: Fractal dimension plots according to Meindl & Henn (2008) of the wavefunction based models of WYLID.

S2.3. Quantum Crystallographic Analysis

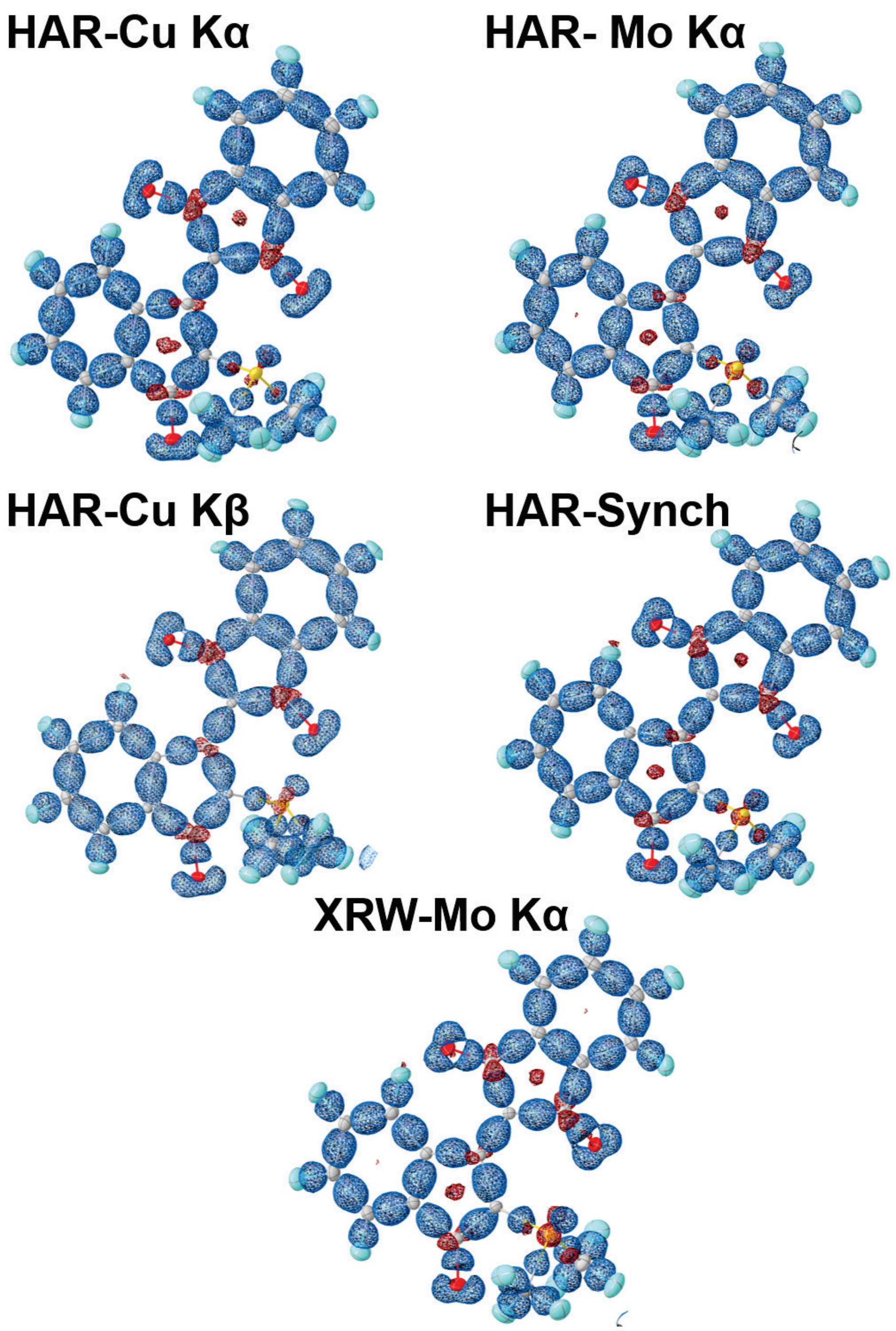

Figure S10: Deformation densities of the wavefunction based models of WYLID at the 0.15 e/Å^3 iso surface. Blue indicates positive, red negative residual electron density. Atoms are drawn at a 50% probability.

The deformation density maps (Fig. S10) reveal the valence interaction in WYLID, that the independent atom model could not describe. In particular, the oxygen lone-pairs and the delocalized π-system, π-holes, along the p-holes on the carbonyl carbons are well visible. Despite the lower quality of the data used in the Cu Kβ model, the deformation density is very similar to the models based on the best possible data sets. This underscores the fact that HAR does not necessitate the use of

high-resolution perfect-quality data, which is typically required for multipolar modelling in order to obtain accurate quantum chemical insights.

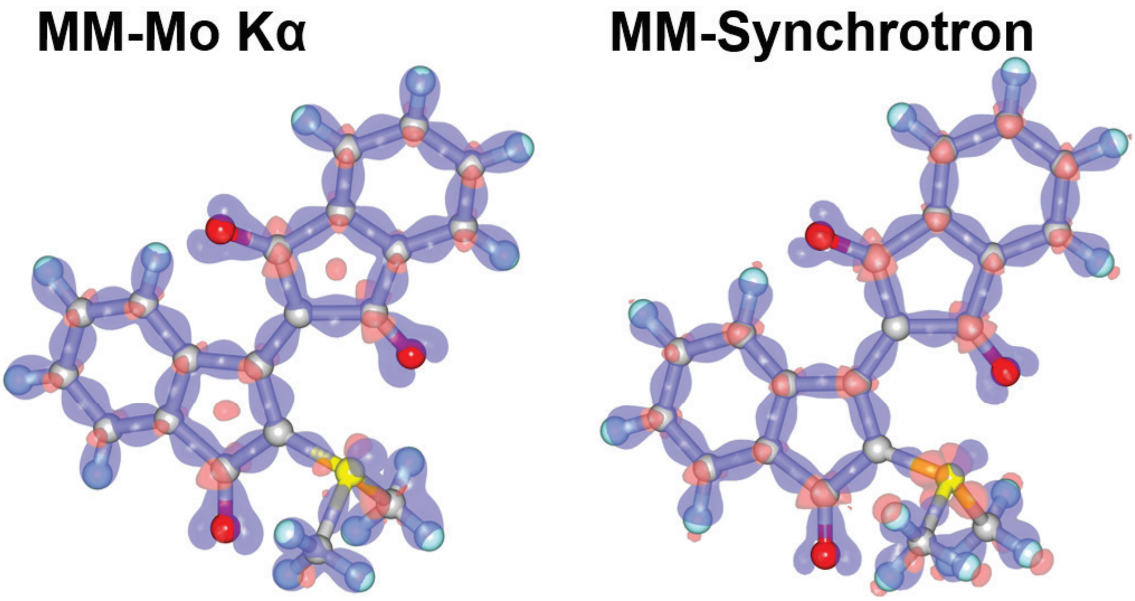

Figure S11: Deformation densities of the MM models of WYLID at the 0.15 e/Å^3 iso surface. Blue indicates positive, red negative residual electron density. Atoms are drawn at a 50% probability.

S2.3.1. Laplacians of several bonds from XRW and MM

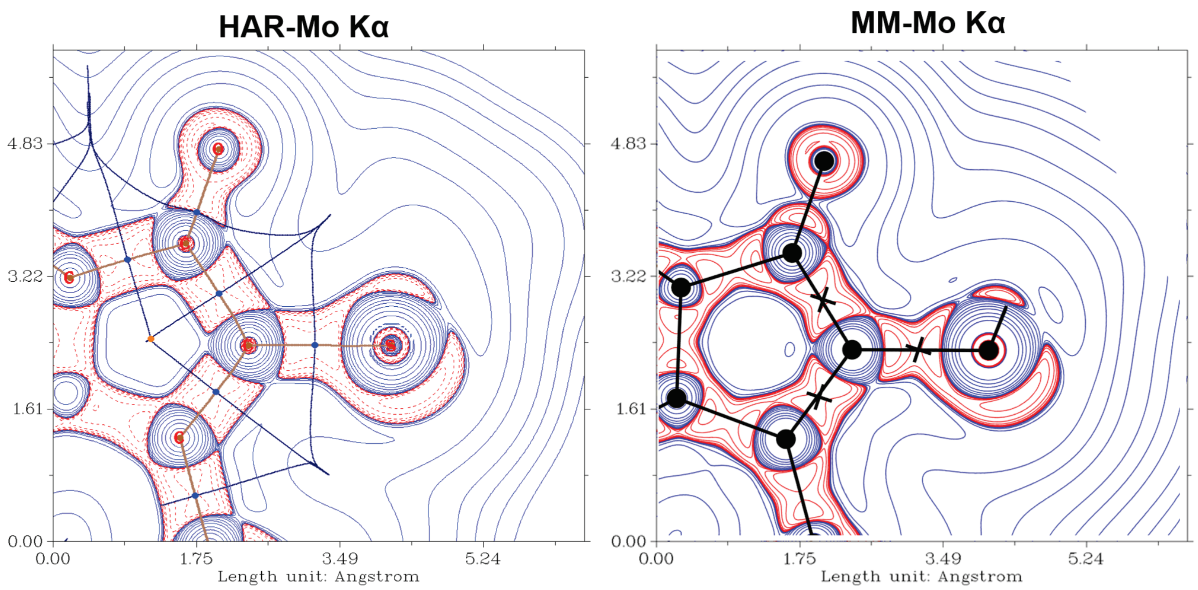

Figure S12: Laplacian (e·Å^{-5}) of the total electron density around the S1-C3 and C4-O1 bonds from the XRW model (left) and MM model (right) of the Mo Kα data set. Contour lines are shown at a logarithmic iso-level to a total of 62 iso-levels. Red indicates a positive and therefore VSCD, blue a negative value of the Laplacian and therefore VSCC. In the left image, BCPs are shown in blue and RCPs in orange, while on the right only the three BCPs from C3 are shown.

Overall, the Laplacian of the total electron density is of similar shape for the MM and XRW models of the Mo Kα data set. The main difference is found in the C4-O1 bond, where in the MM model, the Laplacian shows a different structure, reaching positive values after the BCP, while in the XRW model, the Laplacian stays in the negative from C to O.

S2.3.2. Laplacians along the C-O Bonds from MM

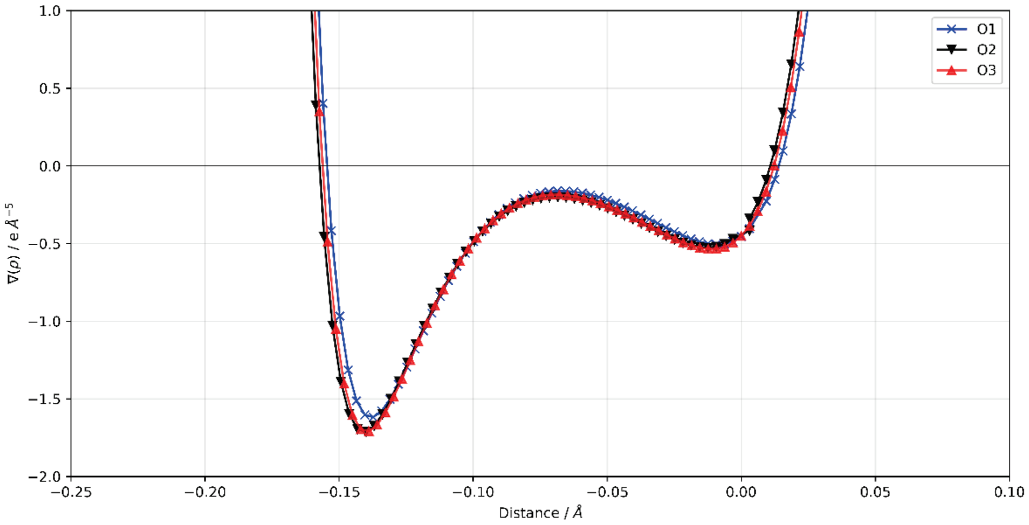

Figure S13: Laplacian (e·Å^{-5}) of the total electron density along the carbonyl bonds from the MM-Mo K$_{\alpha}$ model.

S2.3.3. Topological Data at the BCPs from the XRW model

Table S2: Information the BCPs compared between the MM and XRW model analogous to Table 3 in the main text.

	MM-Mo K$_\alpha$		XRW-Mo K$_\alpha$	
Atom pair	ρ_{BCP} / eÅ^{-3}	$\nabla(\rho_{BCP})$ / eÅ^{-5}	ρ_{BCP} / eÅ^{-3}	$\nabla(\rho_{BCP})$ / eÅ^{-5}
S1-C3	1.42	-6.81	1.50	-12.6
S1-C2	1.28	-4.95	1.37	-10.7
S1-C1	1.26	-4.36	1.35	-9.69
O1-C4	2.79	-28.25	2.71	-0.55
O2-C13	2.78	-27.96	2.71	-0.51
O3-C20	2.79	-25.55	2.70	-0.51
C11-C12	2.11	-18.26	2.14	-0.24

Table S3: Bader charges in electrons for the HAR models based on the different wavelength data sets. Charges calculated by the *multiwfn* software. (Lu & Chen, 2012)

	Cu K_{α}	Cu K_{β}	Mo K_{α}	Synchrotron
S1	0.469147	0.455535	0.470229	0.463612
O3	-1.17921	-1.18082	-1.18519	-1.17387
O2	-1.17239	-1.17688	-1.19703	-1.18106
O1	-1.18601	-1.18962	-1.19767	-1.16967
C20	0.962802	0.965077	0.960325	0.958144
C11	0.023775	0.024566	0.021741	0.024433
C12	-0.08348	-0.08699	-0.05147	-0.08457
C19	-0.04938	-0.05175	-0.08643	-0.05093
C3	-0.24157	-0.24082	-0.05039	-0.23717
C5	-0.0466	-0.04725	-0.02898	-0.04737
C9	-0.02307	-0.01702	-0.03355	-0.02929
C10	-0.02812	-0.02938	-0.23764	-0.02789
C13	0.988571	0.99575	0.989896	0.987584
C14	-0.04896	-0.05175	-0.05591	-0.04997
C4	0.989865	0.993727	0.992493	0.984101
C6	-0.02864	-0.01989	-0.03294	-0.03023
C15	-0.02400	-0.02077	-0.02932	-0.02538
C18	-0.02683	-0.02242	-0.03317	-0.03124
C7	-0.03416	-0.03024	-0.0326	-0.03514
C1	-0.15599	-0.11184	-0.15888	-0.16027
C8	-0.04022	-0.02973	-0.04618	-0.04432
C17	-0.03407	-0.03021	-0.03318	-0.03449
C2	-0.14106	-0.10830	-0.14198	-0.14135
C16	-0.03499	-0.01958	-0.03774	-0.04009
H6	0.0734	0.06554	0.077099	0.076201
H7	0.056294	0.050864	0.06448	0.058718
H8	0.057318	0.049159	0.06638	0.060472
H9	0.116442	0.111938	0.110215	0.121045
H18	0.072257	0.068839	0.080857	0.076932
H17	0.058891	0.055767	0.071176	0.060623
H16	0.059023	0.046792	0.071019	0.062094
H15	0.076402	0.071487	0.080271	0.079147
H1a	0.088273	0.111245	0.093833	0.088287
H1b	0.12381	0.07522	0.094955	0.123913
H1c	0.071662	0.06189	0.11969	0.076475
H2a	0.104727	0.107231	0.104487	0.106174
H2b	0.110381	0.097935	0.094412	0.109947
H2c	0.07569	0.056702	0.106688	0.076399

All HAR based models have comparable Bader charges on the atoms in WYLID. Surprisingly, this is also true for the Cu K_{β} data-based model, despite its higher R values, lower multiplicity, lower intensity and larger residuals.

Table S4: Bader charges in electrons for the Mo K_α XRW model based on the different wavelength data sets. Charges are calculated by the *multiwfn* software. (Lu & Chen, 2012)

S1	0.453336	C14	-0.05318	H18	0.106701
O3	-1.28532	C20	1.020904	H15	0.103442
O2	-1.23662	C8	-0.11282	H2a	0.134519
O1	-1.24039	C17	-0.03634	H8	0.095375
C6	-0.05575	C11	0.002501	H1a	0.124056
C4	1.062076	C12	-0.04598	H6	0.108222
C15	-0.03131	C13	1.064963	H2b	0.109136
C5	-0.06658	C1	-0.21979	H1b	0.109289
C7	-0.06788	C2	-0.21738	H17	0.114349
C19	-0.03570	C9	-0.05143	H7	0.098269
C3	-0.24682	C10	-0.02910	H1c	0.155698
C18	-0.09335	H9	0.144558	H2c	0.143070
C16	-0.10524	H16	0.080518		

Table S5: Bader charges in electrons for the MM models based on the different wavelength data sets. Charges are calculated by the *MoPro* software.

	**Mo Kα**	**Synchrotron**
S1	0.708	0.644
O3	-1.04	-0.829
O2	-1.11	-0.852
O1	-1.002	-0.802
C20	0.786	0.782
C11	0.078	0.029
C12	-0.136	-0.168
C19	-0.08	-0.13
C3	-0.07	-0.123
C5	-0.073	-0.035
C9	-0.407	-0.239
C10	-0.146	-0.099
C13	-0.069	-0.041
C14	-0.051	-0.055
C4	0.839	0.769
C6	-0.051	-0.118
C15	-0.101	-0.003
C18	0.001	-0.001
C7	-0.1	-0.093
C1	0.823	0.77
C8	-0.118	-0.3
C17	-0.138	-0.315
C2	-0.05	-0.009
C16	-0.062	-0.077
H6	0.115	0.059
H7	0.068	0.081
H8	0.073	0.073
H9	0.108	0.1

H18	0.093	0.064
H17	0.05	0.038
H16	0.127	0.071
H15	0.097	0.059
H1a	0.129	0.092
H1b	0.086	0.138
H1c	0.141	0.092
H2a	0.139	0.098
H2b	0.079	0.16
H2c	0.124	0.089

S2.4. Laplacians along the C-O bonds compared to 4-heptone

Figure S12 shows the Laplacians of the total electron density from the XRW-Mo K_{α} models compared to a geometry optimized DFT calculation (r2SCAN-3c//r2SCAN/def2-TZVP) of the carbonyl reference structure 4-heptanone and its carbonylate form (r2SCAN/def2-TZVPD//r2SCAN/def2-TZVPD). The difference in basis set was chosen to accommodate for the diffuse character in the anionic enolate/carbonylate.

The carbonyl bonds including O1, close to the SMe_2 group, and O2, the most distant carbonyl to the SMe_2 group, are slightly different from the O3 carbonyl group, which shows slightly less valence shell charge concentration at the oxygen atom and slightly more at the carbon atom.

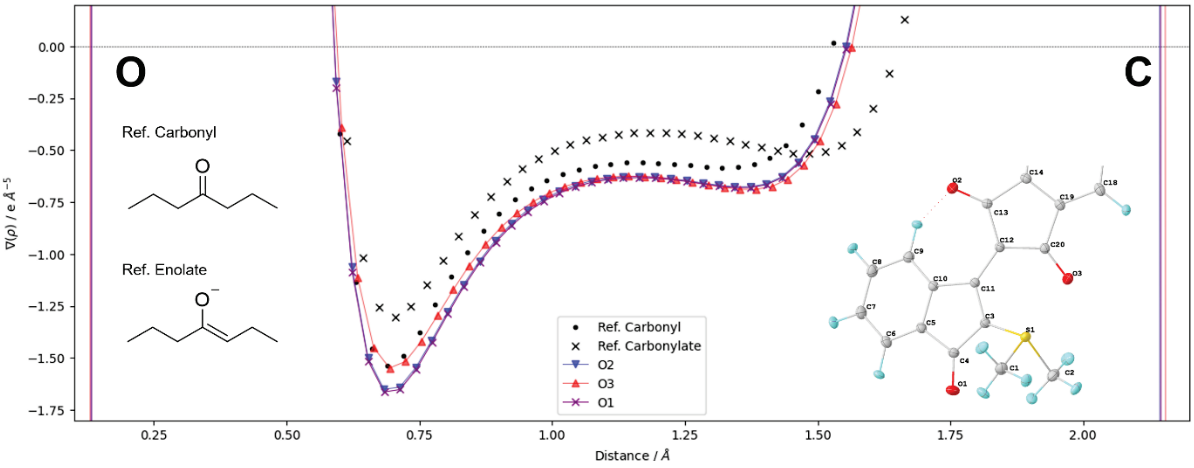

Figure S14: Comparison of the Laplacians of the total electron density along the topological bond path in the three carbonyl groups in WYLID compared to the reference ketone 4-heptanone.

S2.5. Density Functional Theory Calculations of WYLID

Table S6: Geometry after optimization (R2SCAN-3c//R2SCAN/def2-TZVP) for single-point calculation

C	-6.57807	0.930103	-0.42939
C	-6.24253	-0.40103	-0.12085
C	-4.89200	-0.75159	-0.07059
C	-3.91284	0.225512	-0.35214
C	-4.23791	1.535989	-0.67273
C	-5.59709	1.889988	-0.70081
H	-5.89088	2.91713	-0.93956
H	-7.63428	1.216781	-0.45592
H	-3.44834	2.259984	-0.89876
H	-7.02212	-1.13078	0.099336
C	-2.55922	-0.39636	-0.26799
C	-2.81687	-1.79278	0.066871
C	-4.20548	-2.06299	0.152149
C	-4.80538	-3.32877	0.266169
C	-6.15466	-3.67067	0.752079
C	-6.32151	-5.14587	0.534857
C	-5.16612	-5.66145	-0.06501
C	-4.17364	-4.55831	-0.23594
C	-7.40923	-5.96165	0.830095
C	-7.31187	-7.32273	0.499348
C	-6.15380	-7.83909	-0.10522
H	-8.30777	-5.54605	1.29727
H	-8.14993	-7.99417	0.71269
C	-5.06050	-7.00887	-0.39858
H	-6.10604	-8.90540	-0.34879
H	-4.15227	-7.40042	-0.86757
O	-7.00209	-2.95932	1.282293
O	-3.07213	-4.69517	-0.77255
O	-1.48000	0.171573	-0.44005
S	-1.56320	-2.89840	0.549637
C	-0.52291	-1.88726	1.657175
H	0.35112	-2.50275	1.922197
H	-1.12576	-1.68673	2.555835
H	-0.22972	-0.95217	1.160463
C	-0.46891	-3.00148	-0.90026
H	-1.03729	-3.55420	-1.66040
H	0.431913	-3.56069	-0.60309
H	-0.22352	-1.98082	-1.22854

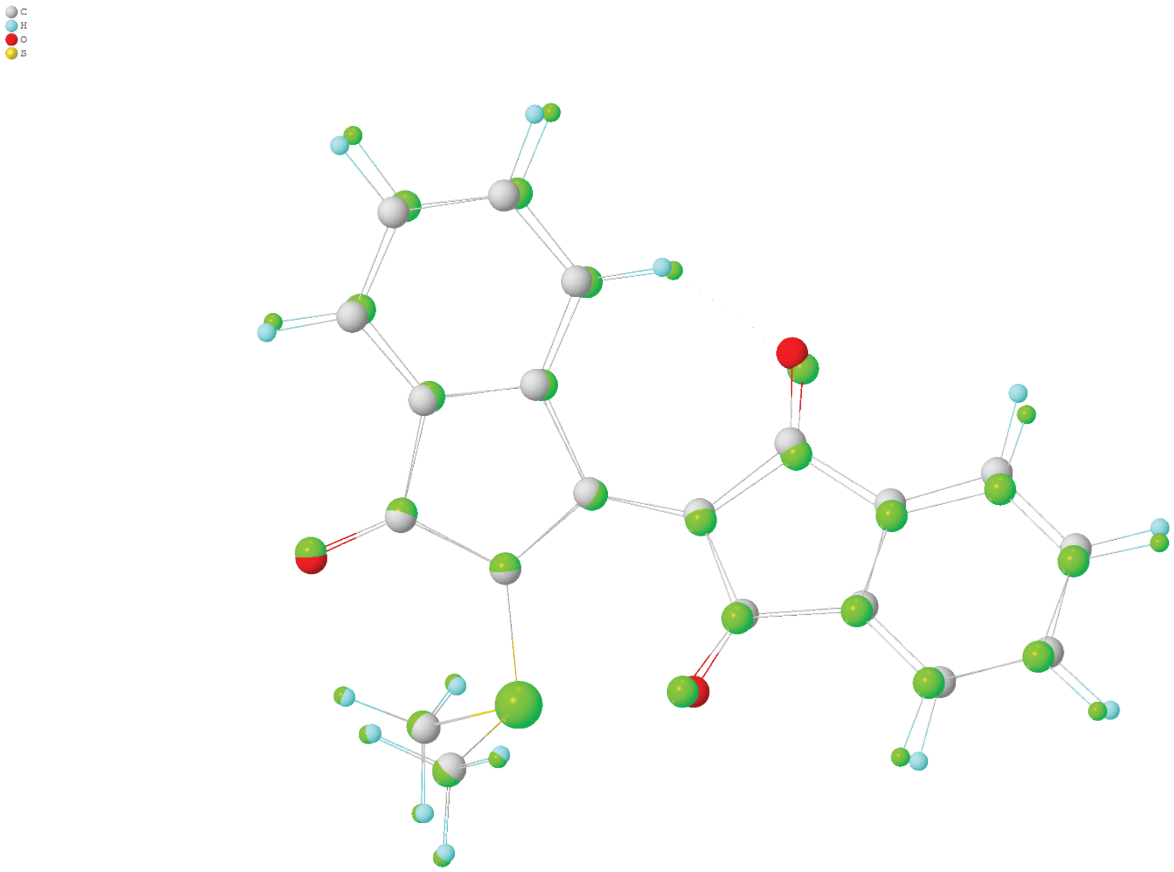

Figure S15: Overlay of the optimized geometry (green) of WYLID and the one obtained in the HAR-Mo K_α model.

The comparison of the optimized geometry (r2SCAN-3c) of WYLID and the HAR-Mo K_α model from single crystal X-ray diffraction in Fig. S15 shows a close similarity between both geometries. This is consistent with similar charges in the quantum crystallographic evaluation.

Table S7: Bader charges of the DFT calculation of WYLID.

C8	-0.0962
C9	-0.02955
C10	-0.02184
C5	-0.07083
C6	-0.09984
C7	-0.10109
H8	0.125476
H7	0.11177
H6	0.147795
H9	0.117614
C4	0.982127
C3	-0.24394
C11	0.028121
C12	-0.09113
C13	1.000304
C14	-0.05188
C19	-0.06447
C20	0.920088
C15	-0.03008
C16	-0.04121
C17	-0.06941
H15	0.082195
H16	0.064142
C18	-0.08857
H17	0.094581
H18	0.142043
O2	-1.1751
O3	-1.12001
O1	-1.14241
S1	0.40022
C1	-0.17759
H1A	0.08441
H1B	0.093117
H1C	0.125157
C2	-0.26356
H2A	0.182628
H2B	0.098057
H2C	0.17886

S2.6. Comparison of saturated and unsaturated detector synchrotron data sets

The saturation of a modern dual-pixel photon-counting detector, such as the PILATUS3 X 2M, occurs when the number of photons incident upon the detector exceeds the capacity of the electronic readout for each pixel to process. Accordingly, the observed intensity is less than what would be anticipated. This indicates that particularly high-intensity reflections in a diffraction experiment may be influenced by oversaturation, whereas low-intensity reflections are still measured to their full extent.

The data set for the detector saturation can be found in the CSD (2401360 and as "data_Ylideanhydrate-20000eV" data set in this publication) and the raw data with additional models are available at via zenodo.org (DOI: **10.5281/zenodo.14725036**).

Figure S16 shows the measured structure factors F_{obs} versus the calculated structure factors F_{calc}. The result for oversaturation in this plot looks similar to strong multiple scattering events, such as extinction, and can be corrected to some extent by an extinction correction (Sheldrick, 2015). The main difference is that reflections suffering from oversaturation are strongly dispersed compared to classical extinction, where reflections should all fall on the F_{obs} / F_{calc} diagonal.

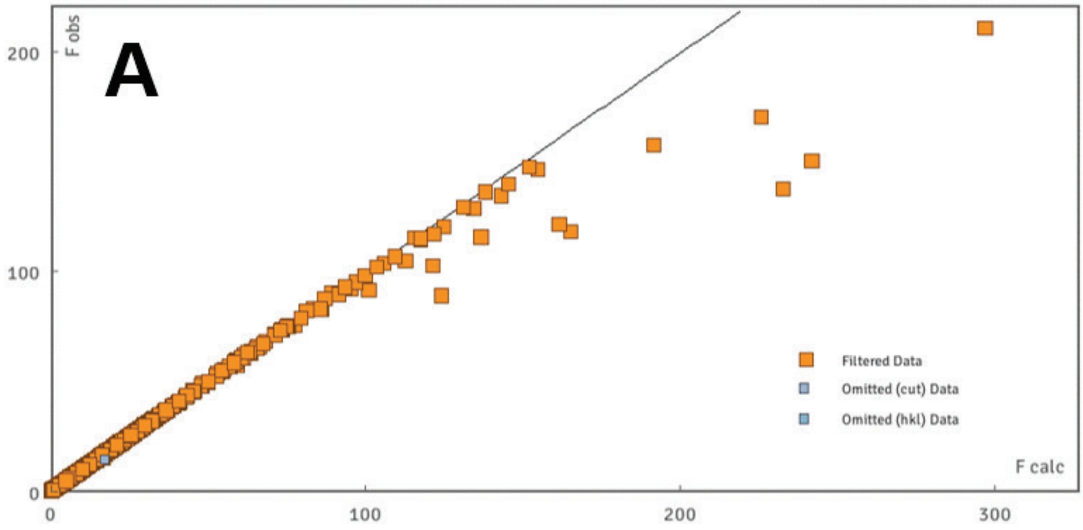

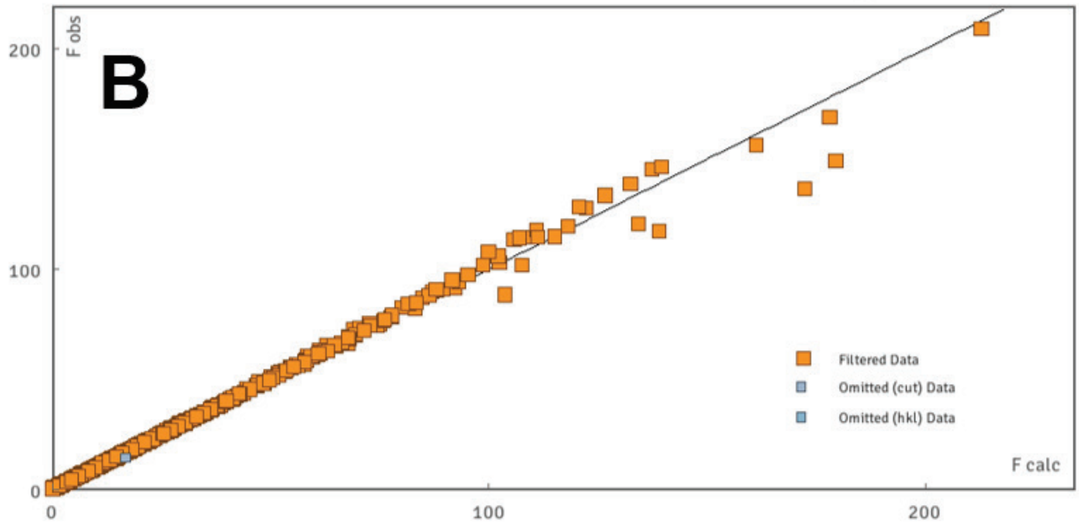

Figure S16: Comparison of the F_{obs} / F_{calc} plot exhibiting detector saturation for the strong reflection before (A) and after (B) extinction correction.

Table S8: Bader charges with of the reference model at 22 keV compared to the model of the data set at 20 keV exhibiting detector oversaturation with (“EXTI”) and without (“NoExti”) applied extinction correction as well as their differences to the reference model.

	**22keV**	**NoExti**	**20 keV-NoExti**	**EXTI**	**20 keV-EXTI**
S1	0.464	0.469	-0.005	0.469	-0.006
O3	-1.174	-1.178	0.004	-1.178	0.004
O1	-1.181	-1.186	0.005	-1.186	0.005
O2	-1.170	-1.176	0.006	-1.175	0.006
C13	0.988	0.993	-0.006	0.994	-0.006
C10	-0.028	-0.038	0.010	-0.029	0.001
C4	0.984	0.025	0.959	0.990	-0.006
C9	-0.029	-0.034	0.005	-0.024	-0.005
C14	-0.050	-0.029	-0.021	-0.048	-0.002
C11	0.024	-0.026	0.050	0.024	0.001
C19	-0.051	-0.026	-0.025	-0.052	0.001
C5	-0.047	-0.084	0.037	-0.047	-0.001
C3	-0.237	-0.241	0.004	-0.241	0.004
C15	-0.025	-0.046	0.021	-0.028	0.002
C18	-0.031	-0.038	0.007	-0.026	-0.005
C12	-0.085	-0.049	-0.036	-0.085	0.000
C1	-0.160	-0.150	-0.010	-0.150	-0.010
C6	-0.030	0.962	-0.993	-0.026	-0.004
C8	-0.044	-0.024	-0.020	-0.039	-0.005
C2	-0.141	-0.140	-0.001	-0.140	-0.002
C7	-0.035	-0.052	0.016	-0.029	-0.006
C17	-0.034	-0.028	-0.006	-0.033	-0.002
C16	-0.040	-0.025	-0.016	-0.035	-0.005
C20	0.958	0.989	-0.031	0.962	-0.004
H6	0.076	0.070	0.006	0.071	0.005
H7	0.059	0.055	0.004	0.052	0.006
H8	0.060	0.052	0.008	0.056	0.005
H9	0.121	0.118	0.003	0.117	0.004
H18	0.077	0.079	-0.002	0.071	0.006
H17	0.061	0.061	0.000	0.059	0.002
H16	0.062	0.060	0.002	0.060	0.003
H15	0.079	0.071	0.008	0.078	0.001
H1a	0.088	0.087	0.002	0.086	0.002
H1b	0.124	0.123	0.000	0.123	0.001
H1c	0.076	0.069	0.008	0.068	0.008
H2a	0.106	0.103	0.003	0.104	0.002
H2b	0.110	0.111	-0.001	0.110	0.000
H2c	0.076	0.075	0.002	0.075	0.001
**SUM**	-2E-06	-3E-05	3.1E-05	0.000	1.8E-16

In general, the errors introduced into the HAR model of the synchrotron data by the detector saturation are small. When an extinction correction is applied, the difference is almost imperceptible.

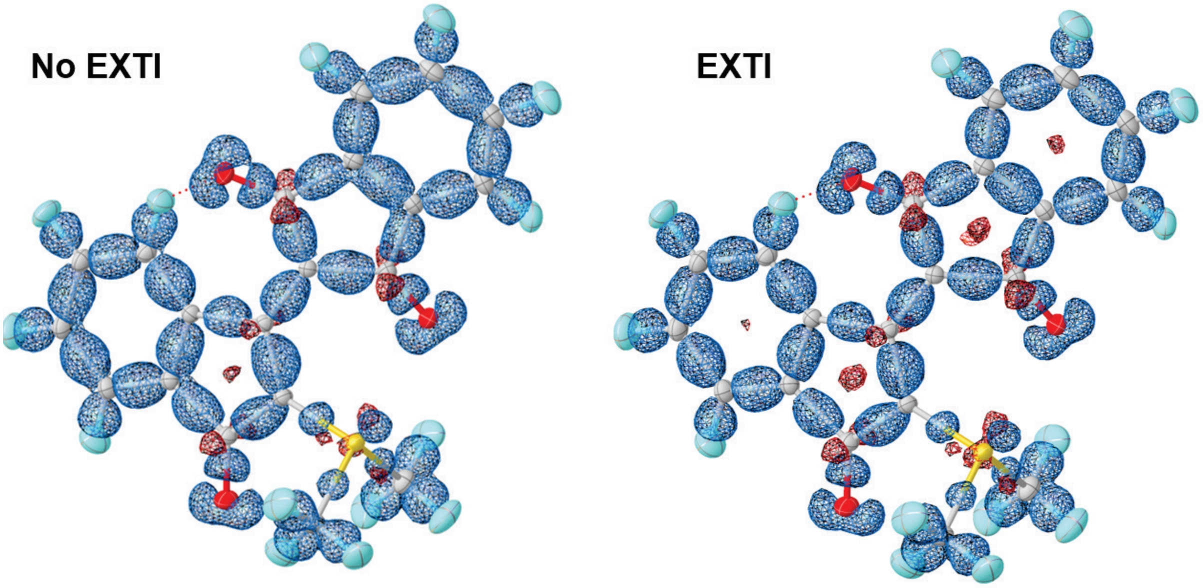

Figure S17: Deformation density at the 0.15 *e*/Å^3 iso level of WYLID using the detector saturating data set of WYLID. Blue indicates positive, red negative deformation density.

While the deformation density is similar in both cases, fine features such as the π-holes of the aromatic ring systems are absent or less pronounced without extinction correction. This suggests that extinction correction improves the quantum crystallographic models when the detector is oversaturated and could generally be applied *a posteriori*. However, saturation of the detector should be avoided by attenuating the beam as much as possible.

S2.7. Comparison of HAR results between r2SCAN and α B97X

Table S9: Comparison of Bader charges between the HAR-synchrotron models using either the meta GGA functional r2SCAN or the hybrid function ωB97X. In both cases, the def2-TZVP basis sets were used.

Level of DFT	**r2SCAN**	α **B97X**
Formula	$C_{20}H_{14}O_3S$	
Mass / g mol^{-1}	334.40	
Crystal size / mm^3	0.1 x 0.1 x 0.1	
Space group	*Pbca*	
a / Å	14.54072(7)	
b / Å	9.17361(2)	
c / Å	23.40310(7)	
V / Å^3	3121.7(2)	
D / g cm^{-3}	1.423	
μ / mm^{-1}	0.122	
θ range / °	2.24-40.40	
T / K	100	
Resolution / Å	0.55	
I/σ(*I*)	95.3	
R_{int} / %	4.88	
Total reflections	508696	
Unique reflections > 4σ	8745	
Completeness / %	100.0	
	**Model Indicators**	
Nr. refined parameters	444	444
Goodness-of-fit	1.070	1.051
R indices, *I* > 2σ / %	1.00	1.01
R indices (all data) / %	1.39	1.42
wR2 indices / %	1.83	1.95
Diff. peak, hole / e Å^{-3}	0.106, -0.117	0.105, -0.202

The resulting structures from HAR on r2SCAN and ωB97X were identical to the degree that we could not spot any parts of the structure that were not completely overlaid.

Table S10: Comparison of Bader charges between the HAR-synchrotron models using either the meta GGA functional r2SCAN or the hybrid function ωB97X. In both cases, the def2-TZVP basis sets were used.

	**r2SCAN**	**ωB097X**	**r2SCAN - ωB97X**
S1	0.464	0.438	0.026
O3	-1.174	-1.206	0.033
O1	-1.181	-1.207	0.026
O2	-1.170	-1.198	0.028
C13	0.988	1.012	-0.024
C10	-0.028	-0.028	0.000
C4	0.984	1.012	-0.028
C9	-0.029	-0.022	-0.007
C14	-0.050	-0.053	0.003
C11	0.024	0.034	-0.009
C19	-0.051	-0.054	0.003
C5	-0.047	-0.049	0.002
C3	-0.237	-0.238	0.001
C15	-0.025	-0.019	-0.006
C18	-0.031	-0.026	-0.005
C12	-0.085	-0.099	0.014
C1	-0.160	-0.125	-0.035
C6	-0.030	-0.022	-0.008
C8	-0.044	-0.038	-0.006
C2	-0.141	-0.109	-0.032
C7	-0.035	-0.031	-0.005
C17	-0.034	-0.028	-0.006
C16	-0.040	-0.036	-0.005
C20	0.958	0.980	-0.022
H6	0.076	0.073	0.003
H7	0.059	0.056	0.003
H8	0.060	0.057	0.003
H9	0.121	0.119	0.002
H18	0.077	0.074	0.002
H17	0.061	0.054	0.006
H16	0.062	0.056	0.006
H15	0.079	0.076	0.004
H1a	0.088	0.082	0.006
H1b	0.124	0.119	0.005
H1c	0.076	0.071	0.006
H2a	0.106	0.100	0.006
H2b	0.110	0.105	0.005
H2c	0.076	0.070	0.006

To estimate the influence of the choice of the functional used in the HAR procedure a second HAR was performed using the synchrotron data set at 22 keV. We chose to compare the meta-GGA functional r2SCAN with the hybrid functional ωB97X, which includes part of a Hartree-Fock calculation. The incorporation of Hartree-Fock into the HAR procedure resulted in a greater overall charge on the S and O atoms, as well as on the carbonyl C atoms.

S2.8. CSD search using MOGUL

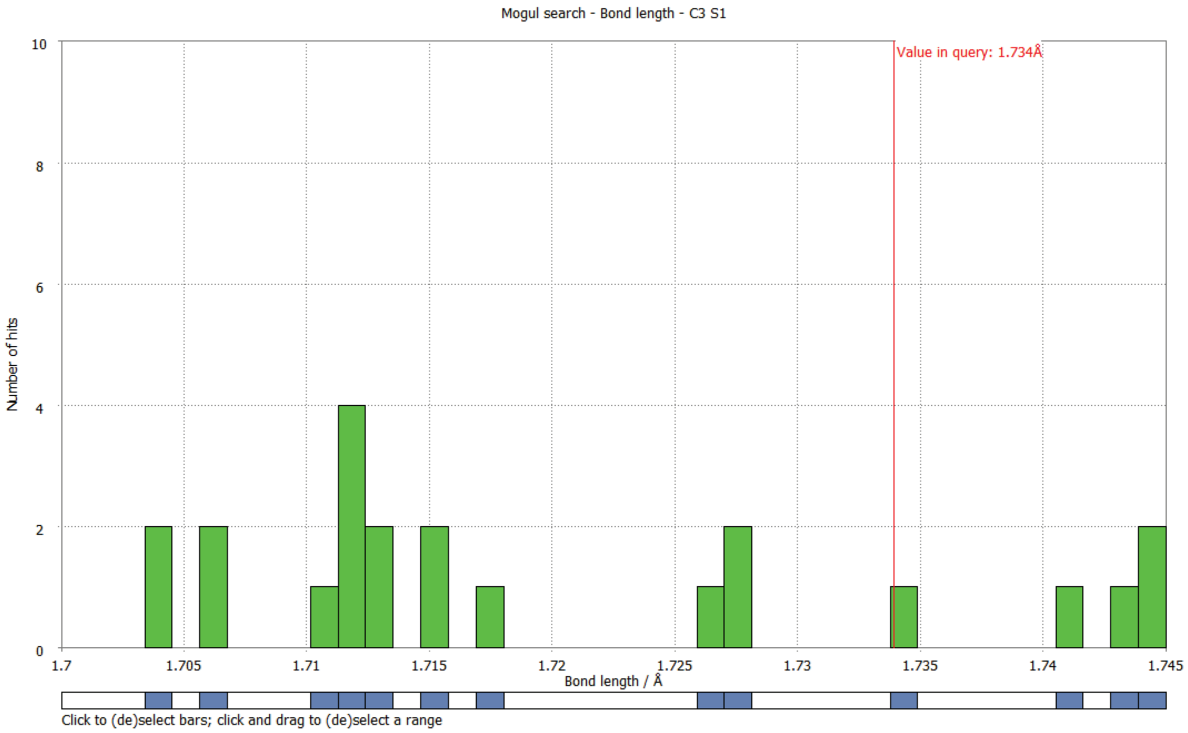

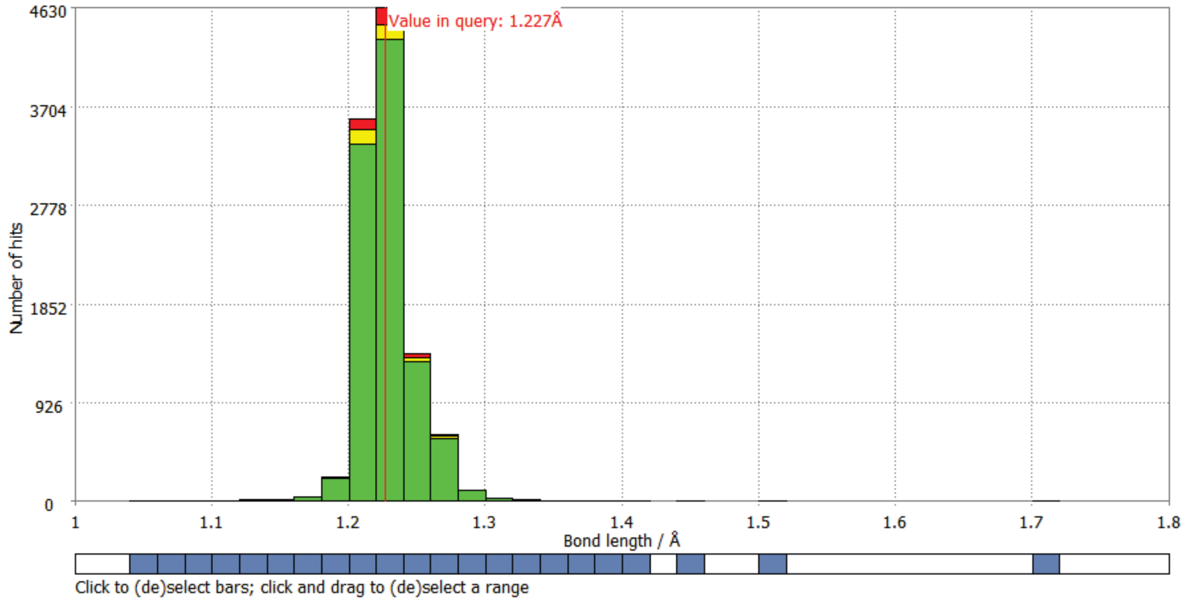

Figure S18: Results of the MOGUL search for the S1-C3 (top, 23 hits) and O1-C4 (bottom, 10670 hits) bond lengths of the HAR-Mo K_{α} structure compared to similar structures.

S2.9. Hirshfeld Surface Analysis of WYLID

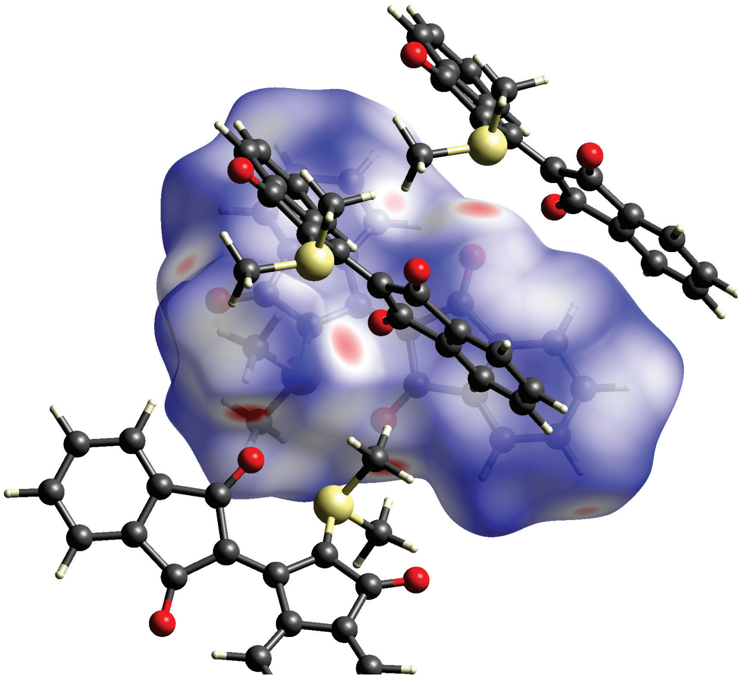

Figure S19: Hirshfeld surface at an iso level of 0.5 of WYLID with closest contacts imprinted on its surface and the closest interacting symmetry equivalents.

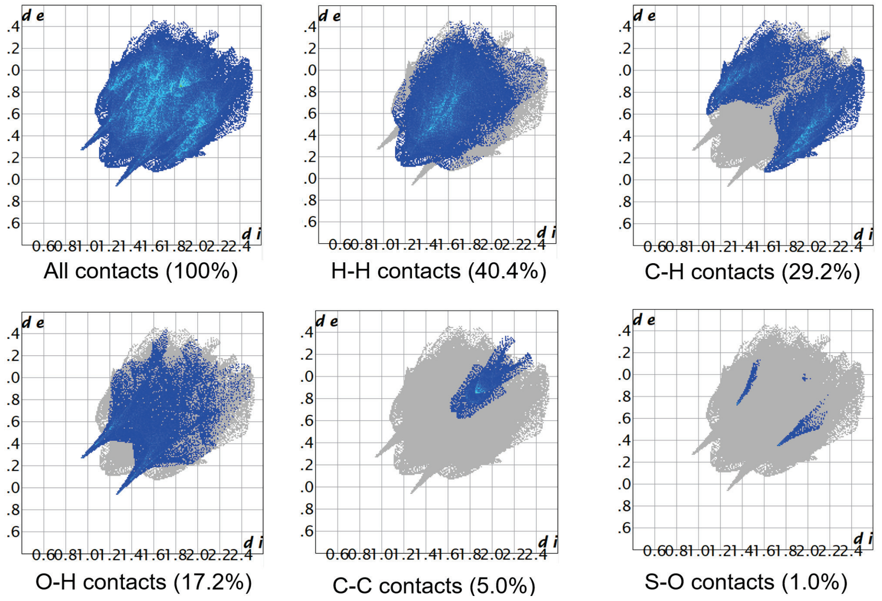

Figure S20: Fingerprint plots of the closest contacts of WYLID on the Hirshfeld surface.

The Hirshfeld surface mapped with d_{norm} (Fig. S19) shows the closest intermolecular contacts in the crystal packing of WYLID. The interactions are predominantly of the O...H and O...S type. While the latter is one of the closest contacts, its total contribution to all interactions is quite small, accounting for only 1% of the contacts (Fig. S20). The main interactions are of the H...H and C...H type.

S2.10. Anomalous Dispersion Refinement

Table S11: Anomalous dispersion parameters from different sources for S at Cu K_α excitation wavelength compared to experimental values obtained by ADR in Olex2. (Meurer *et al.*, 2022)

	f' /*e*	f''/*e*
WYLID HAR-Cu K_α	0.36(1)	0.69(2)
YLID Balmohammadi *et al*. (2025), Avg.	0.27(5)	0.58(4)
Henke *et al.* (1982)	0.3354	0.5514
Sasaki (1989)	0.328	0.590
Brennan & Cowan (1992)	0.3338	0.5584

Table S11 shows the values obtained by ADR compared to those of Balmohammadi *et al.* (2025) over nine different data sets and temperatures. Interestingly, while f'' is closer to the tabulated values and f' differs more in the Balmohammadi et al. data sets, the opposite is true for our measurement on WYLID.

S2.11. Crystallographic Details of Bindandione

Table S12: Crystallographic data of the Bindandione data set as well as the quality indicators of its HAR model.

Formula	$C_{36}H_{20}O_6$
Mass / g mol-1	548.556
Crystal size / mm3	0.1 x 0.1 x 0.1
Space group	*Fdd2*
a / Å	18.0174(1)
b / Å	20.922(2)
c / Å	26.0847(2)
V / Å3 /	9832.94(13)
D / g cm-3	1.482
µ / mm-1	0.827
θ range / °	3.65 – 75.32
T / K	100.0(1)
Resolution / Å	0.80
I/σ(I)	85.2
Rint / %	4.52
Total reflections	102230
Unique reflections > 4σ	5027
Completeness / %	100.0
**Quality Indicators**	
Nr. refined parameters	559
Goodness-of-fit	1.079
R indices, I > 2σ / %	1.60
R indices (all data) / %	1.63
wR2 indices / %	3.67
Diff. peak, hole / e Å-3	0.096, -0.090
Hooft	0.00(3)

S3. References

Balmohammadi, Y., Malaspina, L. A., Nakamura, Y., Cametti, G., Andrzejewski, M., Siczek, M. & Grabowsky, S. (2025). *Sci. Rep.* In the press.

Bourhis, L. J., Dolomanov, O. V., Gildea, R. J., Howard, J. A. K. & Puschmann, H. (2015). *Acta Cryst.* A**71**, 59–75.

Brennan, S. & Cowan, P. L. (1992). *Rev. Sci. Instrum.* **63**, 850–853.

Capelli, S. C., Bürgi, H.-B., Dittrich, B., Grabowsky, S. & Jayatilaka, D. (2014). *IUCrJ* **1**, 361–379.

Dolomanov, O. V., Bourhis, L. J., Gildea, R. J., Howard, J. A. K. & Puschmann, H. (2009). *J. Appl. Cryst.* **42**, 339–341.

Furness, J. W., Kaplan, A. D., Ning, J., Perdew, J. P. & Sun, J. (2020). *J. Phys. Chem. Lett.* **11**, 8208–8215.

Gasevic, T., Stückrath, J. B., Grimme, S. & Bursch, M. (2022). *J. Phys. Chem. A* **126**, 3826–3838.

Guillot, B. (2012). *Acta Cryst.* A**68**, 204–204.

Henke, B. L., Gullikson, E. M. & Davis, J. C. (1993). *At. Data Nucl. Data Tables* **54**, 181–342.

Hirshfeld, F. L. (1977). *Theoret. Chim. Acta* **44**, 129–138.

Jayatilaka, D. & Grimwood, D. J. (2003). Vol. *2660*, *Computational Science — ICCS 2003*, edited by P. M. A. Sloot, D. Abramson, A. V. Bogdanov, Y. E. Gorbachev, J. J. Dongarra & A. Y. Zomaya. pp. 142–151. Berlin, Heidelberg: Springer Berlin Heidelberg.

Jelsch, C., Guillot, B., Lagoutte, A. & Lecomte, C. (2005). *J. Appl. Cryst.* **38**, 38–54.

Kleemiss, F., Dolomanov, O. V., Bodensteiner, M., Peyerimhoff, N., Midgley, L., Bourhis, L. J., Genoni, A., Malaspina, L. A., Jayatilaka, D., Spencer, J. L., White, F., Grundkötter-Stock, B., Steinhauer, S., Lentz, D., Puschmann, H. & Grabowsky, S. (2021). *Chem. Sci.* **12**, 1675–1692.

Lu, T. & Chen, F. (2012). *J. Comput. Chem.* **33**, 580–592.

Meindl, K. & Henn, J. (2008). *Acta Cryst.* A**64**, 404–418.

Meurer, F., Dolomanov, O. V., Hennig, C., Peyerimhoff, N., Kleemiss, F., Puschmann, H. & Bodensteiner, M. (2022). *IUCrJ* **9**, 604–609.

Neese, F., Wennmohs, F., Becker, U. & Riplinger, C. (2020). *J. Chem. Phys.* **152**, 224108.

Rigaku Oxford Diffraction (2024). *CrysAlisPro Software System*, Wroclaw, Poland

Sasaki, S. (1989). KEK Report 88–14, pp. 1–136. Ibaraki-keri, Japan: National Laboratory for High Energy Physics.

Sheldrick, G. M. (2015). *Acta Cryst.* A**71**, 3–8.

Weigend, F. & Ahlrichs, R. (2005). *Phys. Chem. Chem. Phys.* **7**, 3297.
